# Synthesis, biological evaluation, and molecular docking of new series of antitumor and apoptosis inducers designed as VEGFR-2 inhibitors

**DOI:** 10.1080/14756366.2021.2017911

**Published:** 2022-01-10

**Authors:** Abdallah E. Abdallah, Reda R. Mabrouk, Maged Mohammed Saleh Al Ward, Sally I. Eissa, Eslam B. Elkaeed, Ahmed B. M. Mehany, Mariam A. Abo-Saif, Ola A. El-Feky, Mohamed S. Alesawy, Mohamed Ayman El-Zahabi

**Affiliations:** a Pharmaceutical Medicinal Chemistry & Drug Design Department, Faculty of Pharmacy (Boys), Al-Azhar University, Cairo, Egypt; b Department of Pharmaceutical Chemistry, Faculty of Pharmacy (Girls), Al-Azhar University, Cairo, Egypt; c Department of Pharmaceutical Sciences, College of Pharmacy, AlMaarefa University, Riyadh, Saudi Arabia; d Zoology Department, Faculty of Science, Al-Azhar University, Cairo, Egypt; e Biochemistry Department, Faculty of Pharmacy, Tanta university, Tanta, Egypt

**Keywords:** Anticancer, apoptosis, multi-kinase, pharmacophoric features, VEGFR-2

## Abstract

Based on quinazoline, quinoxaline, and nitrobenzene scaffolds and on pharmacophoric features of VEGFR-2 inhibitors, 17 novel compounds were designed and synthesised. VEGFR-2 IC_50_ values ranged from 60.00 to 123.85 nM for the new derivatives compared to 54.00 nM for sorafenib. Compounds **15_a_**, **15_b_**, and **15_d_** showed IC_50_ from 17.39 to 47.10 µM against human cancer cell lines; hepatocellular carcinoma (HepG2), prostate cancer (PC3), and breast cancer (MCF-7). Meanwhile, the first in terms of VEGFR-2 inhibition was compound **15_d_** which came second with regard to antitumor assay with IC_50_ = 24.10, 40.90, and 33.40 µM against aforementioned cell lines, respectively. Furthermore, Compound **15_d_** increased apoptosis rate of HepG2 from 1.20 to 12.46% as it significantly increased levels of Caspase-3, BAX, and P53 from 49.6274, 40.62, and 42.84 to 561.427, 395.04, and 415.027 pg/mL, respectively. Moreover, **15_d_** showed IC_50_ of 253 and 381 nM against HER2 and FGFR, respectively.

## 1. Introduction

Cancer is a life-threatening disease and is reported as a leading cause of death worldwide, accounting for approximately 10 million deaths in 2020^1^. It was reported that non-selective chemotherapeutic agents cause toxicity to normal cells^2^^,^^3^. These two points clearly reveal the need to develop new potent anticancer agents with relatively better selectivity.

Cancer cells are characterised by biochemical abnormalities rather than normal cells. We suggest that development of anticancer agents should primarily be directed to address such defects. One of these specific features of cancer cells is the abnormality of tyrosine kinases. Tyrosine kinases are important enzymes that play a pivotal role in signal transduction, cell survival, proliferation, and migration^4^.

Tyrosine kinases are differentiated into two types: receptor tyrosine kinases (RTKs) and non-receptor tyrosine kinases (NRTKs)^5^. Twenty different types of RTKs have been recognised^3^. They primarily consist of insulin-like receptor and growth factor receptor such as VEGFR, PDGFR, EGFR, FGFR, and NGFR^6^. Among the targets of RTKs, vascular endothelial growth factor receptors (VEGFRs) have attracted attention of researchers as excellent targets to develop new anticancer agents^7^^,^^8^.

VEGF family includes five factors, namely VEGFA, VEGFB, VEGFC, VEGFD, and placental growth factor (PLGF)^9^. Many types of tumour have shown overexpression of VEGFs and their receptors^10^. Accordingly, high rate of angiogenesis is obtained to provide nutrition and oxygen required for tumour growth^9^. Regarding VEGF receptors, they are categorised into three types: VEGFR-1, VEGFR-2, and VEGFR-3^11^. In physiological and pathological angiogenesis, VEGFR-1 and VEGFR-2 play an essential role. While VEGFR-3 is associated with embryogenesis and lymphangiogenesis^12^^,^^13^.

**Figure 1.**
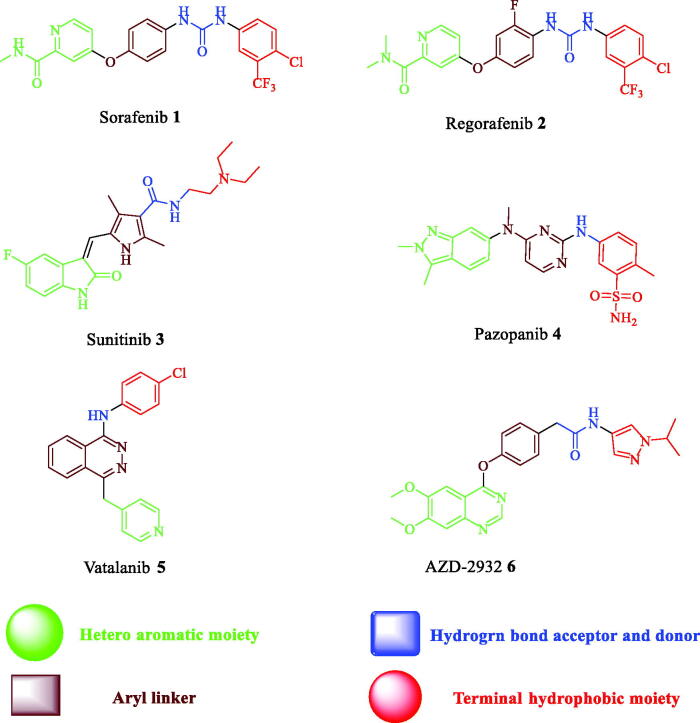
Structures of some reported VEGFR-2 kinase inhibitors.

An essential receptor for VEGF-dependent angiogenesis is VEGFR-2 which binds primarily all VEGF-A isoforms^9^. There is some evidence that metastasis of many solid tumours is attributed to overexpression of VEGFR-2^14^^,^^15^. It was reported that tumour growth can be efficiently inhibited by blocking of angiogenesis^16^. Hence, VEGFR-2 inhibition is an efficient approach to obtain effective anticancer agents^17^. There are some FDA-approved VEGFR-2 inhibitors such as Sorafenib **1**^18^, Regorafenib **2**^19^, Sunitinib **3**^20^, Pazopanib **4**^21^, and Vatalanib **5**^22^ (Figure 1).

VEGFR-2 inhibitors are divided into two major types^23^. Type I inhibitors are characterised by their ability to accommodate the region, of the active conformation of the receptor that primarily occupied by the adenine moiety of ATP^24^. While type II kinase inhibitors are known to occupy a hydrophobic allosteric site appeared just close to the ATP-binding domain in the inactive conformation (DFG-out) of the enzyme^25^. Type-II inhibitors such as Sorafenib outweigh type-I inhibitors by many advantages such as relatively higher selectivity to the enzyme as well as slower off-rates^26^.

### 1.1. Rationale and design

**Figure 2.**
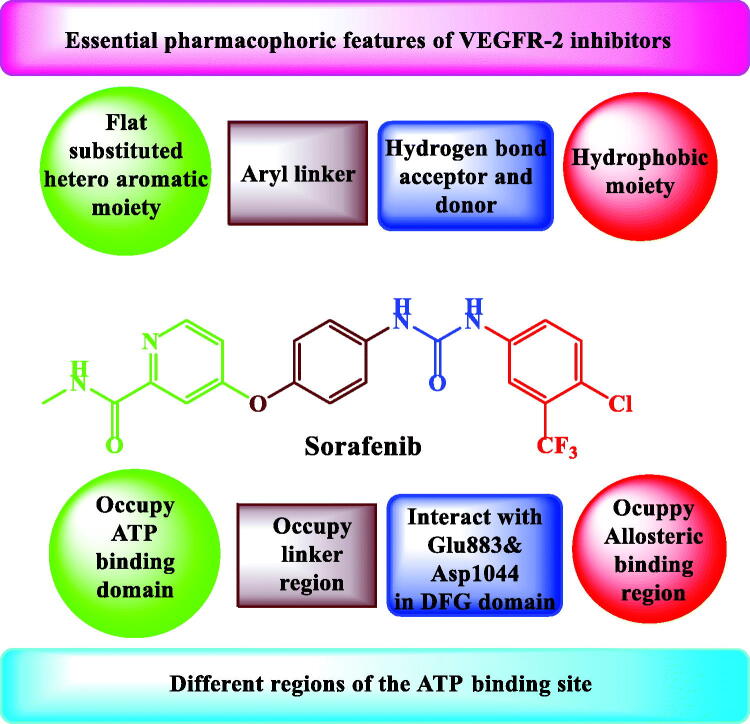
Sorafenib as a model for pharmacophoric features of type II VEGFR-2 kinase inhibitors.

Type II VEGFR-2 inhibitors have common four pharmacophoric features on which the design of the new derivatives of the current work was based for the present work. Figure 2 illustrates the four regions that were reported to be occupied by these features^27^^,^^28^. We can see that the ATP-binding domain (Hinge region) is accommodated by a flat heteroaromatic moiety. The interaction between the heteroaromatic moiety and Glu917 and/or Cys919 residues in this region is important^6^^,^^29^^,^^30^. Secondly, the area between ATP-binding domain and DFG domain requires a linker with suitable length to fit^31^. Thirdly, Glu885 and Asp1046 residues in the DFG domain are essential residues to which the hydrogen bond acceptors and donors interact^29^. Finally, there are hydrophobic interactions between the allosteric region of the inactive confirmation of the receptor and the terminal hydrophobic group^29^^,^^32^.

**Figure 3.**
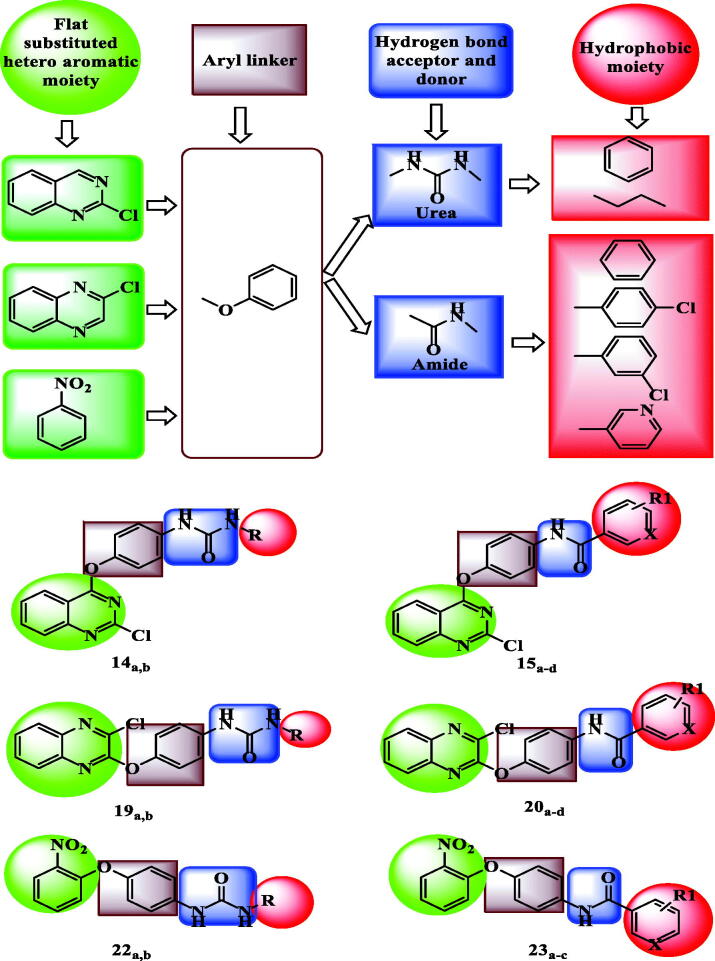
Summary for the rationale of molecular design showing that, the target compounds fulfilled the pharmacophoric features of VEGFR-2 inhibitors.

Figure 3 shows a model for the designed compounds and demonstrates the consistency of their structures with the pharmacophoric features that reported to type II VEGFR-2 inhibitors. With respect to the flat heteroaromatic moiety, three nuclei were taken into account in our design: quinazoline, quinoxaline, and nitrobenzene. AZD-2932 **6** (IC_50_ = 8 nM)^33^ is a quinazoline-based VEGFR-2 inhibitor (Figure 1). It clearly reveals the significance of quinazoline as flat heteroaromatic moiety in VEGFR-2 inhibitors. As a bioisoster to quinazoline, quinoxaline was also designed to study the effect of changing benzodiazine nitrogen positions on the activity. 2-Nitrobenzene was also designed in this work because that benzene is an isostere to pyridine of Sorafenib **1** and Regorafenib **2** (Figure 1). It will show to what extent heterocycle is important for activity. Additionally, there is a nitro group at position 2 which may interact with the essential residues Glu917 and/or Cys919 in Hinge region.

Urea as well as amide moiety was found to be effective hydrogen bond acceptors and donors in some VEGFR-2 inhibitor drugs as Regorafenib and Sunitinib, respectively (Figure 1). So that urea and amide moieties were designed in this work as hydrogen bond acceptors and donors. We aimed to study which moiety will be of more significant effect.

Herein, the phenoxy group was designed as an aryl linker in all new derivatives. This group was selected because it was an effective linker in some VEGFR-2 inhibitor drugs as Sorafenib, Regorafenib, and AZD-2932 **6** (Figure 1).

Finally, the terminal hydrophobic moiety was planned to be phenyl, 3-chlorophenyl, 4-chlorophenyl as well as pyridyl. Furthermore, butyl as aliphatic group was introduced to test whether the aliphatic hydrophobic moiety could play a good substitute for the aromatic hydrophobic groups or not.

## 2. Materials and methods

### 2.1. Chemistry

All melting points were obtained by open capillary method on a Gallen lamp Melting point apparatus and were uncorrected. Infra-red spectra were recorded on Pye Unicam SP 1000 IR spectrophotometer (KBr discs) and were expressed in wave number (cm^–1^). ^1^H NMR and ^13^C NMR spectra were recorded on a BRUKER 400 MHZ-NMR spectrophotometer. TMS was used as internal standard in deuterated DMSO, and chemical shifts were measured in δ ppm. Elemental analyses were performed on a PerkinElmer 2400 series II CHN elemental analyser. Progresses of the reaction was monitored by TLC using TLC sheets precoated with UV fluorescent silica gel Merck 60 F254 plates and were visualised using UV lamp.

#### 2.1.1. General method for synthesis of compounds (14_a&b_) and (15_a–d_)

Equimolar amounts of 2,4-dichloroquinazoline (0.12 g, 0.60 mmol) and the appropriate intermediate (**8_a&b_** and **10_a–d_**) were mixed with K_2_CO_3_ (0.10 g, 0.72 mmol) in isopropanol. The reaction mixture was refluxed for about 1 h. Upon cooling, the obtained precipitate was collected by filtration and dried. Then, it was washed by water, dried, and recrystallized from isopropanol.

##### 2.1.1.1. 1-(4-((2-Chloroquinazolin-4-yl)oxy)phenyl)-3-phenylurea (14_a_)

White solid: (yield: 0.19 g, 81.35%); m.p. = 236–237 °C; IR (KBr, cm^–1^): 3313, 3294, 3213, 3147 (NH), 3062 (C–H aromatic), 1658 (CO urea), 1616 (C = N), 1600, 1558, 1489, 1442 (C = C aromatic), 1207 (C–O); ^1^H NMR (DMSO-d_6_, 400 MHz) *δ* (ppm): 6.99 (t, *J* = 7.2 *Hz*, 1H, Ar-H), 7.30 (m, 4H, Ar-H), 7.48 (d, *J* = 7.8 *Hz*, 2H, Ar-H), 7.58 (d, *J* = 8.9 *Hz*, 2H, Ar-H), 7.81 (ddd, *J* = 8.2, 7.0, 1.2 *Hz*, 1H, Ar-H quinazoline), 7.94 (d, *J* = 8.4 *Hz*, 1H, Ar-H quinazoline), 8.09 (ddd, *J* = 8.5, 7.0, 1.5 *Hz*, 1H, Ar-H quinazoline), 8.39 (dd, *J* = 8.2, 1.5 *Hz*, 1H, Ar-H quinazoline), 8.73 (s, 1H, D_2_O exchangeable, NH), 8.82 (s, 1H, D_2_O exchangeable, NH). ^13 ^C NMR (DMSO-d_6_, 100 MHz) *δ* (ppm): 114.91, 118.73, 119.82, 122.37, 122.53, 124.49, 127.12, 128.86, 129.27, 136.27, 138.29, 140.11, 146.59, 152.61, 153.07, 155.16, 168.52. Anal. Calcd. for C_21_H_15_ClN_4_O_2_ (390.83): C, 64.54; H, 3.87; N, 14.34. Found: C, 64.72; H, 4.09; N, 14.25%.

##### 2.1.1.2. 1-Butyl-3-(4-((2-chloroquinazolin-4-yl)oxy)phenyl)urea (14_b_)

Brown solid: (yield: 0.16 g, 73.80%); m.p. = 214–215 °C; IR (KBr, cm^–1^): 3329, 3116 (NH), 3062, 3042 (C–H aromatic), 2983, 2931, 2870 (C–H aliphatic), 1643 (CO urea), 1616 (C = N), 1554, 1489, 1446 (C = C aromatic), 1207 (C–O); ^1^H NMR (DMSO-*d*_6_, 400 MHz) δ (ppm): 0.90 (t, *J* = 7.3 *Hz*, 3H, CH_3_), 1.31 (m, 2H, *CH_2_*CH_3_), 1.41 (m, 2H, *CH_2_*CH_2_CH_3_), 3.10 (dt, *J* = 6.5 *Hz*, 2H, *CH_2_*NH), 6.19 (t, *J* = 5.7 *Hz*, 1H, *NH*CH_2_), 7.22 ( d, *J* = 8.8 *Hz*, 2H, Ar-H), 7.50 (d, *J* = 8.8 *Hz*, 2H, Ar-H), 7.79 (dd, *J* = 7.6 *Hz*, 1H, Ar-H quinazoline), 7.91 (d, *J* = 8.4 *Hz*, 1H, Ar-H quinazoline), 8.07 (dd, *J* = 7.4, 7.7 *Hz*, 1H, Ar-H quinazoline), 8.35 (d, *J* = 8.2 *Hz*, 1H, Ar-H quinazoline), 8.58 (s, 1H, NH). ^13^C NMR (DMSO-d_6_, 100 MHz) *δ* (ppm): 14.12, 19.95, 32.25, 114.81, 119.28, 122.34, 124.40, 126.97, 128.92, 136.27, 139.05, 146.02, 152.48, 155.09, 155.90, 168.65. Anal. Calcd. for C_19_H_19_ClN_4_O_2_ (370.84): C, 61.54; H, 5.16; N, 15.11. Found: C, 61.82; H, 4.97; N, 14.98%.

##### 2.1.1.3. N-(4-((2-Chloroquinazolin-4-yl)oxy)phenyl)benzamide (15_a_)

Grey solid: (yield: 0.19 g, 83.30%); m.p. = 244–245 °C; IR (KBr, cm^–1^): 3344 (NH), 3074, 3051 (C-H aromatic), 1654 (CO amide), 1612 (C = N), 1558, 1485, 1446 (C = C aromatic), 1531 (amide II band), 1203 (C–O); ^1^H NMR (DMSO-d_6_, 400 MHz) *δ* (ppm): 7.38 (d, *J* = 8.9 *Hz*, 2H, Ar-H), 7.55–7.65 (m, 3H, Ar–H), 7.82 (ddd, *J* = 8.2, 6.9, 1.2 *Hz*, 1H, Ar-H quinazoline), 7.91 − 8.00 (m, 5H, Ar-H), 8.10 (ddd, *J* = 8.5, 7.0, 1.5 *Hz*, 1H, Ar-H quinazoline), 8.41 (dd, *J* = 8.3, 1.5 *Hz*, 1H, Ar-H quinazoline), 10.42 (s, 1H, NH). ^13 ^C NMR (DMSO-d_6_, 100 MHz) *δ* (ppm): 114.87, 122.05, 122.42, 124.48, 127.13, 128.14, 128.91, 132.13, 135.32, 136.31, 137.73, 147.89, 152.63, 155.12, 166.14, 168.46. Anal. Calcd. for C_21_H_14_ClN_3_O_2_ (375.81): C, 67.12; H, 3.76; N, 11.18. Found: C, 66.86; H, 3.94; N, 11.40%.

##### 2.1.1.4. 4-Chloro-N-(4-((2-chloroquinazolin-4-yl)oxy)phenyl)benzamide (15_b_)

Brownish solid: (yield: 0.21 g, 86.70%); m.p. = 249–250 °C; IR (KBr, cm^–1^): 3336 (NH), 3059, 3043 (C-H aromatic), 1651 (CO amide), 1616 (C = N), 1562, 1489, 1446 (C = C aromatic), 1531 (amide II band), 1207 (C-O); ^1^H NMR (DMSO-d_6_, 400 MHz) *δ* (ppm): 7.35 (d, *J* = 8.4 *Hz*, 2H, Ar-H), 7.64 (d, *J* = 8.4 *Hz*, 2H, Ar-H), 7.82 (dd, *J* = 7.6 *Hz*, 1H, Ar-H quinazoline), 7.87 (d, *J* = 8.4 *Hz*, 2H, Ar-H), 7.95 (d, *J* = 8.4 *Hz*, 1H, Ar-H quinazoline), 8.02 (d, *J* = 8.4 *Hz*, 2H, Ar-H), 8.10 (ddd, *J* = 8.5, 7.0, 1.5 *Hz*, 1H, Ar-H quinazoline), 8.40 (dd, *J* = 8.4, 1.4 *Hz*, 1H, Ar-H quinazoline), 10.48 (s, 1H, NH). ^13 ^C NMR (DMSO-d_6_, 100 MHz) *δ* (ppm): 114.79, 122.32, 122.49, 124.47, 127.03, 128.91, 129.03, 130.05, 133.84, 136.42, 137.05, 137.31, 148.08, 152.54, 155.08, 165.30, 168.45. Anal. Calcd. for C_21_H_13_Cl_2_N_3_O_2_ (410.25): C, 61.48; H, 3.19; N, 10.24. Found: C, 61.69; H, 3.25; N, 9.95%.

##### 2.1.1.5. 3-Chloro-N-(4-((2-chloroquinazolin-4-yl)oxy)phenyl)benzamide (15_c_)

Brownish solid: (yield: 0.20 g, 80.50%); m.p. = 229–230 °C; IR (KBr, cm^–1^): 3290, 3128 (NH), 3055 (C-H aromatic), 1654 (CO amide), 1616 (C = N), 1566, 1489, 1450 (C = C aromatic), 1531 (amide II band), 1211 (C-O); ^1^H NMR (DMSO-d_6_, 400 MHz) *δ* (ppm): 7.40 (d, *J* = 8.92 *Hz*, 2H, Ar-H), 7.61 (dd, *J* = 7.8, 7.9 *Hz*, 1H, Ar-H), 7.69 (dd, *J* = 8.0, 1.9 *Hz*, 1H, Ar-H), 7.83 (ddd, *J* = 8.2, 7.0, 1.1 *Hz*, 1H, Ar-H quinazoline), 7.90–7.98 (m, 4H, Ar-H), 8.04 (t, *J* = 1.9 *Hz*, 1H, Ar-H), 8.11 (ddd, *J* = 8.5, 6.9, 1.5 *Hz*, 1H, Ar-H quinazoline), 8.42 (dd, *J* = 8.3, 1.5 *Hz*, 1H, Ar-H quinazoline), 10.52 (s, 1H, NH). ^13^C NMR (DMSO-d_6_, 100 MHz) *δ* (ppm): 114.88, 122.14, 122.49, 124.48, 126.98, 127.14, 127.90, 128.92, 130.94, 131.96, 133.73, 136.32, 137.28, 137.44, 148.08, 152.65, 155.11, 164.63, 168.45. Anal. Calcd. for C_21_H_13_Cl_2_N_3_O_2_ (410.25): C, 61.48; H, 3.19; N, 10.24. Found: C, 61.52; H, 3.41; N, 10.17%.

##### 2.1.1.6. N-(4-((2-Chloroquinazolin-4-yl)oxy)phenyl)nicotinamide (15_d_)

Grey solid: (yield: 0.16 g, 71.50%); m.p. = 202–203 °C; IR (KBr, cm^–1^): 3336, 3120 (NH), 3062, 3039 (C–H aromatic), 1651 (CO amide), 1612 (C = N), 1580, 1562, 1489, 1450 (C = C aromatic), 1531 (amide II band), 1211 (C–O); ^1^H NMR (DMSO-d_6_, 400 MHz) *δ* (ppm): 7.40 (d, *J* = 8.9 *Hz*, 2H, Ar-H), 7.60 (dd, *J* = 7.8, 4.8 *Hz*, 1H, Ar-H pyridine), 7.82 (dd, *J* = 7.6, *Hz*, 1H, Ar-H quinazoline), 7.90–7.97 (m, 3H, Ar-H), 8.10 (ddd, *J* = 7.7, 7, 1.3 *Hz*, 1H, Ar-H quinazoline), 8.32 (ddd, *J* = 7.9, 1.8, 1.8 *Hz*, 1H, Ar-H pyridine), 8.42 (d, *J* = 7.6 *Hz*, 1H, Ar-H quinazoline ), 8.79 (dd, *J* = 4.8, 1.5 *Hz*, 1H, Ar-H pyridine), 9.14 (d, *J* = 1.7 *Hz* 1H, Ar-H pyridine), 10.61 (s, 1H, NH). ^13 ^C NMR (DMSO-d_6_, 100 MHz) *δ* (ppm): 114.83, 122.10, 122.52, 124.02, 124.44, 127.11, 128.88, 130.96, 135.96, 136.28, 137.37, 148.11, 149.13, 152.61, 152.64, 155.09, 164.62, 168.41. Anal. Calcd. for C_20_H_13_ClN_4_O_2_ (376.80): C, 63.75; H, 3.48; N, 14.87. Found: C, 63.51; H, 3.64; N, 14.93%.

#### 2.1.2. General method for synthesis of compounds (19_a&b_) and (20_a–d_)

Equimolar amounts of 2,3-dichloroquinoxaline (0.12 g, 0.60 mmol) and the appropriate intermediate (**8_a&b_** and **10_a–d_**) along with K_2_CO_3_ (0.10 g, 0.72 mmol) as a base were mixed in isopropanol. The reaction mixture was refluxed for approximately 5 h then allowed to cool. The obtained precipitate was collected by filtration, washed by water, dried, and recrystallized from isopropanol.

##### 2.1.2.1. 1-(4-((3-Chloroquinoxalin-2-yl)oxy)phenyl)-3-phenylurea (19_a_)

White solid: (yield: 0.18 g, 78.65%); m.p. = 248–249 °C; IR (KBr, cm^–1^): 3305, 3282, 3201, 3143 (NH), 3066, 3039 (C–H aromatic), 1647 (CO urea), 1600 (C = N), 1554, 1504, 1446 (C = C aromatic), 1222, 1195 (C–O); ^1^H NMR (DMSO-d_6_, 400 MHz) *δ* (ppm): 6.99 (t, *J* = 7.4 *Hz*, 1H, Ar-H), 7.30 (m, 4H, Ar-H), 7.48 (d, *J* = 7.6 *Hz*, 2H, Ar-H), 7.56 (d, *J* = 8.9 *Hz*, 2H, Ar-H), 7.75 (m, 3H, Ar-H quinoxaline), 8.03 (dd, *J* = 8.3, 1.6 *Hz*, 1H, Ar-H quinoxaline), 8.82 (s, 1H, NH), 8.90 (s, 1H, NH). ^13 ^C NMR (DMSO-d_6_, 100 MHz) *δ* (ppm): 118.91, 120.17, 122.44, 122.61, 127.19, 128.02, 129.03, 129.31, 131.48, 137.73, 138.86, 139.02, 139.26, 139.88, 147.22, 153.23, 153.35. Anal. Calcd. for C_21_H_15_ClN_4_O_2_ (390.83): C, 64.54; H, 3.87; N, 14.34. Found: C, 64.30; H, 3.95; N, 14.59%.

##### 2.1.2.2. 1-Butyl-3-(4-((3-chloroquinoxalin-2-yl)oxy)phenyl)urea (19_b_)

White solid: (yield: 0.16 g, 70.90%); m.*p* = 227–228 °C; IR (KBr, cm^–1^): 3317, 3116 (NH), 3059, 3043 (C–H aromatic), 2958, 2931, 2870 (C–H aliphatic), 1639 (CO urea), 1604 (C = N), 1562, 1504, 1462 (C = C aromatic), 1222, 1195 (C–O); ^1^H NMR (DMSO-d_6_, 400 MHz) *δ* (ppm): 0.92 (t, *J* = 7.2 *Hz*, 3H, CH_3_), 1.32 (m, 2H, *CH_2_*CH_3_), 1.42 (m, 2H, *CH_2_*CH_2_CH_3_), 3.10 (td, *J* = 6.6, *Hz*, 2H), 6.16 (t, *J* = 5.6 *Hz*, 1H, D_2_O exchangeable, *NH*CH_2_), 7.20 (d, *J* = 8.8 *Hz*, 2H, Ar-H), 7.47 (d, *J* = 8.8 *Hz*, 2H, Ar-H), 7.74 (m, 3H, Ar-H quinoxaline), 8.02 (dd, *J* = 7.7, 1.7 *Hz*, 1H, Ar-H quinoxaline), 8.51 (s, 1H, D_2_O exchangeable, NH). ^13 ^C NMR (DMSO-d_6_, 100 MHz) *δ* (ppm): 14.17, 19.99, 32.36, 39.35, 119.16, 122.26, 127.25, 128.09, 128.87, 131.34, 138.84, 138.92, 139.16, 139.33, 146.49, 153.48, 155.75. Anal. Calcd. for C_19_H_19_ClN_4_O_2_ (370.84): C, 61.54; H, 5.16; N, 15.11. Found: C, 61.32; H, 5.37; N, 15.35%.

##### 2.1.2.3. N-(4-((3-Chloroquinoxalin-2-yl)oxy)phenyl)benzamide (20_a_)

Brownish solid: (yield: 0.19 g, 82.70%); m.p. = 258–259 °C; IR (KBr, cm^–1^): 3344 (NH), 3059, 3028 (C–H aromatic), 1647 (CO amide), 1608 (C = N), 1562, 1508, 1446 (C = C aromatic), 1531 (amide II band), 1226, 1192 (C–O); ^1^H NMR (DMSO-d_6_, 400 MHz) *δ* (ppm): 7.36 (d, *J* = 8.9 *Hz*, 2H, Ar-H), 7.57 (m, 2H, Ar-H), 7.61 (m, 1H, Ar-H), 7.76 (m, 3H, Ar-H quinoxaline), 7.90 (d, *J* = 8.9 *Hz*, 2H, Ar-H), 8.01 (m, 3H, Ar-H), 10.40 (s, 1H, NH). ^13 ^C NMR (DMSO-d_6_, 100 MHz) *δ* (ppm): 122.16, 122.31, 127.32, 128.12, 128.14, 128.91, 129.00, 131.40, 132.12, 135.34, 137.32, 139.02, 139.12, 139.31, 148.52, 153.37, 166.11. Anal. Calcd. for C_21_H_14_ClN_3_O_2_ (375.81): C, 67.12; H, 3.76; N, 11.18. Found: C, 66.87; H, 3.95; N, 10.94%.

##### 2.1.2.4. 4-Chloro-N-(4-((3-chloroquinoxalin-2-yl)oxy)phenyl)benzamide (20_b_)

Grey solid: (yield: 0.21 g, 83.60%); m.p. = 264 − 265 °C; IR (KBr, cm^-1^): 3259, 3143 (NH), 3070, 3035 (C–H aromatic), 1647 (CO amide), 1608 (C = N), 1597, 1504, 1462 (C = C aromatic), 1531 (amide II band), 1222, 1195 (C–O); ^1^H NMR (DMSO-d_6_, 400 MHz) *δ* (ppm): 7.36 (d, *J* = 8.9 *Hz*, 2H, Ar-H), 7.63 (d, *J* = 8.5 *Hz*, 2H, Ar-H), 7.75 (m, 3H, Ar-H quinoxaline), 7.88 (d, *J* = 8.9 *Hz*, 2H, Ar-H), 8.02 (m, 3H, Ar-H), 10.49 (s, 1H, NH). ^13 ^C NMR (DMSO-d_6_, 100 MHz) *δ* (ppm): 122.23, 122.32, 127.31, 128.11, 128.96, 128.98, 130.13, 131.38, 134.01, 136.94, 137.16, 139.01, 139.11, 139.30, 148.62, 153.34, 164.96. Anal. Calcd. for C_21_H_13_Cl_2_N_3_O_2_ (410.25): C, 61.48; H, 3.19; N, 10.24. Found: C, 61.21; H, 2.90; N, 10.58%.

##### 2.1.2.5. 3-Chloro-N-(4-((3-chloroquinoxalin-2-yl)oxy)phenyl)benzamide (20_c_)

Grey solid: (yield: 0.20 g, 79.85%); m.p. = 243–244 °C; IR (KBr, cm^–1^): 3290, 3136 (NH), 3066 (C–H aromatic), 1651 (CO amide), 1608 (C = N), 1562, 1512, 1473 (C = C aromatic), 1531 (amide II band), 1222, 1199 (C–O); ^1^H NMR (DMSO-d_6_, 400 MHz) *δ* (ppm): 7.37 (d, *J* = 8.9 *Hz*, 2H, Ar-H), 7.61 (m, 1H, Ar-H), 7.69 (m, 1H, Ar-H) 7.76 (m, 3H, Ar-H quinoxaline), 7.88 (d, *J* = 8.9 *Hz*, 2H, Ar-H), 7.95 (d, *J* = 7.6 *Hz*, 1H, Ar-H), 8.02 (m, 2H, Ar-H), 10.49 (s, 1H, NH). ^13 ^C NMR (DMSO-d_6_, 100 MHz) *δ* (ppm): 122.23, 122.36, 126.98, 127.32, 127.89, 128.11, 129.00, 130.93, 131.39, 131.94, 133.72, 137.03, 137.30, 139.02, 139.11, 139.30, 148.70, 153.33, 164.59. Anal. Calcd. for C_21_H_13_Cl_2_N_3_O_2_ (410.25): C, 61.48; H, 3.19; N, 10.24. Found: C, 61.29; H, 2.98; N, 10.54%.

##### 2.1.2.6. N-(4-((3-Chloroquinoxalin-2-yl)oxy)phenyl)nicotinamide (20_d_)

White solid: (yield: 0.17 g, 74.45%); m.p. = 219–220 °C; IR (KBr, cm^–1^): 3329 (NH), 3066, 3047 (C–H aromatic), 1651 (CO amide), 1612 (C = N), 1562, 1508 (C = C aromatic), 1523 (amide II band), 1222, 1192 (C–O); ^1^H NMR (DMSO-d_6_, 400 MHz) *δ* (ppm): 7.38 (d, *J* = 8.8 *Hz*, 2H, Ar-H), 7.60 (dd, *J* = 7.7, 4.8 *Hz* 1H, Ar-H pyridine), 7.76 (m, 3H, Ar-H quinoxaline), 7.88 (d, *J* = 8.8 *Hz*, 2H, Ar-H), 8.02 (d, *J* = 8.0 *Hz*, 1H, Ar-H quinoxaline), 8.32 (d, *J* = 7.9 *Hz*, 1H, Ar-H pyridine), 8.79 (d, *J* = 4.3 *Hz*, 1H, Ar-H pyridine), 9.15 (s, 1H, Ar-H pyridine). 10.58 (s, 1H, D_2_O exchangeable, NH). ^13 ^C NMR (DMSO-d_6_, 100 MHz) *δ* (ppm): 122.20, 122.43, 124.06, 127.32, 128.11, 129.04, 131.00, 131.43, 135.98, 136.96, 139.02, 139.10, 139.30, 148.74, 149.11, 152.65, 153.34, 164.62. Anal. Calcd. for C_20_H_13_ClN_4_O_2_ (376.80): C, 63.75; H, 3.48; N, 14.87. Found: C, 63.61; H, 3.44; N, 15.11%.

#### 2.1.3. *General method for synthesis of compounds* (22_a&b_) and (23_a–d_)

Equimolar amounts of 2-fluronitrobenzen (0.10 ml, 0.71 mmol) and the appropriate intermediate (**8_a&b_** and **10_a–c_**) were mixed with K_2_CO_3_ (0.12 g, 0.86 mmol) in isopropanol. The reaction mixture was heated under reflux for about 3 h then cooled. The obtained precipitate was filtered, washed by water several times then dried and recrystallized from isopropanol.

##### 2.1.3.1. 1-(4-(2-Nitrophenoxy)phenyl)-3-phenylurea (22_a_)

Grey solid: (yield: 0.21 g, 84.20%); m.p. = 175–176 °C; IR (KBr, cm^–1^): 3302, 3190, 3136 (NH), 3093, 3062, 3035 (C–H aromatic), 1643 (CO urea) 1597, 1558, 1504, 1473 (C = C aromatic), 1523, 1342 (NO_2_), 1234 (C–O); ^1^H NMR (DMSO-d_6_, 400 MHz) *δ* (ppm): 6.98 (t, *J* = 7.3 *Hz*, 1H, Ar-H ), 7.06 (m, 3H, Ar-H), 7.29 (m, 3H, Ar-H), 7.46 (d, *J* = 7.8 *Hz*, 2H, Ar-H), 7.54 (d, *J* = 8.9 *Hz*, 2H, Ar-H), 7.66 (ddd, *J* = 7.9, 1.4 *Hz*, 1H, Ar-H), 8.03 (dd, *J* = 8.1, 1.3 *Hz*, 1H, Ar-H), 8.67 (s, 1H, NH), 8.75 (s, 1H, NH). ^13 ^C NMR (DMSO-d_6_, 100 MHz) *δ* (ppm): 118.79, 120.07, 120.19, 120.53, 122.41, 123.65, 125.95, 129.25, 135.30, 137.01, 140.03, 141.10, 150.08, 150.66, 153.10. Anal. Calcd. for C_19_H_15_N_3_O_4_ (349.35): C, 65.32; H, 4.33; N, 12.03. Found: C, 65.71; H, 4.50; N, 11.98%.

##### 2.1.3.2. 1-Butyl-3-(4-(2-nitrophenoxy)phenyl)urea (22_b_)

Grey solid: (yield: 0.18 g, 77.50%); m.p. = 163–164 °C; IR (KBr, cm^–1^): 3317, 3170, 3113 (NH), 3074, 3043 (C–H aromatic), 2958, 2931, 2862 (C–H aliphatic), 1635 (CO urea), 1600, 1585, 1504, 1473 (C = C aromatic), 1523, 1342 (NO_2_), 1246 (C–O); ^1^H NMR (DMSO-d_6_, 400 MHz) *δ* (ppm): 0.90 (t, *J* = 7.2 Hz, 3H, CH_3_), 1.29 (m, 2H, *CH_2_*CH_3_), 1.42 (m, 2H, *CH_2_*CH_2_CH_3_), 3.08 (dt, *J* = 6.5 Hz, 2H, *CH_2_*NH), 6.11 (t, *J* = 5.7 Hz, 1H, *NH*CH_2_), 7.00 (m, 3H, Ar-H), 7.29 (ddd, *J* = 8.1, 7.4, 1.2 *Hz*, 1H, Ar-H), 7.46 (d, *J* = 8.9 *Hz*, 2H, Ar-H), 7.64 (ddd, *J* = 8.7, 7.4, 1.5 *Hz*, 1H, Ar-H), 8.03 (dd, *J* = 8.1, 1.4 *Hz*, 1H, Ar-H), 8.48 (s, 1H, NH). ^13 ^C NMR (DMSO-d_6_, 100 MHz) *δ* (ppm): 14.15, 19.97, 32.34, 39.18, 119.67, 119.72, 120.29, 123.60, 125.92, 135.42, 138.16, 141.00, 149.13, 150.93, 155.69. Anal. Calcd. for C_17_H_19_N_3_O_4_ (329.36): C, 62.00; H, 5.81; N, 12.76. Found: C, 61.87; H, 5.97; N, 12.59%.

##### 2.1.3.3. N-(4–(2-Nitrophenoxy)phenyl)benzamide (23_a_)

Brownish solid: (yield: 0.21 g, 88.25%); m.p. = 197–198 °C; IR (KBr, cm^–1^): 3336 (NH), 3055 (C–H aromatic), 1651 (CO amide), 1600, 1585, 1508, 1477 (C = C aromatic), 1535 (amide II band), 1535, 1350 (NO_2_), 1234 (C–O); ^1^H NMR (DMSO-d_6_, 400 MHz) *δ* (ppm): 7.11 (m, 3H, Ar-H ), 7.35 (ddd, *J* = 8.3, 7.6, 1.0 *Hz*, 1H, Ar-H), 7.57 (m, 2H, Ar-H), 7.61 (m, 1H, Ar-H), 7.67 (ddd, *J* = 8.8, 7.6, 1.6 *Hz*, 1H, Ar-H), 7.84 (d, *J* = 9.0 *Hz*, 2H, Ar-H), 7.96 (m, 2H, Ar-H), 8.07 (dd, *J* = 8.2, 1.6 *Hz*, 1H, Ar-H), 10.35 (s, 1H, NH). ^13 ^C NMR (DMSO-d_6_, 100 MHz) *δ* (ppm): 119.58, 120.71, 122.82, 124.36, 126.03, 128.03, 128.95, 132.18, 135.09, 13549, 136.09, 141.32, 150.14, 151.71, 166.20. Anal. Calcd. for C_19_H_14_N_2_O_4_ (334.33): C, 68.26; H, 4.22; N, 8.38. Found: C, 67.98; H, 4.43; N, 8.25%.

##### 2.1.3.4. 3-Chloro-N-(4–(2-nitrophenoxy)phenyl)benzamide (23_b_)

Brownish solid: (yield: 0.21 g, 81.60%); m.p. = 169–170 °C; IR (KBr, cm^–1^): 3313 (NH), 3074, 3047 (C–H aromatic), 1651 (CO amide), 1600, 1585, 1508, 1473 (C = C aromatic), 1527 (amide II band), 1527, 1350 (NO_2_), 1242 (C–O); ^1^H NMR (DMSO-d_6_, 400 MHz) *δ* (ppm): 7.12 (m, 3H, Ar-H ), 7.35 (dd, *J* = 7.5 *Hz*, 1H, Ar-H), 7.59 (dd, *J* = 7.9 *Hz*, 1H, Ar-H), 7.69 (m, 2H, Ar-H), 7.83 (d, *J* = 9.0 *Hz*, 2H, Ar-H), 7.92 (d, *J* = 7.7 *Hz*, 1H, Ar-H), 8.02 (s, 1H, Ar-H), 8.06 (dd, *J* = 8.1, 1.3 *Hz*, 1H, Ar-H), 10.44 (s, 1H, NH). ^13 ^C NMR (DMSO-d_6_, 100 MHz) *δ* (ppm): 119.68, 120.70, 122.71, 124.30, 126.05, 126.93, 127.85, 130.90, 131.91, 133.71, 135.44, 136.01, 137.23, 141.37, 150.19, 151.79, 164.48. Anal. Calcd. for C_19_H_13_ClN_2_O_4_ (368.77): C, 61.88; H, 3.55; N, 7.60. Found: C, 61.63; H, 3.71; N, 7.84%.

##### 2.1.3.5. N-(4–(2-Nitrophenoxy)phenyl)nicotinamide (23C)

Grey solid: (yield: 0.18 g, 74.90%); m.p.= 160–161 °C; IR (KBr, cm^–1^): 3336 (NH), 3032 (C–H aromatic), 1647 (CO amide), 1600, 1585, 1508, 1477 (C = C aromatic), 1527 (amide II band), 1527, 1346 (NO_2_), 1230 (C–O); ^1^H NMR (DMSO-d_6_, 400 MHz) *δ* (ppm): 7.13 (m, 3H, Ar-H), 7.35 (ddd, *J* = 8.2, 7.6, 1.0 *Hz*, 1H, Ar-H), 7.59 (ddd, *J* = 7.9, 4.9, 0.6 *Hz*, 1H, Ar-H pyridine), 7.69 (ddd, *J* = 8.9, 7.3, 1.6 *Hz*, 1H, Ar-H), 7.83 (d, *J* = 9.0 *Hz*, 2H, Ar-H), 8.06 (dd, *J* = 8.1, 1.5 *Hz*, 1H, Ar-H), 8.30 (ddd, *J* = 7.9, 2.4, 1.5 *Hz*, 1H, Ar-H pyridine), 8.77 (dd, *J* = 4.7, 1.5 *Hz*, 1H, Ar-H pyridine), 9.12 (dd, *J* = 2.4, 0.8 *Hz,* 1H, Ar-H pyridine),10.53 (s, 1H, NH). ^13 ^C NMR (DMSO-d_6_, 100 MHz) *δ* (ppm): 119.72, 120.71, 122.66, 124.01, 124.32, 126.06, 130.95, 135.45, 135.93, 135.97, 141.37, 149.09, 150.18, 151.83, 152.61, 164.48. Anal. Calcd. for C_18_H_13_N_3_O_4_ (335.32): C, 64.48; H, 3.91; N, 12.53. Found: C, 64.29; H, 3.84; N, 12.27%.

### 2.2. Biological testing

#### 2.2.1. VEGFR-2 inhibition testing

Enzyme linked immunosorbent assay (ELISA) technique was applied to human VEGFR-2 ELISA kit to calculate IC_50_ of all new derivatives against VEGFR-2 kinase. The antibody specific to the enzyme was planted on a 96 well microplate to which different micromolar concentrations of the positive control and the new compounds was added. After incubation at 25 °C for about 2.5 h, the biotin antibody (100 µL) was added. The plates were further incubated for 1 h at 25 °C and then washed. A solution of streptavidin (100 µL) was added to the microplates which were incubated for another 45 min at 25 °C and then washed. Similarly, a solution of tetramethybenzidine (TMB) substrate (100 µL) was added with incubation at 25 °C for half an hour. Finally, stop solution (50 µL) was added and the reading was immediately determined at 450 nm. To get the standard curve, the concentrations used were presented on the X-axis and the corresponding absorbances were on the Y-axis.

#### 2.2.2. *In vitro* antitumor testing

The antitumor assay was performed against the human cancer cell lines: HepG2, PC3, and MCF-7. The chemicals used in this test were supplied by Sigma-Aldrich with high analytical grade. National Cancer Institute (Cairo, Egypt) was the supplier of the cell lines tested which originally obtained from American Type Culture Collection (ATCC). MTT protocol was applied to quantitatively measure the antiproliferative activity of the new derivatives^34^. In this test, the colour change is attributed to that the yellow tetrazolium bromide (MTT) is converted to formazan derivative which has purple colour. This biochemical reaction occurs only in viable cells by mitochondrial succinate dehydrogenase. The tested cells were initially cultured in RPMI-1640 medium with foetal bovine serum (10%). To the incubated cell, streptomycin (100 µg/mL) and penicillin (100 units/mL) were added in a 5% CO_2_ incubator at 37 °C. Then seeding of the cells was performed in a 96-well plate under 5% CO_2_ and at density of 1.0 × 10^4^ cells/well at 37 °C for 48 h. Various non-toxic concentrations of the tested candidates were added and the cells were then incubated for further 24 h to the cells treated with the new compounds, MTT solution (20 µL at 5 mg/mL) was added, followed by 4 h incubation. One hundred microlitres of Dimethyl sulfoxide (DMSO) were added into each well to dissolve the purple formazan formed which is colorimetrically absorbed at 570 nm using a plate reader (EXL 800, USA). Finally, the percentages of cell viability was obtained as (A570 of treated samples/A570 of untreated sample) × 100.

#### 2.2.3. Effect of 15_d_ on cell cycle of HepG2

The protocol reported by Wang et al.^35^ was followed to determine the effect of 15_d_ on cell cycle of HepG2. The cancer cells (2 × 10^5^ cells) were seeded and incubated in each of six-well plates for 24 h. The cells were further incubated at 37 °C and 5% CO_2_ after addition of foetal bovine serum (FBS, 10%). The medium was replaced by DMSO solution (1% *v/v*) in which 9.6 µg of compound **15_d_** was dissolved. Well plates were then incubated for 48 h and then rinsed with cold phosphate-buffered saline (PBS), fixed with ethanol (70%) and washed again with PBS. DNA fluorochrome propidium iodide (PI) was used to stain the cells which were then kept for 15 min at 37 °C. FACS Calibre flow cytometer was used to analyse the samples.

#### 2.2.4. Effect of 15_d_ on apoptosis rate of HepG2

Apoptosis ratio of HepG2 treated with 9.6 µg of compound **15_d_** in comparison with untreated HepG2 cells was calculated according to the reported method using Annexin V fluorescein isothiocyanate (V-FITC)/PI kit^36^.

#### 2.2.5. Effect of 15_d_ on apoptotic markers

HepG2 cells were cultured and treated with compound **15_d_** for 24 h. after that cells were collected and washed with PBS, then were collected and lysed by adding it to the extraction buffer containing protease inhibitors (1 ml per 1 × 10^6^ cells.) then the lysate was diluted immediately before the assay. Caspase-3, BAX, Bcl-2, and p53 level was measured by using Quantikine-Human active kits (R&D Systems, Inc. Minneapolis, USA) according to the manufacturer protocols.

#### 2.2.6. Effect of 15d on multi types of kinases

Inhibitory activity of compound **15_d_** against HER2 and FGFR was performed using Homogeneous time resolved fluorescence (HTRF) assay method. Different kinases and ATP were obtained from Sigma. The specified kinase and its substrate was incubated with compound for 5 min in buffer solution to start the enzymatic reaction; ATP was then added to the reaction mixture and was maintained for 30 min at room temperature. The reaction was stopped by adding detection reagents containing EDTA for 1 h and then the IC_50_ values were determined by GraphPad Prism 5.0.

### 2.3. Molecular modelling

Molecular docking studies for the new molecules were performed by using *Discovery Studio 2.5* software (Accelrys software Inc., USA) against VEGFR-2. The 3D structure of the VEGFR-2 kinase combined with sorafenib was downloaded from Protein Data Bank (PDB) website (PDB ID: 4ASD^37^).

To prepare the obtained structure, water molecules were deleted. Then correction of the valence of atoms was achieved by running the valence monitor method. To minimise the energy, force fields CHARMM and MMFF94 were applied^38–40^. The active site was then defined and prepared for docking. The new derivatives were sketched using *ChemBioDraw Ultra 14.0* software ^41^ and saved as MDL-SD files. The co-crystallized ligand was similarly drawn. The drawn structures were opened and prepared for docking by 3 D protonation, and energy was minimised through running force fields CHARMM and MMFF94. Protocol selected for docking process was CDOCKER. Meanwhile, CHARMM-based molecular dynamics (MD) was used to dock the co-crystallized ligand^42–44^. Ten docked poses were obtained for each molecule. Finally, the conformers of the most minimal free energy were selected to study the binding pattern of each molecule.

## 3 Results and discussion

### 3.1. Chemistry

**Scheme 1.**
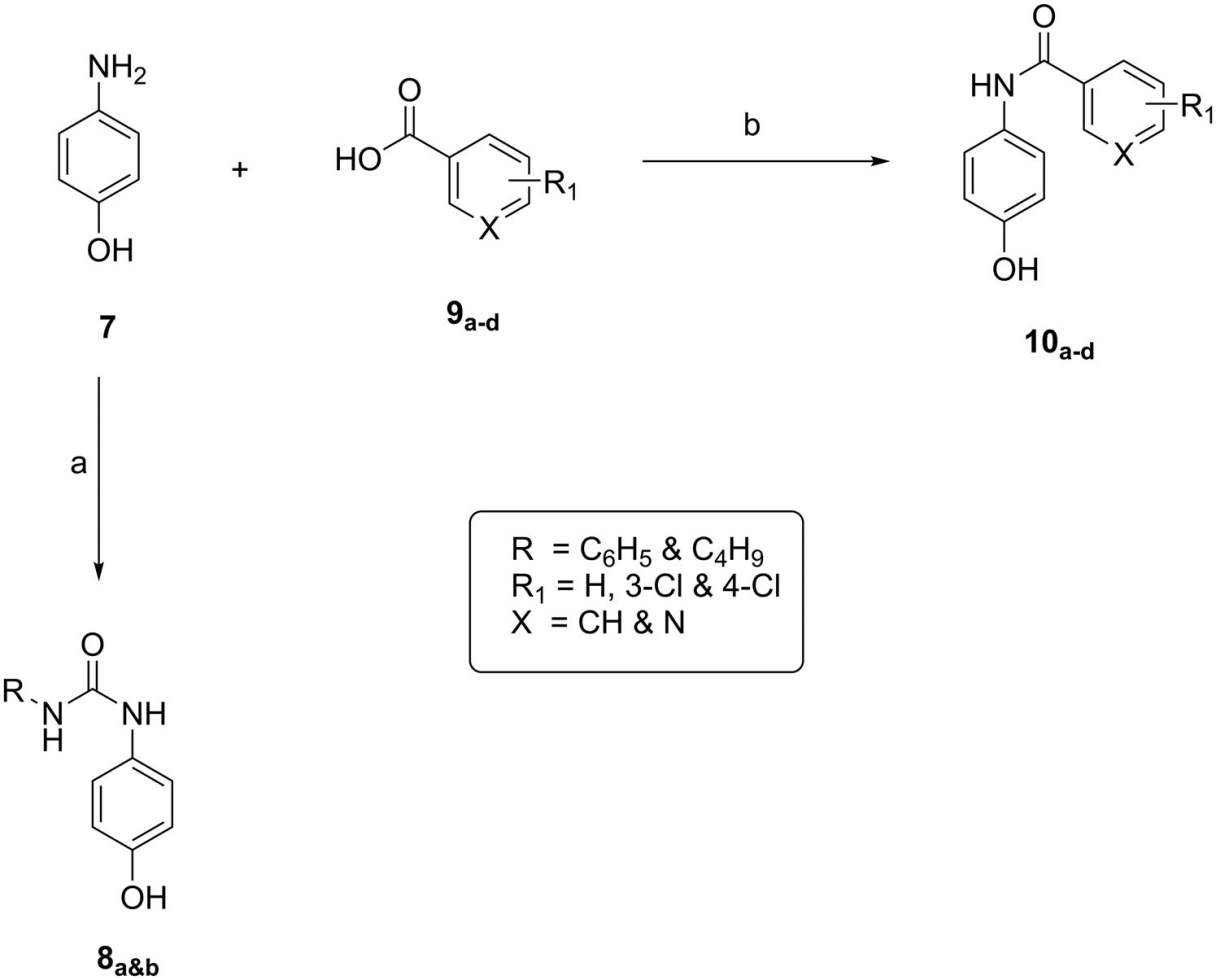
General procedure for preparation of intermediates **9a,b** and **10a–d**; Reagents and conditions: (a) phenyl or butylisocyanate, absolute ethanol, reflux, 2 h. (b) DCM, ClCOOEt, Et3N, ice salt bath.

Firstly, preparation of the intermediates **8_a&b_** and **10_a-d_** was achieved as illustrated in Scheme 1. Heating of 4-aminophenol with phenylisocyanate and butylisocyanate in absolute ethanol afforded **8_a&b_**, respectively,^45^ while the intermediates **10_a–d_**^46–48^ were obtained by reaction of 4-aminophenol with benzoic acid derivatives namely, benzoic acid, 4-chlorobenzoic acid and 3-chlorobenzoic acid and with nicotinic acid, respectively. Synthesis of these intermediates was afforded by treating an appropriate acid with ethyl chloroformate and triethyl amine in DCM at about –5.0 °C for 1 h. Then 4-aminophenol was added to the reaction mixture which stirred at r.t. for 1 h.

**Scheme 2.**
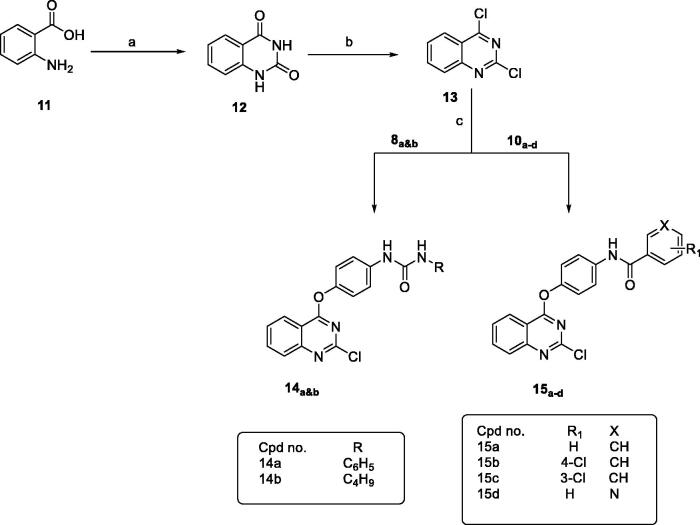
General procedure for preparation of final compounds **14a&b** and **15a–d**; Reagents and conditions: (a) urea, fusion, 4 h. (b) POCl_3_, Et_3_N, reflux, 6 h. (c) K_2_CO_3_, isopropanol, reflux, 1 h.

**Scheme 3.**
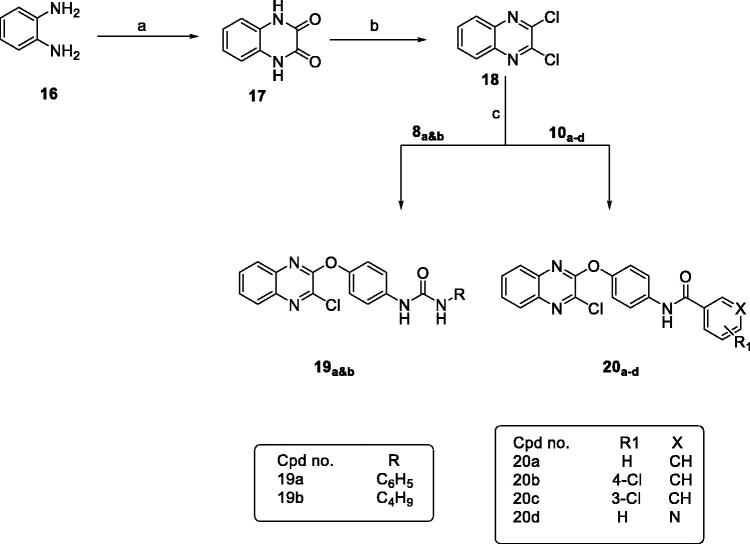
General procedure for synthesis of final compounds **19a&b** and **20a–d**; Reagents and conditions: (a) oxalic acid, HCl, H_2_O, reflux. (b) POCl_3_, DMF, reflux, 3 h. (c) K_2_CO_3_, isopropanol, reflux, 3 h.

Secondly, synthesis of dichloroquinazoline (**13**) and dichloroquinoxaline (**18**) according to the reported procedures provided in Schemes 2 and 3. Compound **13** was obtained by fusion of anthranilic acid with urea to give quinazoline-2,4-dione which was then chlorinated by refluxing with POCl_3_ in presence of Et_3_N^49^, while reaction of oxalic acid with o-phenylenediamine in 4 N HCl afforded quinoxaline-2,3-dione which was then chlorinated with POCl_3_ in presence of DMF to give compound **18**^50^.

Similarly, reaction of the intermediates (**8_a&b_** and **10_a–d_**) with compound **13** or **18** in isopropanol and in presence of K_2_CO_3_ gave the final compounds **14_a&b_, 15_a–d_, 19_a&b_** and **20_a–d_**, respectively, as shown in Schemes 2 and 3.

**Scheme 4.**
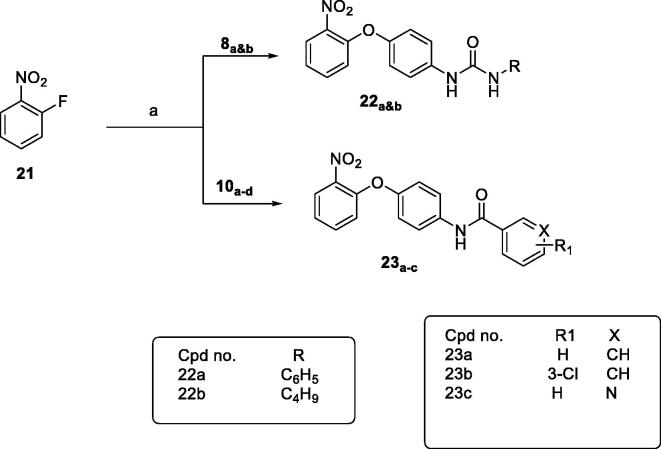
General procedure for synthesis of final compounds **22a&b** and **23a–d**; Reagents and conditions: (a) K_2_CO_3_, isopropanol, reflux, 2h.

Finally, treatment of 2-fluronitrobenzene with the appropriate intermediate in isopropanol gave the final compounds **22_a&b_** and **23_a–d_** as shown in Scheme 4.

In ^1^H NMR charts of amide-containing compounds, the peak of amidic NH appeared at about 10.4 ppm. Compounds **14_a_**, **19_a_**, and **22_a_** showed peaks of the two NH of urea at about 8.7 and 8.8 ppm. While aliphatic and aromatic NH of urea of compounds **14_b_, 19_b_**, and **22_b_** exhibited their peaks at 6.1 and 8.6 ppm, respectively, pyridine ring in compounds **15_d_**, **20_d_**, and **23_c_** demonstrated two peaks at about 9 ppm which is a characteristic chemical shift of its two ortho protons.

Moreover, amidic carbonyl revealed a peak at about 166 ppm in ^13^C NMR charts. While urea carbonyl group appeared at about 153 ppm, regarding quinazoline nucleus, it showed one peak at about 168 ppm and two peaks above 150 ppm which are due to 3 C = N. While quinoxaline nucleus demonstrated one peak above 150 ppm, it showed no peaks above 160 ppm.

### 3.2. Biological testing

The synthesised compounds were evaluated biologically according to the following strategy; initially, all compounds were screened for *in vitro* inhibition of VEGFR-2 kinase. Then, antiproliferative assay of the best nine compounds was performed against three cancer cell lines namely: hepatocellular carcinoma (HepG2), prostate cancer (PC3), and breast cancer (MCF-7) and against one normal cell line as well. Finally, HepG2 cells treated with compound **15_d_** were evaluated for apoptosis and cell cycle kinetics compared to untreated HepG2 cells.

#### 3.2.1. VEGFR-2 inhibition testing

The novel derivatives were investigated *in vitro* for the inhibitory activities against VEGFR-2 as a possible mechanism of its antitumor activity. In the meantime, sorafenib was used as a positive control. Half-maximal inhibitory concentration (IC_50_) values as presented in Table 1 were calculated from the obtained concentration–inhibition response curve. As can be seen from Table 1, the new derivatives revealed inhibitory activity of the enzyme with IC_50_ values ranging from 60.00 to 123.85 nM. Sorafenib, meanwhile, showed IC_50_ = 54.00 nM. Compounds **15_d_, 15_c_, 14_a_, 15_a_, 20_d_, 23_a_, 19_b_, 15_b_**, and **20_c_** were found to be the most effective candidates with potency ranging from 0.90 to 0.62 of the Sorafenib potency. Compound **15_d_** emerged as the most effective candidate with IC_50_ = 60.00 nM.

**Table 1.** IC_50_ obtained for all the new derivatives as well as sorafenib against VEGFR-2 kinase.

Serial	Comp. no.	VEGFR-2 kinase IC_50_ (nM)
1	**14_a_**	67.05 ± 1.07
2	**14_b_**	94.22 ± 1.53
3	**15_a_**	67.25 ± 1.54
4	**15_b_**	86.36 ± 1.76
5	**15_c_**	65.24 ± 1.36
6	**15_d_**	60.00 ± 1.45
7	**19_a_**	93.63 ± 1.82
8	**19_b_**	75.18 ± 1.57
9	**20_a_**	107.94 ± 2.25
10	**20_b_**	113.74 ± 2.83
11	**20_c_**	86.56 ± 1.75
12	**20_d_**	69.55 ± 1.16
13	**22_a_**	123.85 ± 3.78
14	**22_b_**	107.46 ± 2.00
15	**23_a_**	74.85 ± 1.67
16	**23_b_**	105.31 ± 2.50
17	**23_c_**	119.25 ± 3.16
18	Sorafenib	54.00 ± 1.40

#### 3.2.2. *In vitro* antitumor assay

The potential antitumor activity of the selected nine compounds **14_a_, 15_a_, 15_b_, 15_c_, 15_d_, 19_b_, 20_c_, 20_d_**, and **23_a_** was further investigated against HepG2, PC3, and MCF-7. As is presented in Table 2 as IC_50_ values of the new derivatives against the aforementioned cancer cell lines, the following results can be concluded:

**Table 2.** Anti-proliferative activities towards HePG2, PC3, MCF-7, and WI-38 for the newly selected derivatives.

S.	Comp. No.	IC_50_ (µM)^a^			
		HepG2	PC3	MCF-7	WI-38
1	14_a_	41.49 ± 3.6	50.67 ± 4.36	58.26 ± 4.87	234.72 ± 2.43
2	15_a_	34.59 ± 2.82	30.28 ± 2.56	47.10 ± 3.59	250.33 ± 2.51
3	15_b_	17.39 ± 1.54	25.58 ± 2.31	19.88 ± 1.79	233.21 ± 2.39
4	15_c_	50.41 ± 4.39	55.95 ± 1.9	62.68 ± 5.12	236.15 ± 2.39
5	15_d_	24.10 ± 2.12	40.90 ± 3.46	33.40 ± 2.92	221.25 ± 2.55
6	19_b_	73.38 ± 5.95	84.41 ± 6.68	105.48 ± 7.57	230.16 ± 2.51
7	20_c_	144.29 ± 9.51	157.00 ± 10.00	134.85 ± 9.02	402.88 ± 3.26
8	20_d_	221.38 ± 11.44	>300	246.20 ± 13.56	460.69 ± 4.04
9	23_a_	152.93 ± 10.77	221.31 ± 12.87	233.92 ± 13.17	758.20 ± 5.15
10	Doxorubicin	14.61 ± 1.10	16.32 ± 1.10	12.41 ± 0.74	NT
11	Sorafenib	12.90 ± 0.85	19.38 ± 1.34	14.49 ± 1.68	NT

^a^IC_50_ values are the mean ± S.D. of three separate experiments.

NT: not tested.

1. HepG2: The descending order of the tested derivatives was as follows: **15 _b_** (IC_50_ = 17.39 µM) > **15_d_** (IC_50_ = 24.10 µM) > **15_a_** (IC_50_ = 34.59 µM) > **14_a_** (IC_50_ = 41.49 µM) > **15_c_** (IC_50_ = 50.41 µM) > **19 _b_** (IC_50_ = 73.38 µM) > **20_c_** (IC_50_ = 144.29 µM) > **23_a_** (IC_50_ = 152.93 µM) > **20_d_** (IC_50_ = 221.38 µM).
2. PC3: The descending order of the tested derivatives was as follows: **15 _b_** (IC_50_ = 25.58 µM) > **15_a_** (IC_50_ = 30.28 µM) > **15_d_** (IC_50_ = 40.90 µM) > **14_a_** (IC_50_ = 50.67 µM) > **15_c_** (IC_50_ = 55.95 µM) > **19 _b_** (IC_50_ = 84.41 µM) > **20_c_** (IC_50_ = 157.00 µM) > **23_a_** (IC_50_ = 221.31 µM) > **20_d_** (IC_50_ >300 µM).
3. MCF-7: The descending order of the tested derivatives was as follows: **15 _b_** (IC_50_ = 19.88 µM) > **15_d_** (IC_50_ = 33.40 µM) > **15_a_** (IC_50_ = 47.10 µM) > **14_a_** (IC_50_ = 58.26 µM) > **15_c_** (IC_50_ = 62.68 µM) > **19 _b_** (IC_50_ = 105.48 µM) > **20_c_** (IC_50_ = 134.85 µM) > **23_a_** (IC_50_ = 233.92 µM) > **20_d_** (IC_50_ = 246.20 µM).

It can be noticed that compound **15_b_** was ranked first in terms of activity against all the tested cancer cell lines. It showed potency equal to 0.74, 0.76, and 0.73 of sorafenib potency against HepG2, PC3, and MCF-7, respectively. Meanwhile compound **15_d_** came second against HepG2 and MCF-7 and third against PC3. The second compound against PC3 was **15_a_** which came third against both HepG2 and MCF-7. The other derivatives displayed the same descending order of activity against all cancer cell lines as follows: **14_a_** > **15_c_** > **19 _b_** > **20_c_** > **23_a_** > **20_d_**.

Of note, the cytotoxicity against normal human lung fibroblasts cell line (WI-38) were examined for the nine selected compounds. It is evident from Table 2 that the tested compounds have a significantly higher IC_50_ versus WI-38 than the IC_50_ versus cancer cell lines. It was found that compounds **15_b_** and **15_d_** showed the best selectivity. Compound **15_b_** showed selectivity indices of 13.57, 9.50, and 11.87 to HepG2, PC3, and MCF-7 cell lines, respectively, while it was found that selectivity indices of compound **15_d_** were 9.22, 5.53, and 6.92 to the cell lines, respectively.

On the basis of the obtained data, Compound **15_d_** was the most promising derivative in VEGFR-2 inhibitory testing and showed promising antitumor activity against HepG2 cells with good selectivity. So that the effects of **15_d_** on cell cycle and apoptosis rate of HepG2 were evaluated.

#### 3.2.3. Effect of 15_d_ on cell cycle of HepG2

**Figure 4.**
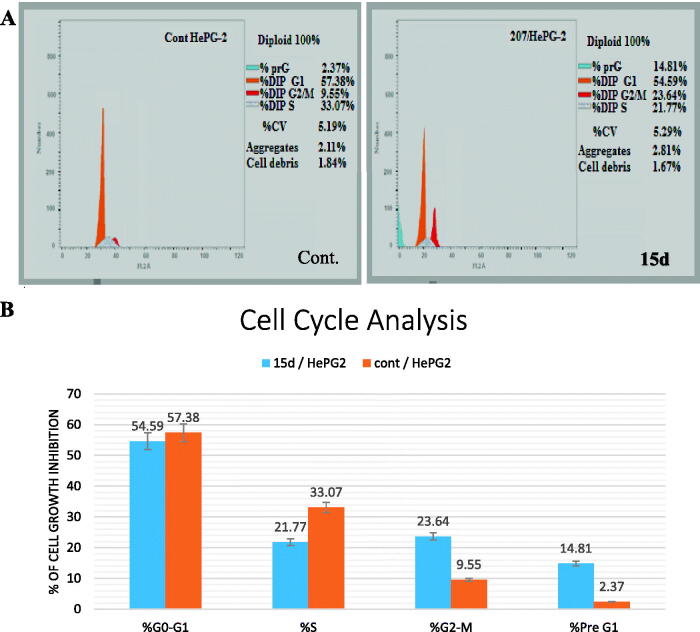
(A) HepG-2 cells distribution upon treatment with compound **15_d_.** (B) Percentages of cell cycle phases of HePG2 treated with **15_d_** in comparison with control.

HepG2 cell line was selected to evaluate the effect of compound **15_d_** on its different cell cycle phases as shown in Figure 4(A,B) where we can see that compound **15_d_ (**at its IC_50_) has the ability to stop the growth of HepG2 in both G2/M and pre G1 phases leading to significant increase in cell accumulation percentage.

#### 3.2.4. Effect of 15_d_ on apoptosis rate of HepG2

**Figure 5.**
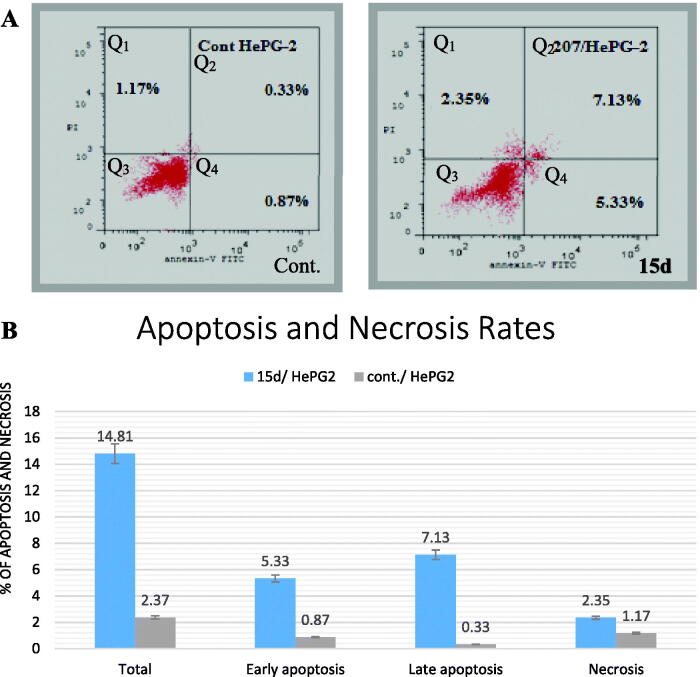
(A). Histogram for the effect of compound **15_d_** on both apoptosis and necrosis of HepG2 cells. (B) Column chart representing the effect of compound **15_d_** on both apoptosis and necrosis of HepG2 cells.

Table 3 and Figure 5(A,B) show that compound **15_d_** (at IC_50_) caused a 10 folds increase in apoptosis of HepG2 from 1.20 to 12.46%. We can see the significant increase in the percentages of early apoptosis from 0.87 to 5.33% and late apoptosis from 0.33 to 7.13%, while necrosis was slightly increased from 1.17 to 2.35%. Furthermore, it is also noted that in the control cells, induction of necrosis was almost at the same rate of apoptosis. But when the cells were treated with compound **15_d_**, apoptosis became about five times higher than necrosis.

**Table 3.** Percentage induction of HepG2 apoptosis and necrosis by compound **15_d_** compared with control.

S	Comp. no.	Total	% Apoptosis		% Necrosis
			Early	Late	
1	**15_d_**/ HepG2	14.81	5.33	7.13	2.35
2	cont./ HepG2	2.37	0.87	0.33	1.17

#### 3.2.5. Effect of compound 15_d_ on apoptosis markers

**Figure 6.**
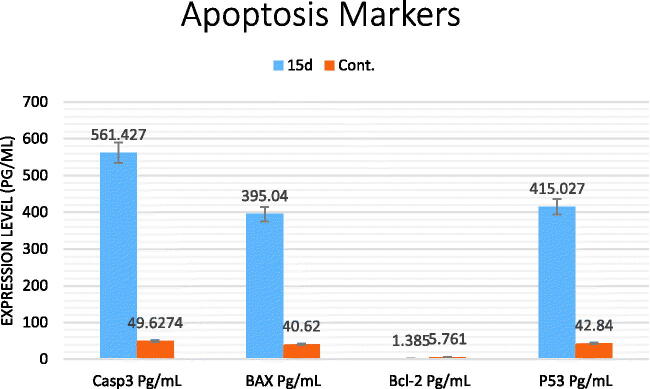
The effect of compound **15d** on apoptotic markers of HepG2 cells.

Table 4 and Figure 6 illustrate that compound **15_d_** (at IC_50_) caused remarkable increase in apoptotic markers (i.e. Caspase-3, BAX, and P53) in HepG2 cells. We can see the significant increase in the expression levels of these markers from 49.6274, 40.62, and 42.84 to 561.427, 395.04, and 415.027 pg/mL, respectively. Meanwhile, considerable decrease was seen in the level of Bcl-2 from 5.761 to 1.385 pg/mL. These obtained data gave an explanation for the ability of compound **15_d_** to induce apoptosis in cancer cells.

**Table 4.** Percentage of apoptotic markers of HepG2 cells by compound **15d** compared to control.

Comp. no.	Casp3 (pg/mL)	BAX (pg/mL)	Bcl-2 (pg/mL)	P53 (pg/mL)
**15_d_**/HepG2	561.427	395.04	1.385	415.027
cont./HepG2	49.6274	40.62	5.761	42.84

#### 3.2.6. Multi-kinase assay

Further exploration for the activity of compound **15_d_** was performed against two other types of kinases: human epidermal growth factor receptor 2 (HER2) and fibroblast growth factor receptor (FGFR). Table 5 shows the obtained IC_50_ of compound **15_d_** compared to erlotinib as a reference drug. It was found that IC_50_ of the **15_d_** is much higher for HER2 and FGFR than erlotinib. However, the new derivative was able to show considerable inhibition for HER2 and FGFR with IC_50_ = 253 and 381 nM, respectively.

**Table 5.** IC_50_ of HER2 and FGFR for compound **15_d_** compared to erlotinib.

Comp. no.	HER2 (nM)	FGFR (nM)
**15_d_**	253 ± 1.6	381 ± 1.8
Erlotinib	95 ± 1.2	34 ± 0.92

### 3.3. Molecular docking

**Figure 7.**
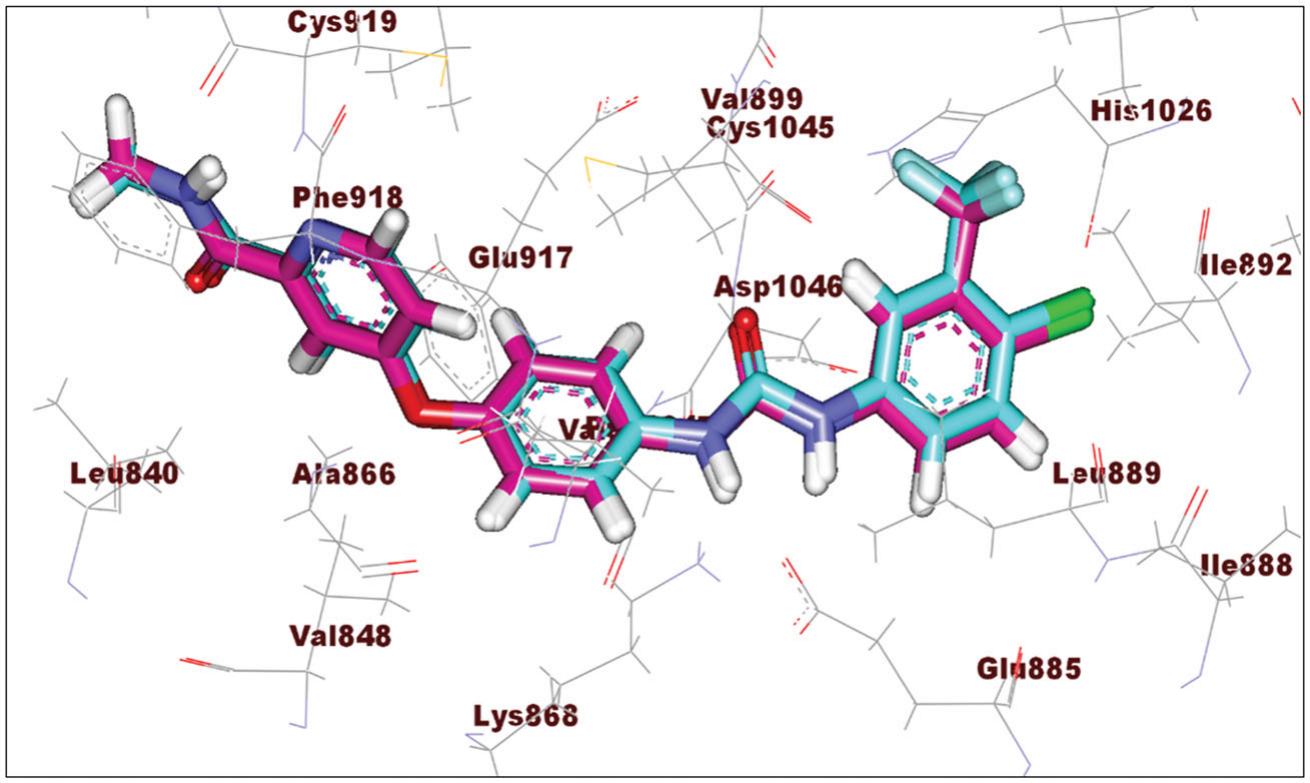
Overlay of the co-crystallized ligand (magenta) and the redocking one (turquoise) of sorafenib into VEGFR-2 kinase active site.

Using the C-Docker protocol in the Discovery Studio 2.5 software for studying docking mood of the new molecules and their orientation in VGEFR-2 kinase (PDB ID: (4ASD)) using sorafenib as a reference ligand. The docking was validated by incorporating the C-Docker algorithm to redock the co-crystallized ligand. RMSD obtained was 0.59 which indicated the validation of the applied docking protocol (Figure 7).

In this section of the work, we consider the binding free energy (**ΔG)** and the binding modes of the new derivatives. Critical interactions with various key residues, including Cys919 in the hinge region and Glu885 and Asp1046 in the DFG-binding domain were reported for potent VEGFR-2 inhibitors^32^^,^^51^. The docking results demonstrated that the studied molecules displayed correct binding patterns into the active site of VEGFR-2 kinase. At the same time, there was a significant correlation between the biological results and ΔG of the new derivatives. Table 6 shows the values of ΔG for the new derivatives as well as for the active control (sorafenib).

**Table 6.** Binding free energies (calculated ΔG in C-Docker energy score) of the studied compounds and sorafenib as a positive control into VEGFR-2.

Comp. No.	ΔG **[**C-Docker energy score**]**	Comp. No.	ΔG **[**C-Docker energy score**]**
**14_a_**	–63.86	**20_b_**	–39.61
**14_b_**	–52.41	**20_c_**	–45.16
**15_a_**	–61.91	**20_d_**	–53.75
**15_b_**	–53.63	**22_a_**	–44.59
**15_c_**	–66.52	**22_b_**	–45.99
**15_d_**	–64.11	**23_a_**	–49.31
**19_a_**	–51.77	**23_b_**	–39.17
**19_b_**	–55.43	**23_c_**	–37.21
**20_a_**	–40.06	Sorafenib	–74.23

**Figure 8.**
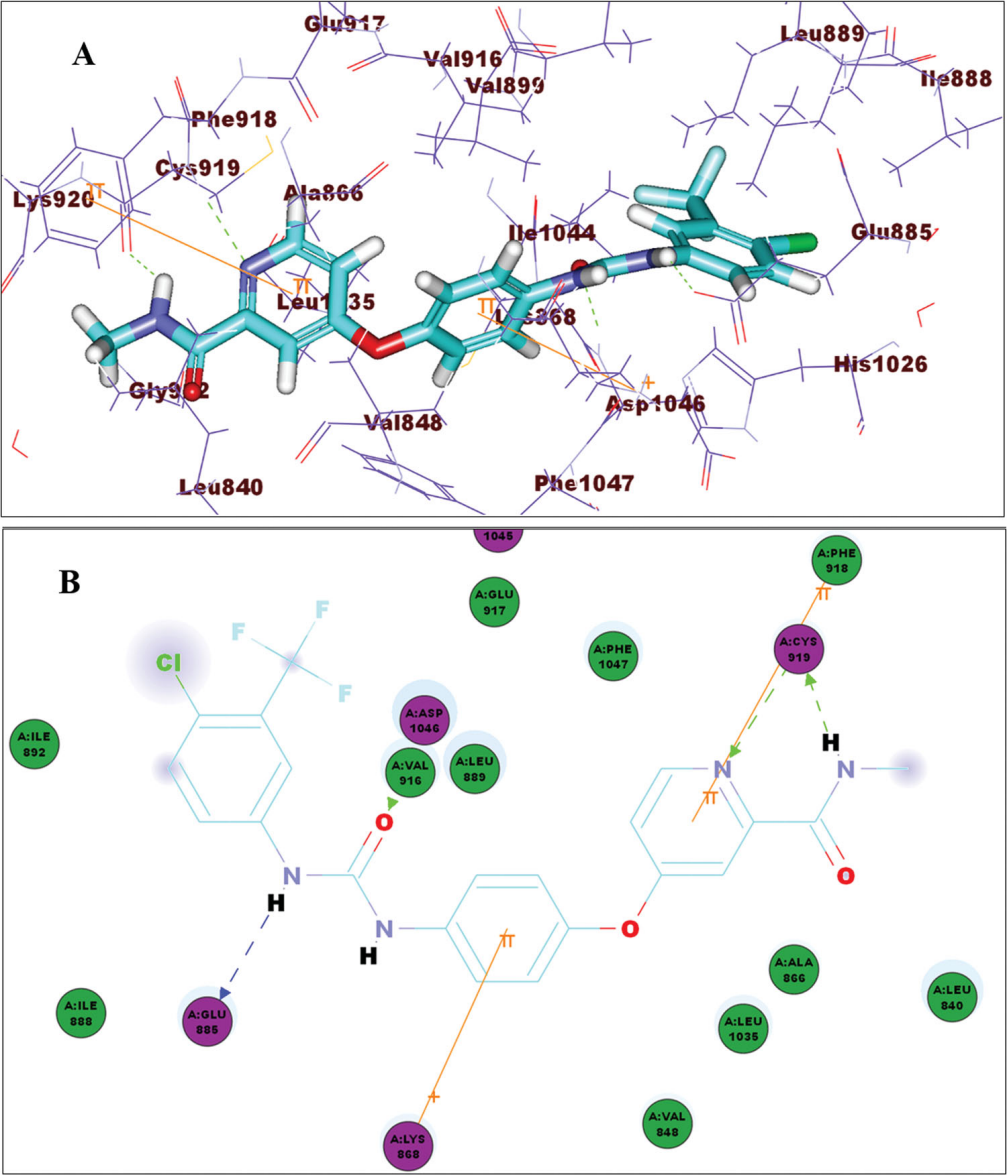
(A) 3D of sorafenib binding mode. (B) 2D of sorafenib binding mode.

The proposed sorafenib binding mode as a reference ligand revealed an affinity value of –74.23 C-Docker energy score. *N*-methylpicolinamide moiety was found to form two hydrogen bonds with Cys919 and an aromatic stacking interaction (pi–pi interaction) with Phe918 in the Hinge region. Also, the phenoxy group was observed in the linker region forming cation–pi interaction with Lys868. Urea moiety, meanwhile, displayed two hydrogen bonds with Glu885 and Asp1046 in the DFG binding domain. Finally, the terminal substituted phenyl moiety accommodated the allosteric region formed by Lue889, Glu885, His1026, Leu1019, and Ile888. The results obtained were consistent with the reported^32^^,^^51^ (Figure 8(A,B).

**Figure 9.**
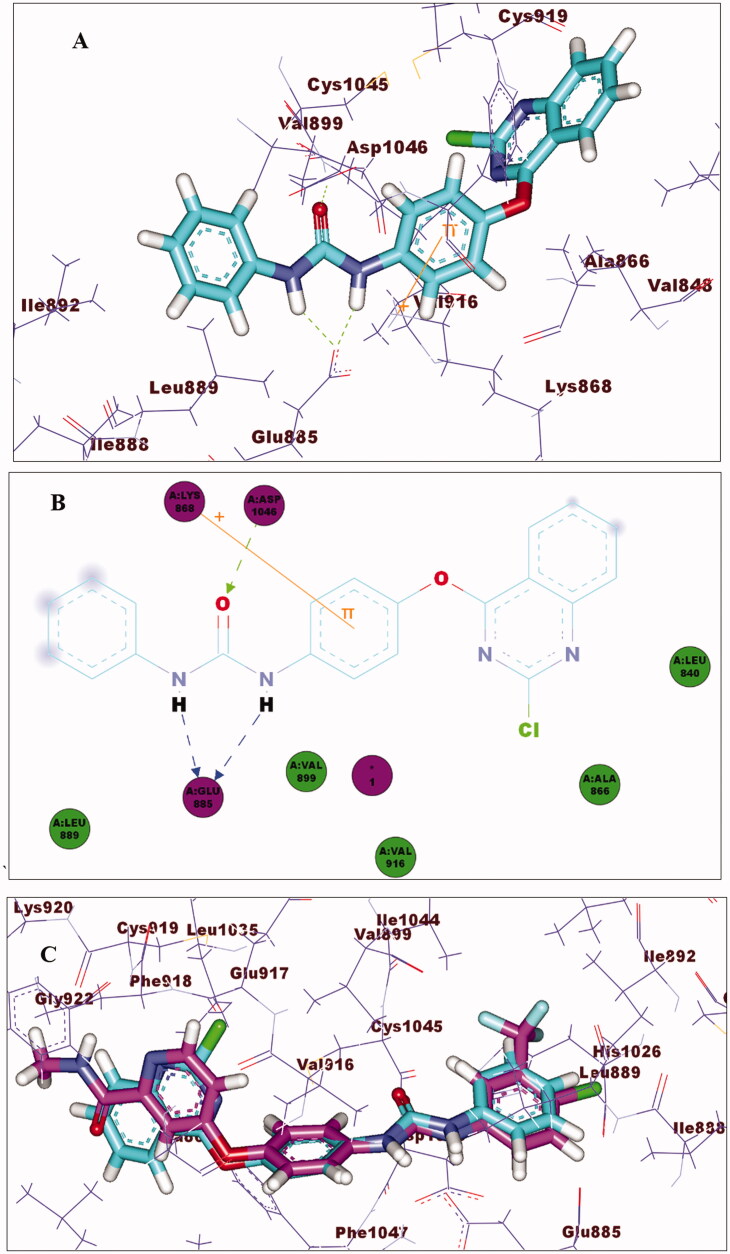
(A) 3D of compound **14_a_** binding pattern (B) 2D of compound **14_a_** binding pattern (C) Superimposing **14_a_** (turquoise) and sorafenib (magenta).

With regard to the new molecules, compound **14_a_** revealed a high binding affinity (ΔG = –66.86). This high affinity for VEGFR-2 is likely due to the formation of three hydrogen bonds as illustrated in Figure 9(A,B). As can be seen from Figure 9, the quinazoline moiety correctly occupied the Hinge region. Additionally, the phenoxy group was fitted to the linker region and formed cation–pi interaction with Lys868. It is also evident from Figure 9 that the two N-H portions of urea moiety showed two hydrogen bonds with Glu885 in the DFG binding domain, while oxygen atom of the carbonyl displayed one hydrogen bond with Asp1046. Furthermore, the terminal phenyl group oriented to the allosteric region showed hydrophobic interactions with Leu889, Ile888, Ile892, and Val899. The spatial orientation of compound **14_a_** in comparison with sorafenib is shown in Figure 9(C).

**Figure 10.**
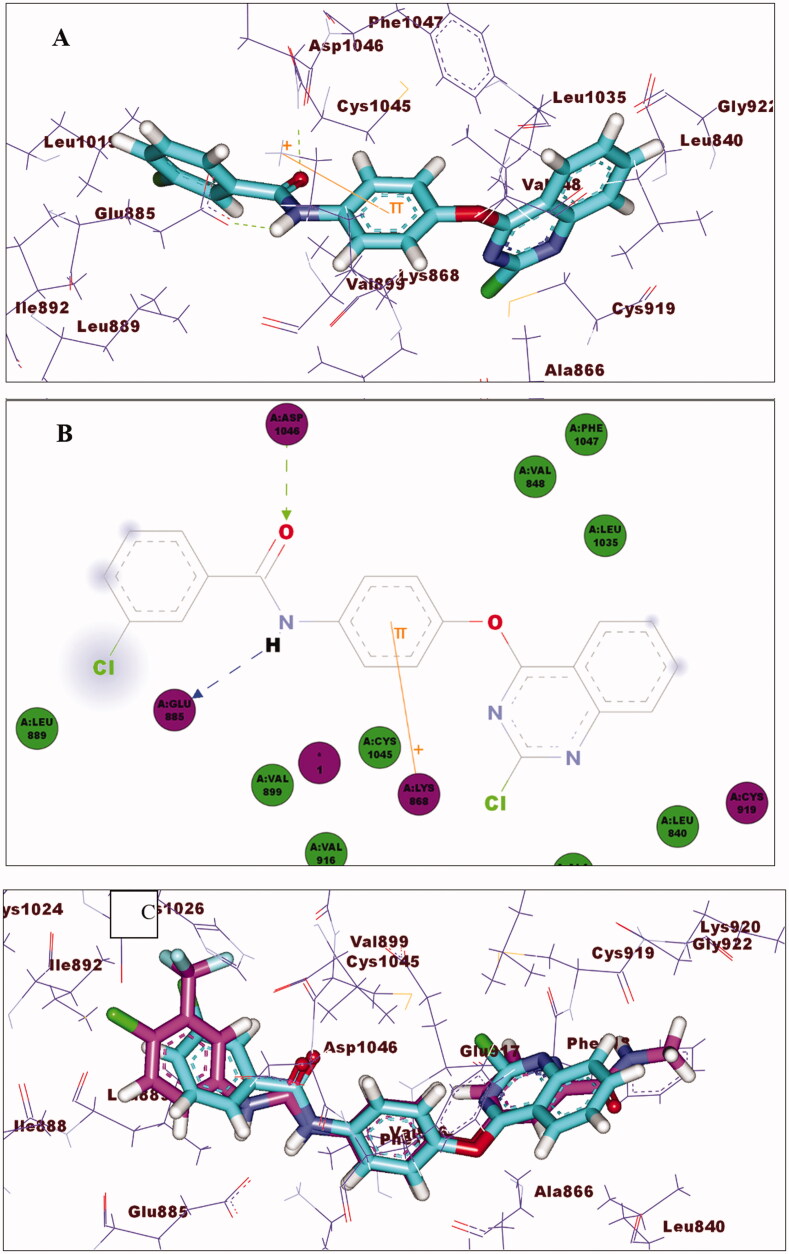
(A) 3D of **15_c_** binding pattern. (B) 2D of **15_c_** binding pattern. (C) Superimposing **15_c_** (turquoise) and sorafenib (magenta).

Compound **15_c_** exhibited a good fitting to VEGFR-2 with binding energy of 66.52 C-Docker energy score. The quinazoline moiety accommodated the Hinge region as shown in Figure 10(A,B). The phenoxy group was fitted to the linker region and exhibited cation–pi interaction with Lys868. In DFG region, the amide showed two hydrogen bonding interactions with Asp1046 and Glu885. Furthermore, the terminal 3-chlrophenyl group occupied the allosteric binding region and formed hydrophobic interactions with Leu889, Ile892, and Lue1019. Figure 10(C) shows a superimposition of sorafenib and **15_c_** in order to compare their spatial orientations.

**Figure 11.**
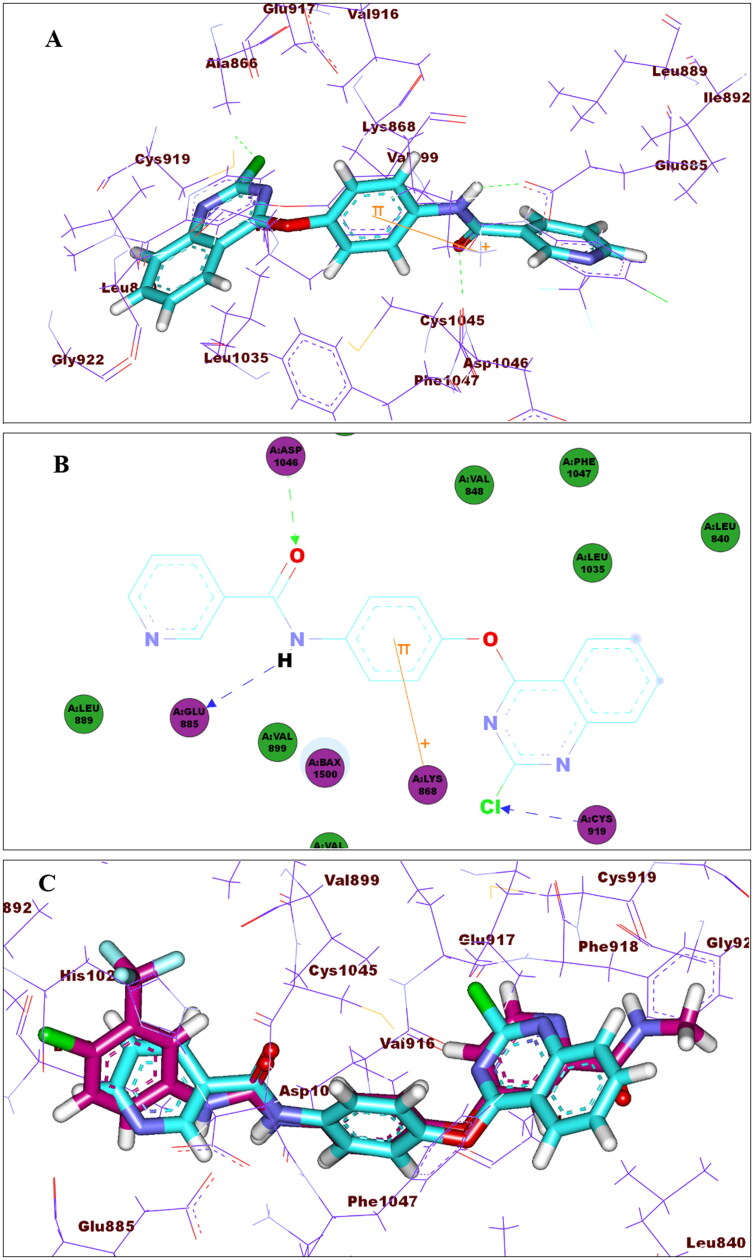
(A) 3D of compound **15_d_** binding pattern (B) 2D of compound **15_d_** binding pattern (C) Overlay of **15_d_** (turquoise) and sorafenib (magenta).

Investigation of the best pose of compound **15_d_** (–64.11 C-Docker energy score) demonstrated the formation of hydrogen bonds as well as electrostatic and hydrophobic interactions. Figure 11(A,B) shows pi–cation interaction between the phenoxy group and Lys868 of the linker region. Meanwhile, N-H of the amide group demonstrated a hydrogen bond with Glu885, and the oxygen of carbonyl group formed anther hydrogen bond with Asp1046 at the DFG binding domain. Moreover, the chloride atom at position 2 of quinazoline formed hydrogen bond with Cys919 of the ATP-binding domain. Finally, pyridyl moiety accommodated the allosteric binding region showing hydrophobic interactions. Figure 11(C) shows a superimposition of **15_d_** and sorafenib.

The docking results revealed the ability of the new derivatives to accommodate VEGFR-2 pocket and form interactions in a mode similar to that of sorafenib. These results are not only consistent with biological data obtained from the VEGFR-2 assay but also give an explanation to them. For example, quinazoline based derivatives (**14_a,b_** and **15_a–d_**) displayed the best docking energy (from –53.63 to –66.52 C-Docker energy score) as presented in Table 6. Regarding VEGFR-2 inhibition, the same derivatives were the most potent (IC_50_ ranged from 60 to 94.22 nM) as listed in Table 1. The most potent candidates for VEGFR-2 inhibition were **15_d_** and **15_c_** with IC_50_ = 60.00 and 65.24 nM, respectively. Similarly, these two compounds were the most promising in terms of docking score showed the highest binding free energies 64.11 and 66.52 C-Docker energy score, respectively. At the same time, quinoxaline-based derivatives (**19_a,b_** and **20_a–d_**) came the second with regard to both VEGFR-2 inhibition and docking score as illustrated in Tables 1 and 6. It can also be seen that nitrobenzene-based derivatives came last in both biological activity and docking results.

### 3.4. Structure–activity relationship (SAR)

According to the results given, the following relationships can be established for the new derivatives

1. Quinazoline nucleus as a planar heteroaromatic moiety is much better than both quinoxaline and benzene ring. It can be noticed from Table 1 that quinazoline-based derivatives (**14_a&b_** and **15_a–d_**) showed IC_50_ values ranging from 60 to 94.22 nM. These results were the best compared to other candidates which showed IC_50_ values ranging from 69.55 to 123.85 nM. Similarly, the antiproliferative assay data showed that quinazoline-containing derivatives (**14_a_** and **15_a–d_**) were the most promising against all the tested cancer cell lines.
2. The nitrogen atom in the heteroaromatic moiety plays a key role in the activity. For example, the data of VEGFR-2 inhibitory assay revealed IC_50_ = 67.05 nM for **14_a_** which is quinazoline-based candidate. Meanwhile, the corresponding nitrobenzene-based derivative (**22_a_**) displayed IC_50_ = 123.85 nM. In a similar manner, compound **15_a_** (quinazoline derivative) showed significantly better antiproliferative results than **23_a_** (a nitrobenzene derivative).
3. Urea, as well as amide moiety, is an effective hydrogen bond acceptor and donor. IC_50_ for compound **14_a_** and its corresponding amide derivative (**15_a_**) were found to be 67.05 and 67.25 nM against VEGFR-2, respectively. At the same time, both displayed antiproliferative activity.
4. With respect to hydrophobic moiety
5. Both aromatic systems and aliphatic groups can accommodate VEGFR-2 pocket with priority given to aromatic systems. Compounds **14_a_** and **14_b_** exhibited IC_50_ = 67.05, 94.22 nM, respectively.
6. Substituted aromatic systems are better than unsubstituted ones. Derivatives containing 4-chlorophenyl moiety as **15_b_** displayed far more potent antiproliferative activity than **15_a_** which contain plain phenyl ring.
7. Pyridine nucleus is better than benzene ring. The nitrogen atom increased the basicity of the pyridine ring than benzene ring which makes a chance to form pi-cation interaction more than benzene ring. Figures 12(A,B) and 13(A,B) in Supplementary data. Moreover, compound **15_d_** was found to be the most promising derivative as VEGFR-2 inhibitor with IC_50_ = 60.00 nM. It also demonstrated antiproliferative activity better than that of **15_a_** (Table 1).

## 4. Conclusion

On the basis of quinazoline, quinoxaline, and benzene nuclei, we designed and synthesised 17 new compounds in order to meet the reported pharmacophoric features of type II VEGFR-2 inhibitors. Biological results showed that compounds **15_d_** and **15_b_** are of particular interest as anticancer agents targeting VEGFR-2 kinase. In addition to their considerable inhibition of VEGFR-2, they have shown promising antitumor effects especially against hepatocellular cancer cell line (HepG2) with high degree of selectively. The data obtained from VEGFR-2 inhibitory assay revealed IC_50_ = 60.00 and 86.36 nM for **15_d_** and **15_b_**, respectively compared to IC_50_ = 54.00 nM for the positive control sorafenib. At the same time, the IC_50_ values obtained for the two candidates against HepG2 were 24.10 and 17.39 µM, respectively. Further examination of compound **15_d_** revealed its importance as an apoptosis inducer at HepG2 cells where it raised the apoptosis rate from 1.20 to 12.46%. The mechanism of apoptosis induction was proved to compound **15_d_** through increasing the expression levels of caspase-3, BAX, and P53. Additionally, Bcl-2 level was decreased. Accordingly, this work suggests that compounds **15_b_** and **15_d_** should be considered for further evaluation and/or modification as promising anticancer candidates.

## Supplementary Material

Supplemental Material
