# Supplementary material for: Synthesis, biological evaluation, and molecular docking of new series of antitumor and apoptosis inducers designed as VEGFR-2 inhibitors
Source: J Enzyme Inhib Med Chem. 2022 Jan 10;37(1):573–91. doi: 10.1080/14756366.2021.2017911 (PMC8757611; doi:10.1080/14756366.2021.2017911)
Supplement: Supplemental Material [file IENZ_A_2017911_SM1984.pdf]

Majed ElWard_H_Sor209

Microanalytical Unit - FOPCU - NMR laboratory
www.pharma.cu.edu.eg dir-mau.fopcu@pharma.cu.edu.eg

BRUKER

```
Current Data Parameters
NAME      Majed ElWard_H_Sor209
EXPNO                    10
PROCNO                    1

F2 - Acquisition Parameters
Date_              20200311
Time                   6.39
INSTRUM               spect
PROBHD    5 mm PABBO BB/
PULPROG                zg30
TD                    65536
SOLVENT                DMSO
NS                       32
DS                        2
SWH                8012.820 Hz
FIDRES             0.122266 Hz
AQ                4.0894465 sec
RG                   202.37
DW                   62.400 usec
DE                     6.50 usec
TE                    298.1 K
D1               1.00000000 sec
TD0                       1

======== CHANNEL f1 ========
SFO1           400.1924713 MHz
NUC1                     1H
P1                    15.00 usec
PLW1            10.39999962 W

F2 - Processing parameters
SI                    65536
SF              400.1900000 MHz
WDW                      EM
SSB       0
LB                     0.30 Hz
GB        0
PC                     1.00
```

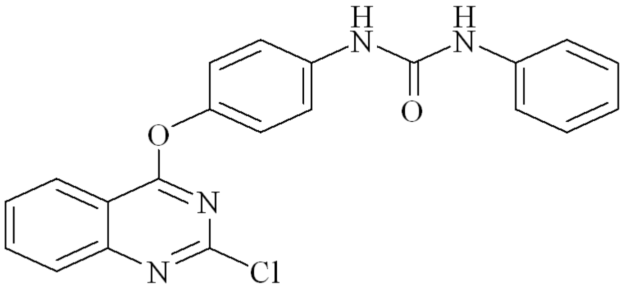

14a

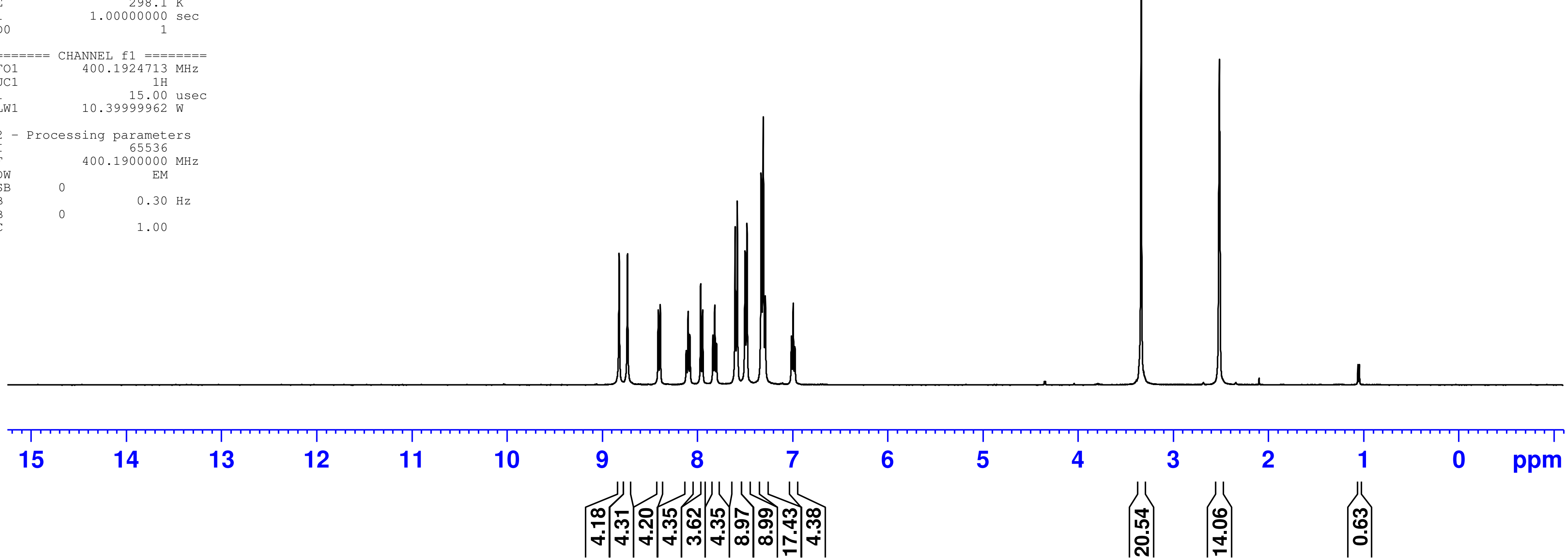

Majed ElWard_H_Sor211

Microanalytical Unit - FOPCU - NMR laboratory
www.pharma.cu.edu.eg dir-mau.fopcu@pharma.cu.edu.eg

```
Current Data Parameters
NAME      Majed ElWard_H_Sor211
EXPNO                  10
PROCNO                  1

F2 - Acquisition Parameters
Date_            20200311
Time                 6.58
INSTRUM             spect
PROBHD    5 mm PABBO BB/
PULPROG              zg30
TD                  65536
SOLVENT              DMSO
NS                     32
DS                      2
SWH              8012.820 Hz
FIDRES           0.122266 Hz
AQ              4.0894465 sec
RG                  56.39
DW                 62.400 usec
DE                   6.50 usec
TE                  298.1 K
D1             1.00000000 sec
TD0                     1

======== CHANNEL f1 ========
SFO1          400.1924713 MHz
NUC1                   1H
P1                  15.00 usec
PLW1          10.39999962 W

F2 - Processing parameters
SI                  65536
SF            400.1900000 MHz
WDW                    EM
SSB       0
LB                   0.30 Hz
GB        0
PC                   1.00
```

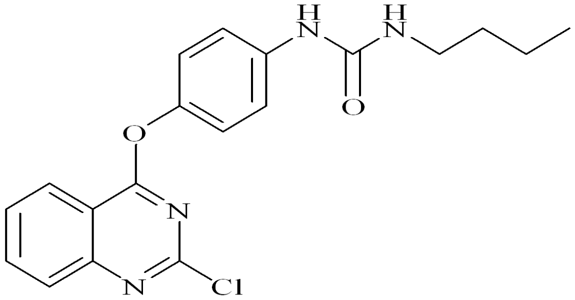

14b

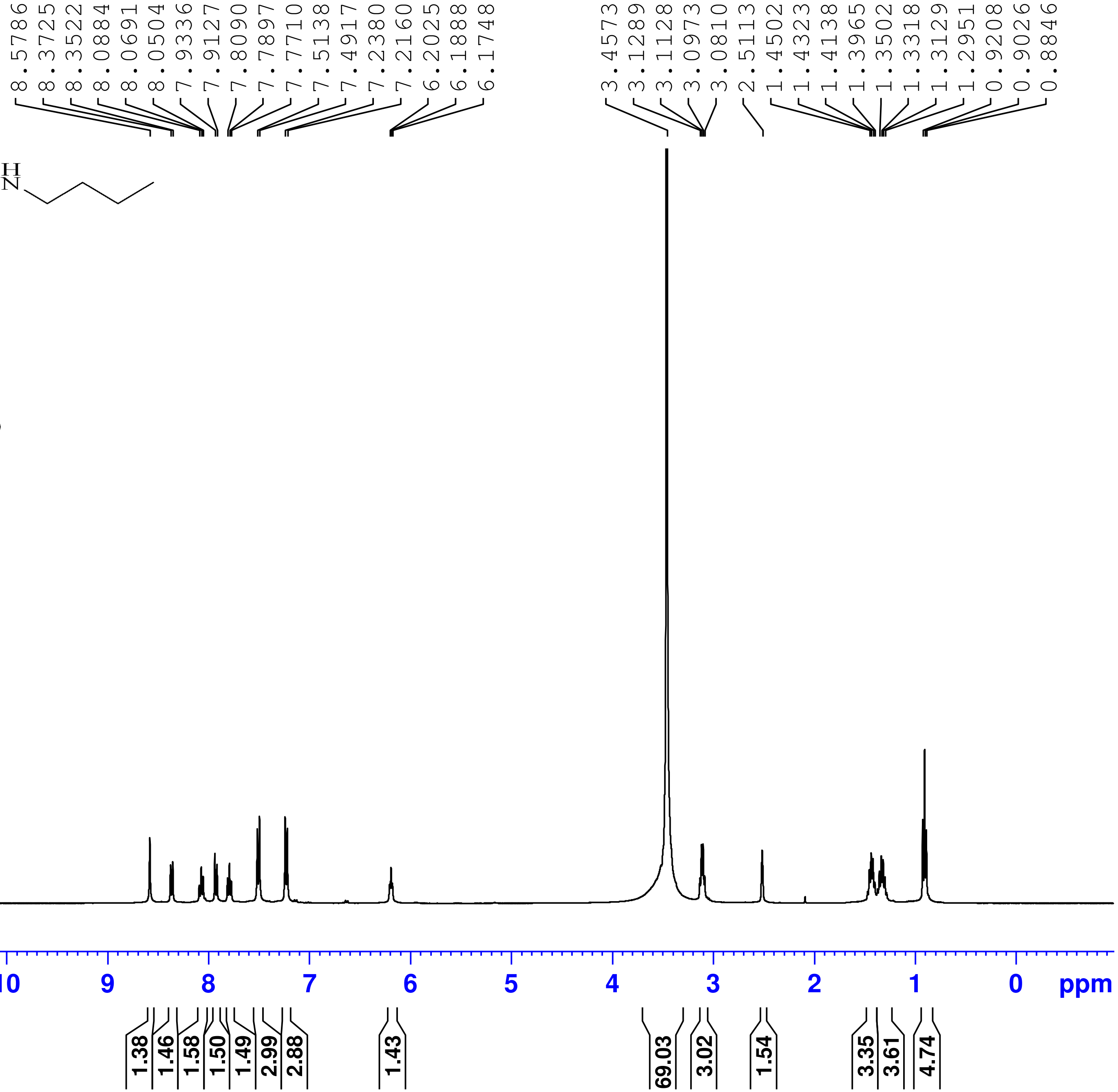

Majed ElWard_H_Sor201

Microanalytical Unit - FOPCU - NMR laboratory
www.pharma.cu.edu.eg dir-mau.fopcu@pharma.cu.edu.eg

BRUKER

```
Current Data Parameters
NAME       Majed ElWard_H_Sor201
EXPNO                     10
PROCNO                     1

F2 - Acquisition Parameters
Date_             20200311
Time                  6.02
INSTRUM              spect
PROBHD    5 mm PABBO BB/
PULPROG               zg30
TD                   65536
SOLVENT               DMSO
NS                      32
DS                       2
SWH               8012.820 Hz
FIDRES            0.122266 Hz
AQ               4.0894465 sec
RG                  202.37
DW                  62.400 usec
DE                    6.50 usec
TE                   298.1 K
D1              1.00000000 sec
TD0                      1

======== CHANNEL f1 ========
SFO1           400.1924713 MHz
NUC1                    1H
P1                   15.00 usec
PLW1           10.39999962 W

F2 - Processing parameters
SI                   65536
SF             400.1900000 MHz
WDW                     EM
SSB       0
LB                    0.30 Hz
GB        0
PC                    1.00
```

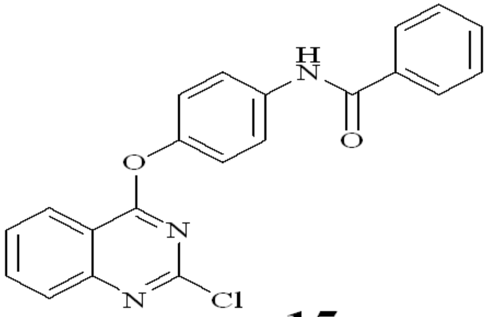

15a

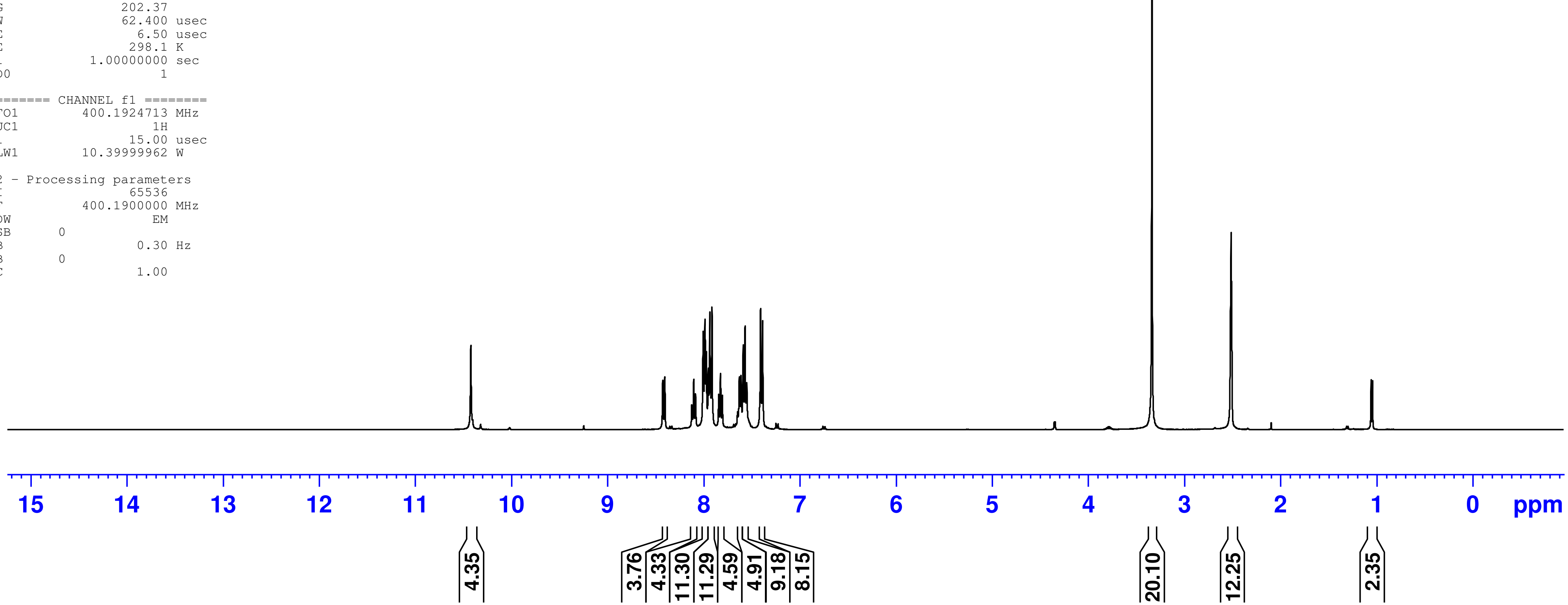

Abdallah ElSayed_H_Sor203.10.fid
Abdallah ElSayed_H_Sor203

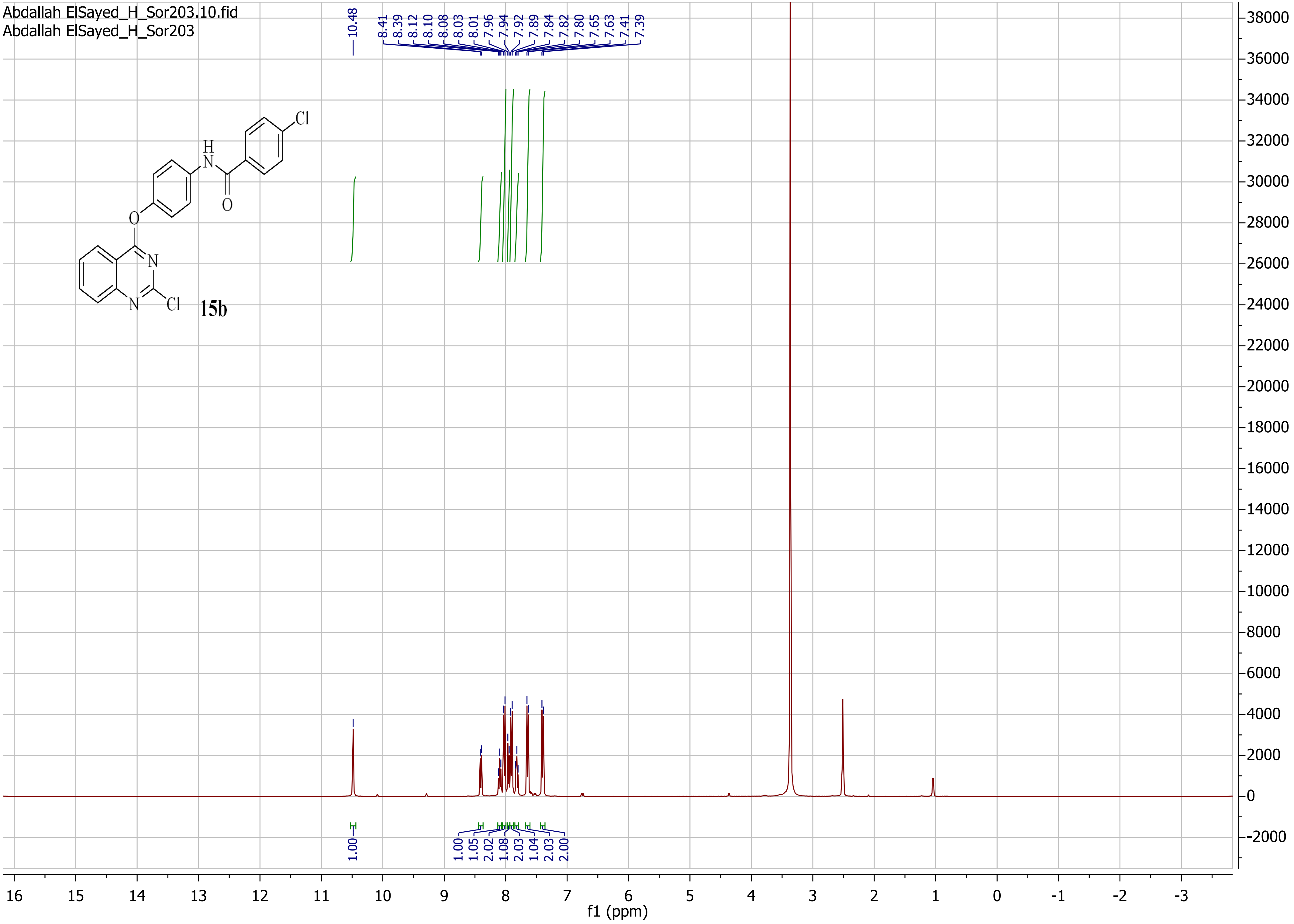

Majed ElWard_H_Sor205

Microanalytical Unit - FOPCU - NMR laboratory
www.pharma.cu.edu.eg dir-mau.fopcu@pharma.cu.edu.eg

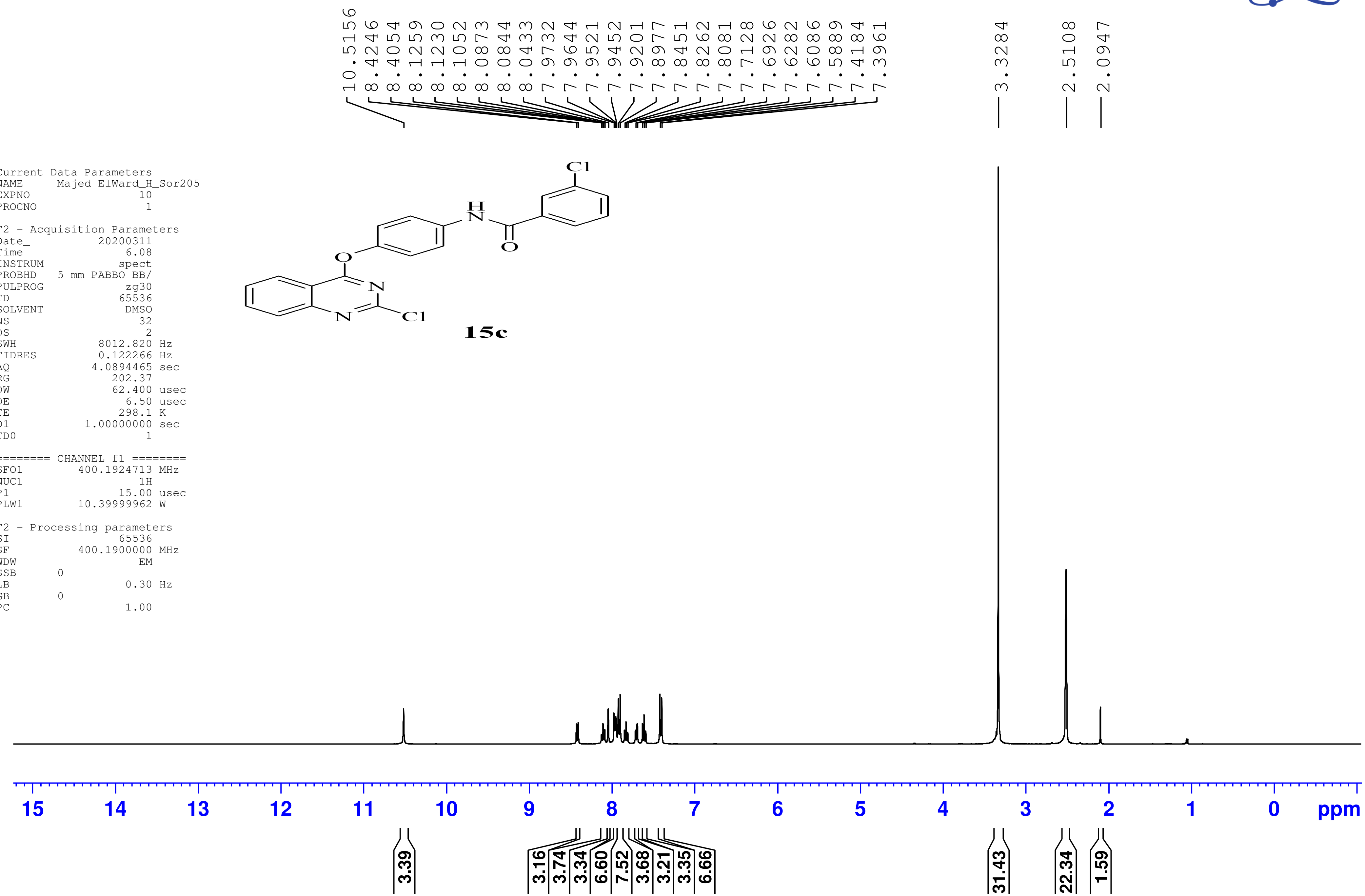

15c

```
Current Data Parameters
NAME      Majed ElWard_H_Sor205
EXPNO                     10
PROCNO                     1

F2 - Acquisition Parameters
Date_             20200311
Time                  6.08
INSTRUM              spect
PROBHD    5 mm PABBO BB/
PULPROG               zg30
TD                   65536
SOLVENT               DMSO
NS                      32
DS                       2
SWH               8012.820 Hz
FIDRES            0.122266 Hz
AQ               4.0894465 sec
RG                  202.37
DW                  62.400 usec
DE                    6.50 usec
TE                   298.1 K
D1              1.00000000 sec
TD0                      1

======== CHANNEL f1 ========
SFO1           400.1924713 MHz
NUC1                    1H
P1                   15.00 usec
PLW1           10.39999962 W

F2 - Processing parameters
SI                   65536
SF             400.1900000 MHz
WDW                     EM
SSB      0
LB                    0.30 Hz
GB       0
PC                    1.00
```

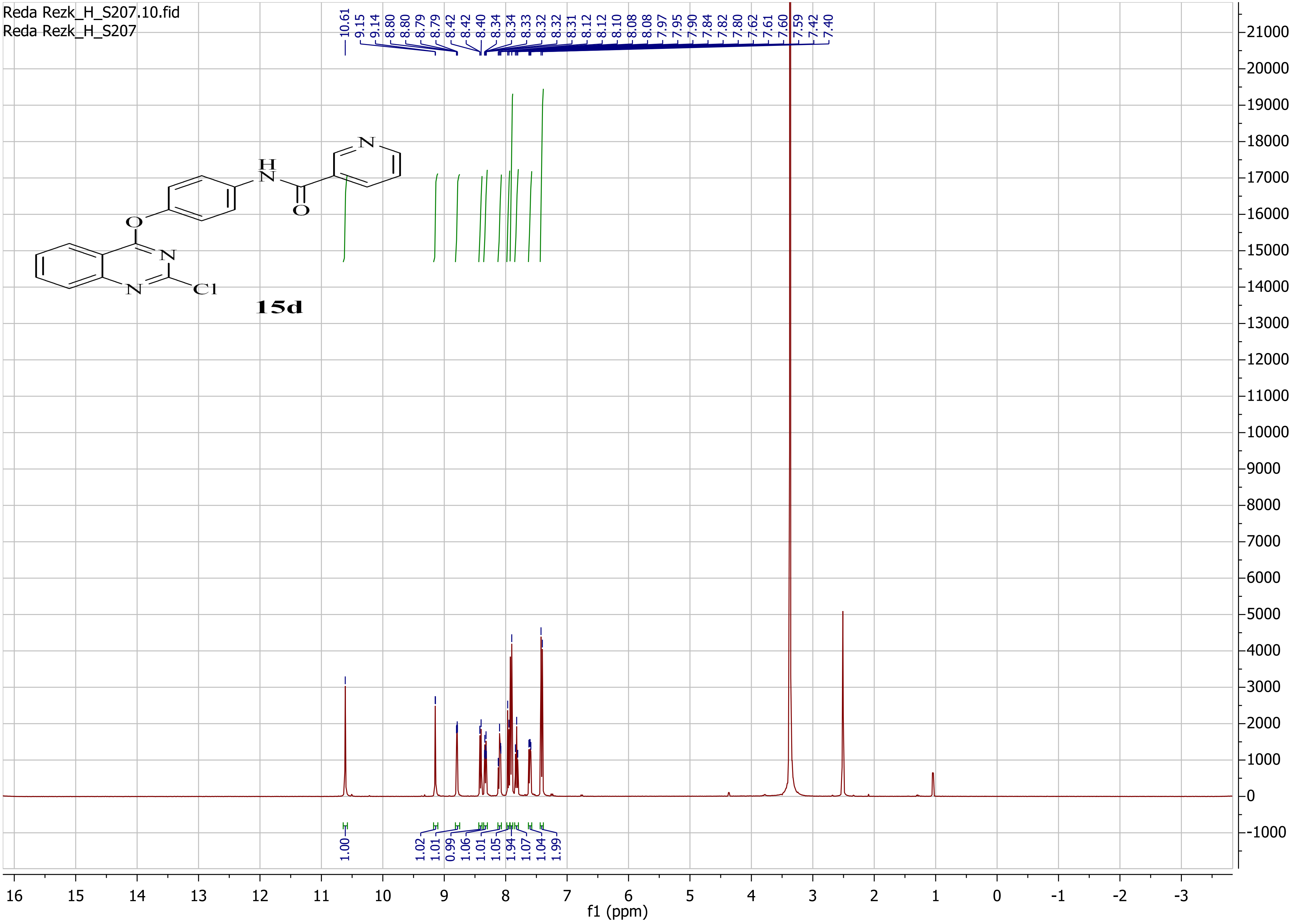
Reda Rezk_H_S207.10.fid
Reda Rezk_H_S207
10.61
9.15
9.14
8.80
8.80
8.79
8.79
8.42
8.42
8.40
8.34
8.34
8.33
8.32
8.32
8.31
8.12
8.12
8.10
8.08
8.08
7.97
7.95
7.90
7.84
7.82
7.80
7.62
7.61
7.60
7.59
7.42
7.40
15d
1.00
1.02
1.01
0.99
1.06
1.01
1.05
1.94
1.07
1.04
1.99
f1 (ppm)

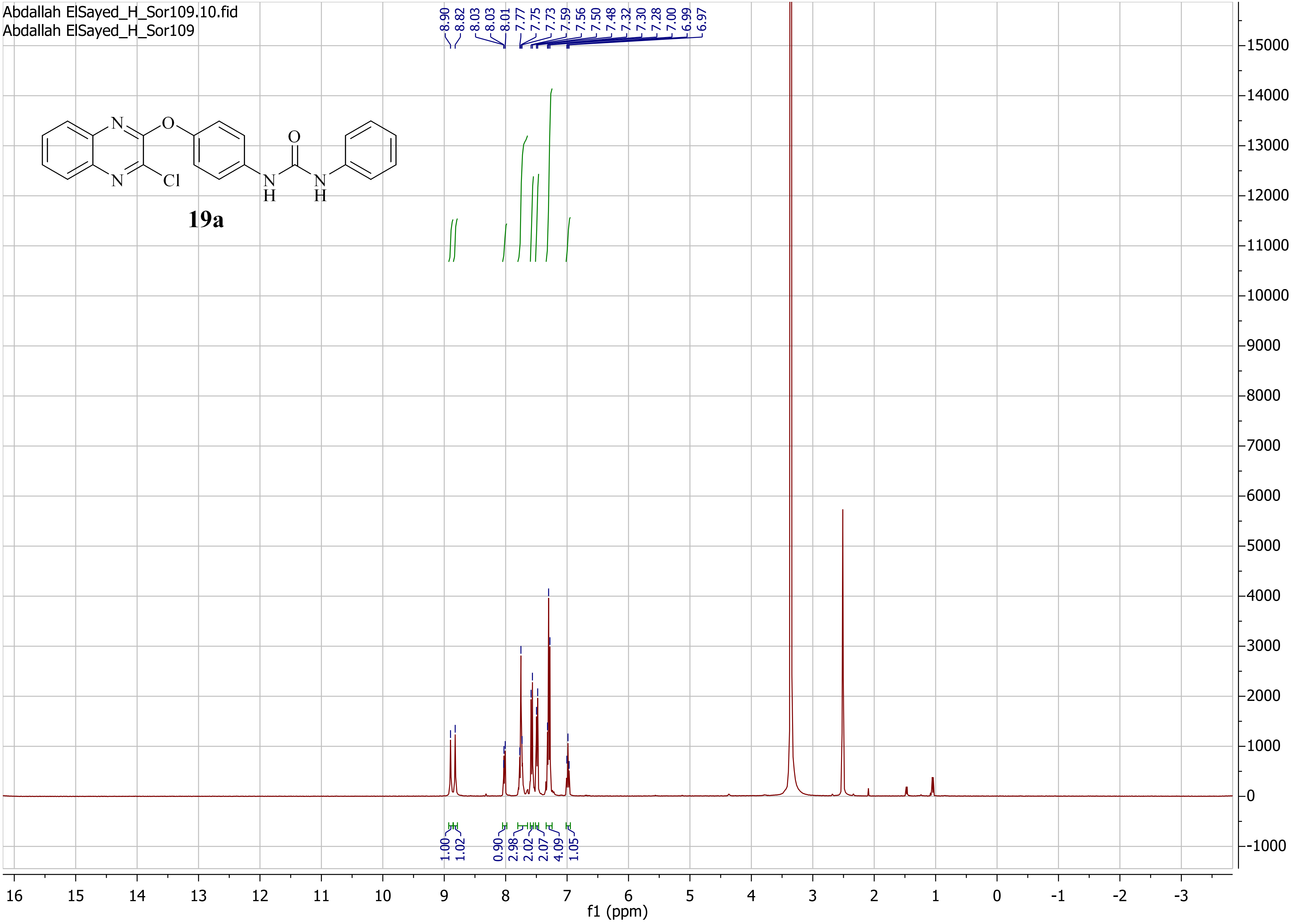
Abdallah ElSayed_H_Sor109.10.fid
Abdallah ElSayed_H_Sor109
8.90 8.82 8.03 8.03 8.01 7.77 7.75 7.73 7.59 7.56 7.50 7.48 7.32 7.30 7.28 7.00 6.99 6.97
19a
1.00 1.02 0.90 2.98 2.02 2.07 4.09 1.05
f1 (ppm)

Majed ElWard_H_Sor111

Microanalytical Unit - FOPCU - NMR laboratory
www.pharma.cu.edu.eg dir-mau.fopcu@pharma.cu.edu.eg

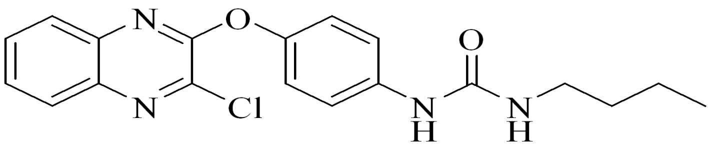

19b

```
Current Data Parameters
NAME      Majed ElWard_H_Sor111
EXPNO                   10
PROCNO                   1

F2 - Acquisition Parameters
Date_             20200311
Time                  6.52
INSTRUM              spect
PROBHD    5 mm PABBO BB/
PULPROG               zg30
TD                   65536
SOLVENT               DMSO
NS                      32
DS                       2
SWH               8012.820 Hz
FIDRES            0.122266 Hz
AQ               4.0894465 sec
RG                  202.37
DW                  62.400 usec
DE                    6.50 usec
TE                   298.1 K
D1              1.00000000 sec
TD0                      1

======== CHANNEL f1 ========
SFO1           400.1924713 MHz
NUC1                    1H
P1                   15.00 usec
PLW1           10.39999962 W

F2 - Processing parameters
SI                   65536
SF             400.1900000 MHz
WDW                     EM
SSB       0
LB                    0.30 Hz
GB        0
PC                    1.00
```

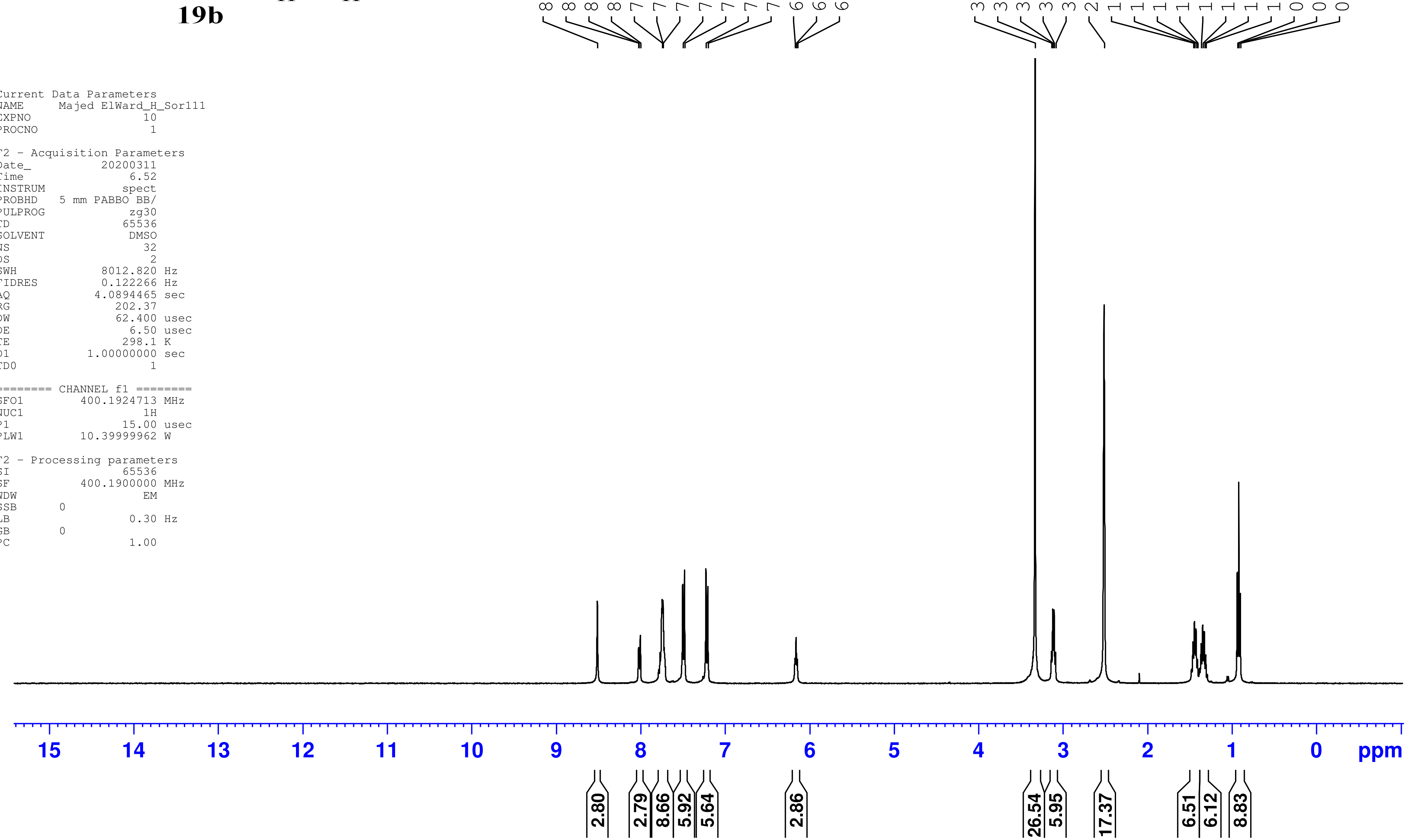

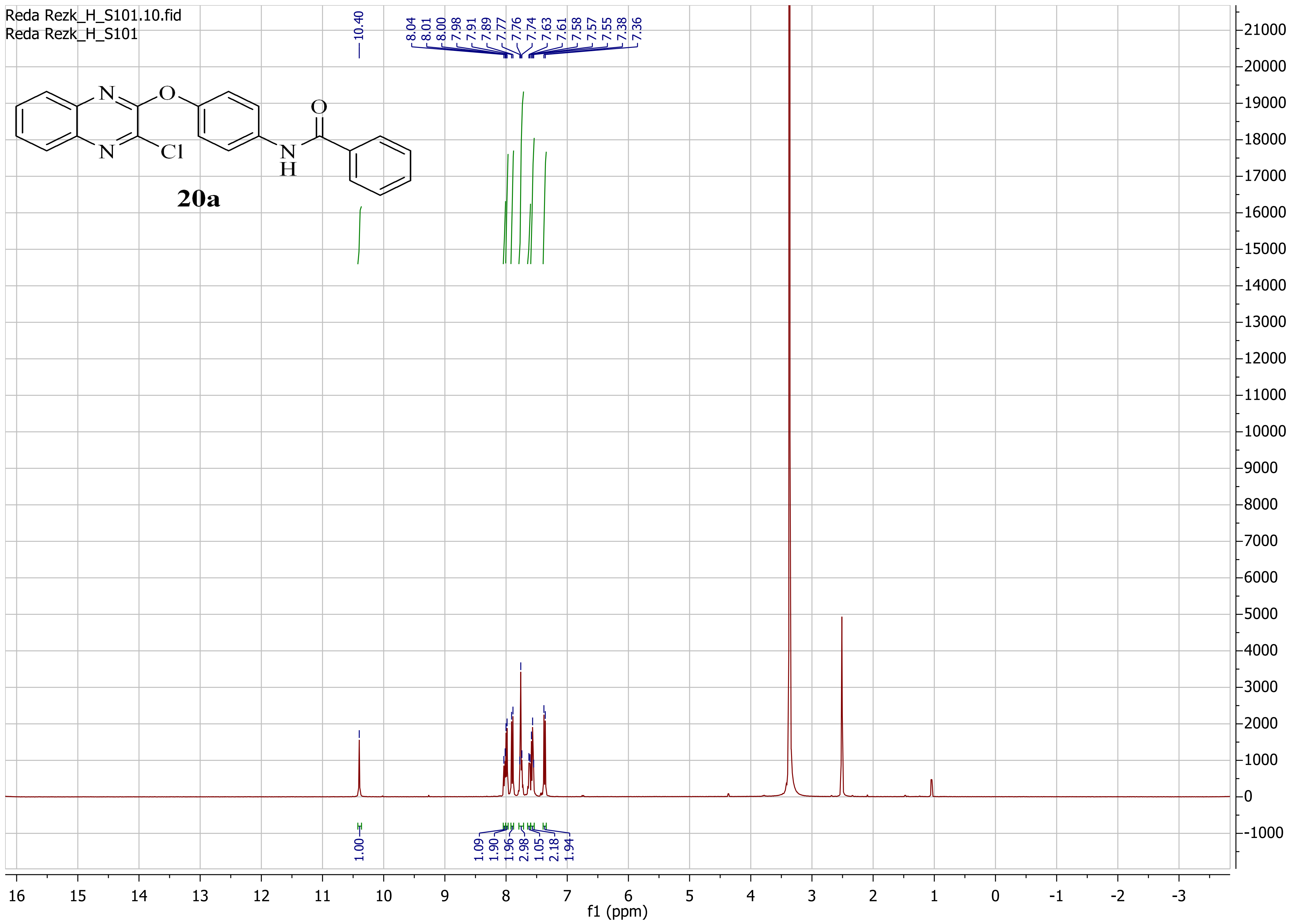

Reda Rezk_H_S101.10.fid
Reda Rezk_H_S101
20a
10.40
8.04
8.01
8.00
7.98
7.91
7.89
7.77
7.76
7.74
7.63
7.61
7.58
7.57
7.55
7.38
7.36
1.00
1.09
1.90
1.96
2.98
1.05
2.18
1.94
f1 (ppm)

Majed ElWard_H_Sor103

Microanalytical Unit - FOPCU - NMR laboratory
www.pharma.cu.edu.eg dir-mau.fopcu@pharma.cu.edu.eg

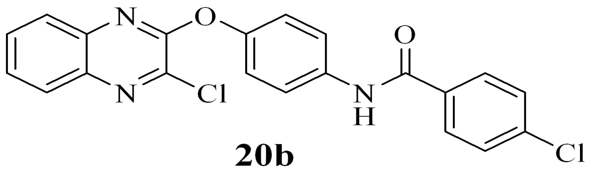

20b

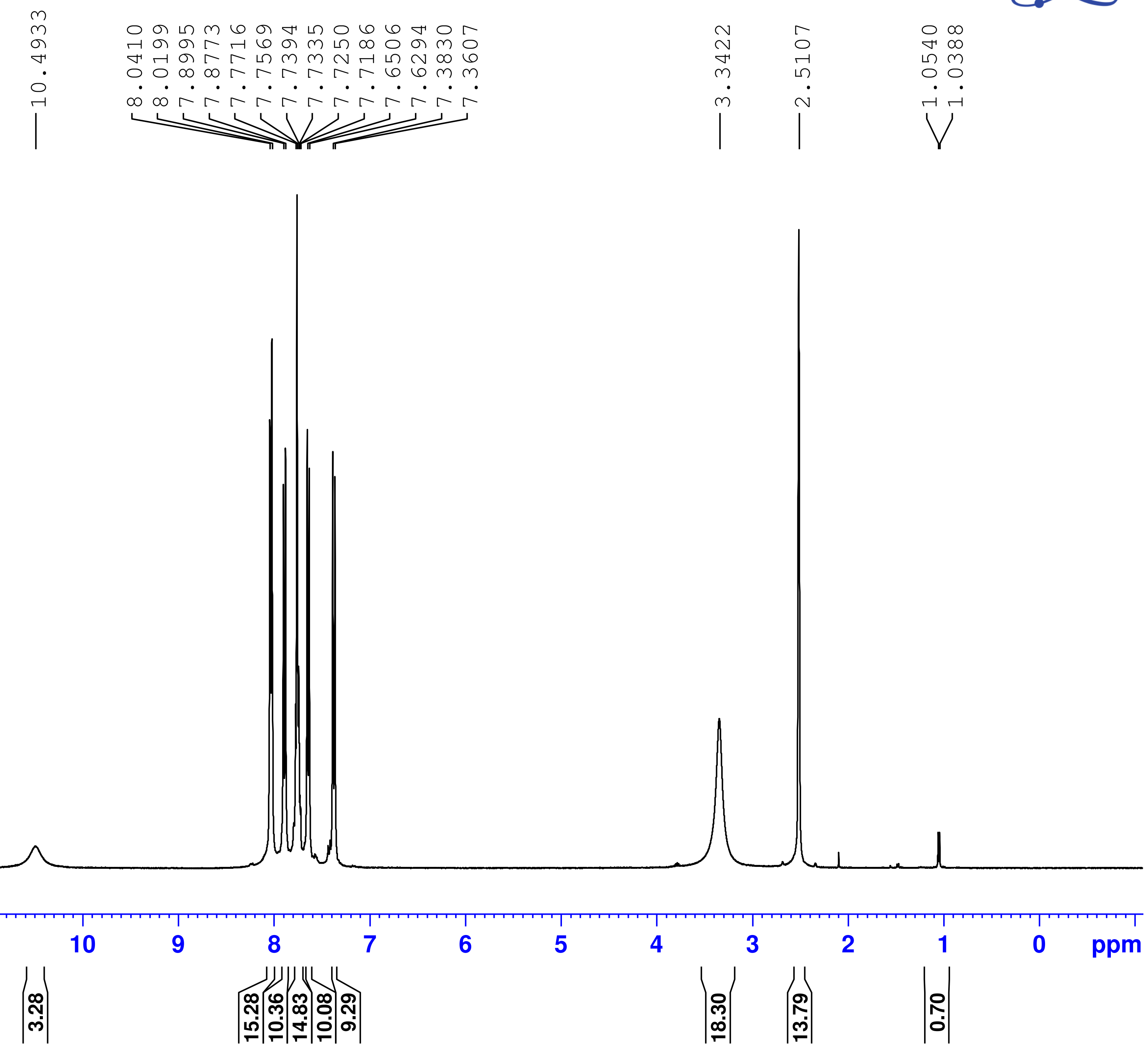


```
Current Data Parameters
NAME      Majed ElWard_H_Sor103
EXPNO                  10
PROCNO                  1

F2 - Acquisition Parameters
Date_          20200311
Time               6.14
INSTRUM           spect
PROBHD    5 mm PABBO BB/
PULPROG            zg30
TD                65536
SOLVENT            DMSO
NS                   32
DS                    2
SWH            8012.820 Hz
FIDRES         0.122266 Hz
AQ            4.0894465 sec
RG               202.37
DW               62.400 usec
DE                 6.50 usec
TE                298.1 K
D1           1.00000000 sec
TD0                   1

======== CHANNEL f1 ========
SFO1        400.1924713 MHz
NUC1                 1H
P1                15.00 usec
PLW1        10.39999962 W

F2 - Processing parameters
SI                65536
SF          400.1900000 MHz
WDW                  EM
SSB      0
LB                 0.30 Hz
GB       0
PC                 1.00
```

Majed ElWard_H_Sor105

Microanalytical Unit - FOPCU - NMR laboratory
www.pharma.cu.edu.eg dir-mau.fopcu@pharma.cu.edu.eg

BRUKER

```
Current Data Parameters
NAME      Majed ElWard_H_Sor105
EXPNO                  10
PROCNO                  1

F2 - Acquisition Parameters
Date_            20200311
Time                 6.33
INSTRUM             spect
PROBHD   5 mm PABBO BB/
PULPROG              zg30
TD                  65536
SOLVENT              DMSO
NS                     32
DS                      2
SWH              8012.820 Hz
FIDRES           0.122266 Hz
AQ              4.0894465 sec
RG                  180.8
DW                 62.400 usec
DE                   6.50 usec
TE                  298.1 K
D1             1.00000000 sec
TD0                     1

======== CHANNEL f1 ========
SFO1          400.1924713 MHz
NUC1                   1H
P1                  15.00 usec
PLW1          10.39999962 W

F2 - Processing parameters
SI                  65536
SF            400.1900000 MHz
WDW                    EM
SSB      0
LB                   0.30 Hz
GB       0
PC                   1.00
```

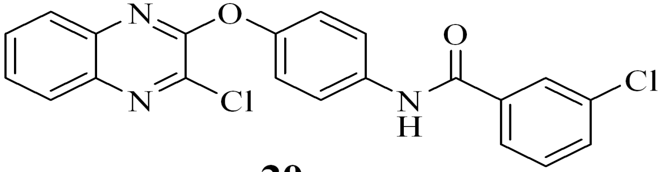

20c

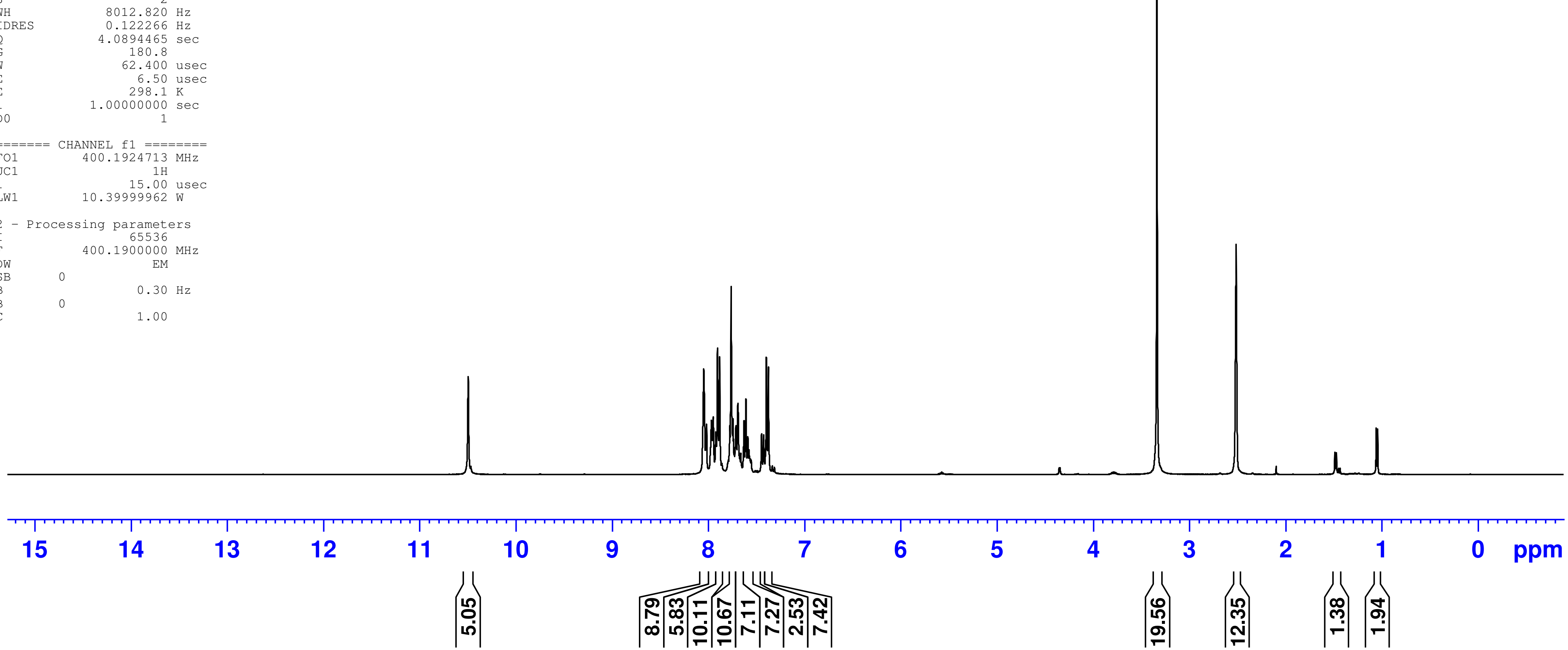

Majed ElWard_H_Sor107

Microanalytical Unit - FOPCU - NMR laboratory
www.pharma.cu.edu.eg dir-mau.fopcu@pharma.cu.edu.eg

```
Current Data Parameters
NAME      Majed ElWard_H_Sor107
EXPNO                     10
PROCNO                     1

F2 - Acquisition Parameters
Date_               20200311
Time                    6.45
INSTRUM                spect
PROBHD   5 mm PABBO BB/
PULPROG                 zg30
TD                     65536
SOLVENT                 DMSO
NS                        32
DS                         2
SWH                 8012.820 Hz
FIDRES              0.122266 Hz
AQ                 4.0894465 sec
RG                     180.8
DW                    62.400 usec
DE                      6.50 usec
TE                     298.0 K
D1                1.00000000 sec
TD0                        1

======== CHANNEL f1 ========
SFO1             400.1924713 MHz
NUC1                      1H
P1                     15.00 usec
PLW1             10.39999962 W

F2 - Processing parameters
SI                     65536
SF               400.1900000 MHz
WDW                       EM
SSB      0
LB                      0.30 Hz
GB       0
PC                      1.00
```

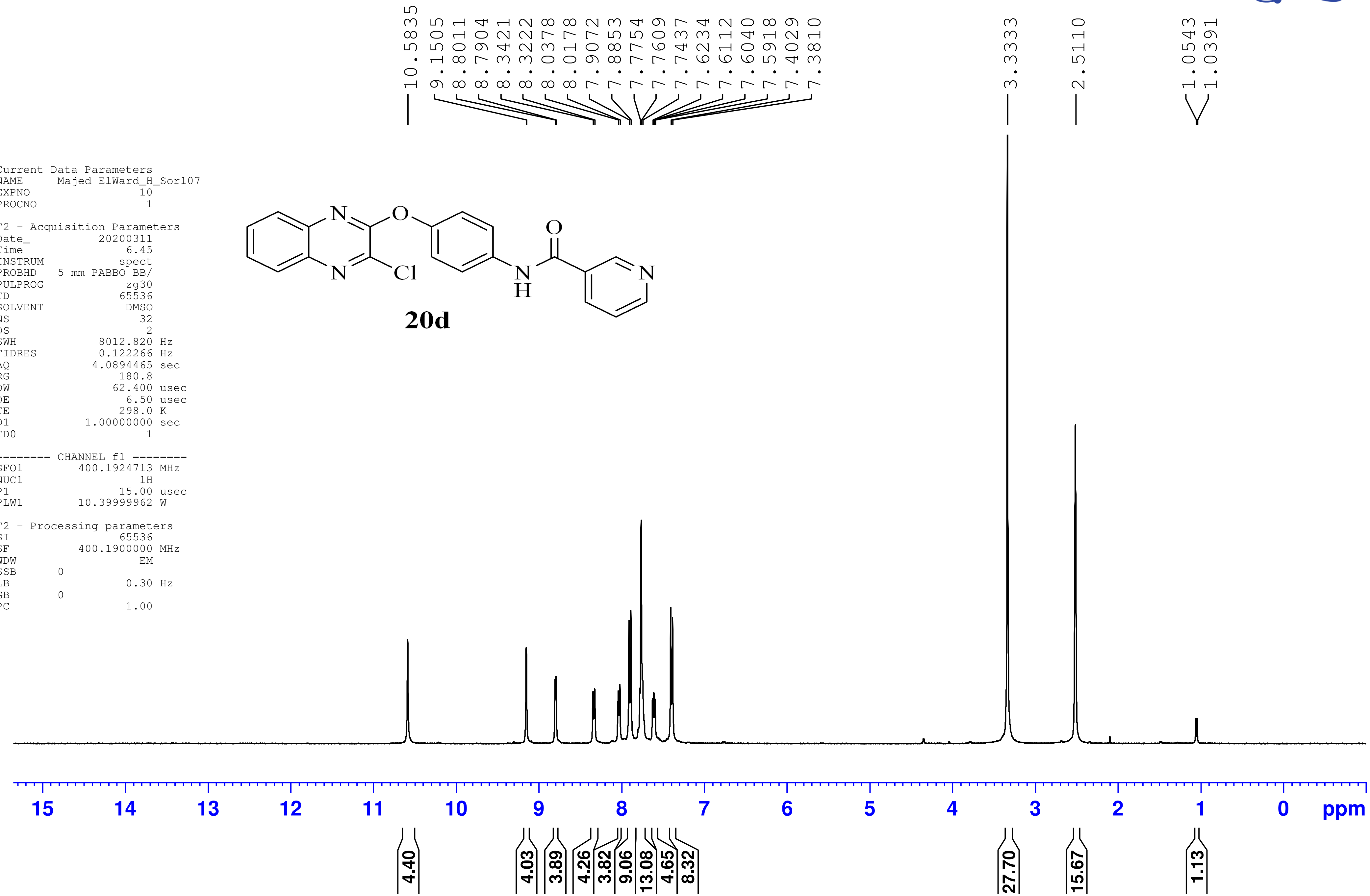

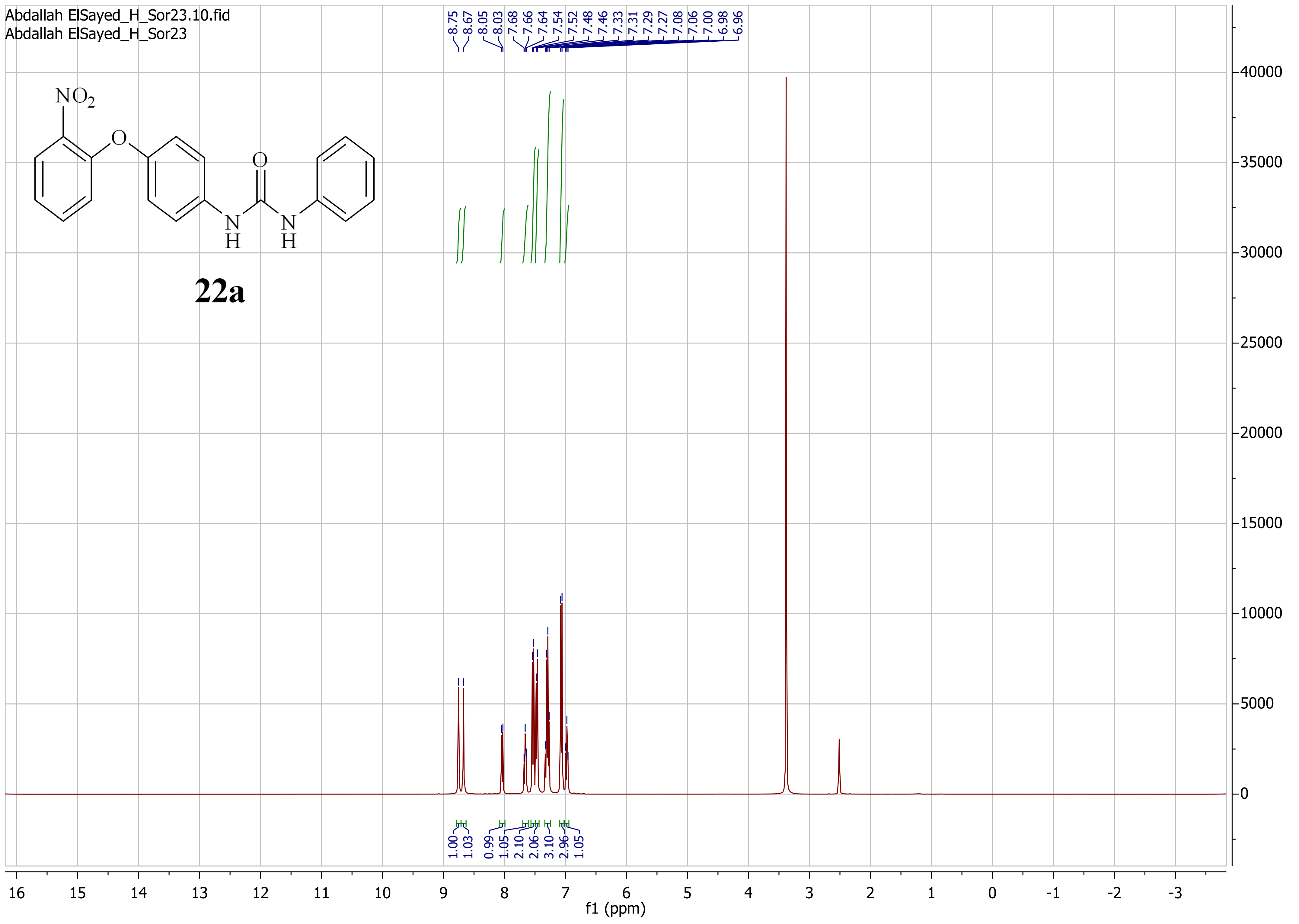
Abdallah ElSayed_H_Sor23.10.fid
Abdallah ElSayed_H_Sor23
8.75
8.67
8.05
8.03
7.68
7.66
7.64
7.54
7.52
7.48
7.46
7.33
7.31
7.29
7.27
7.08
7.06
7.00
6.98
6.96
NO2
O
O
N
H
N
H
22a
1.00
1.03
0.99
1.05
2.10
2.06
3.10
2.96
1.05
f1 (ppm)

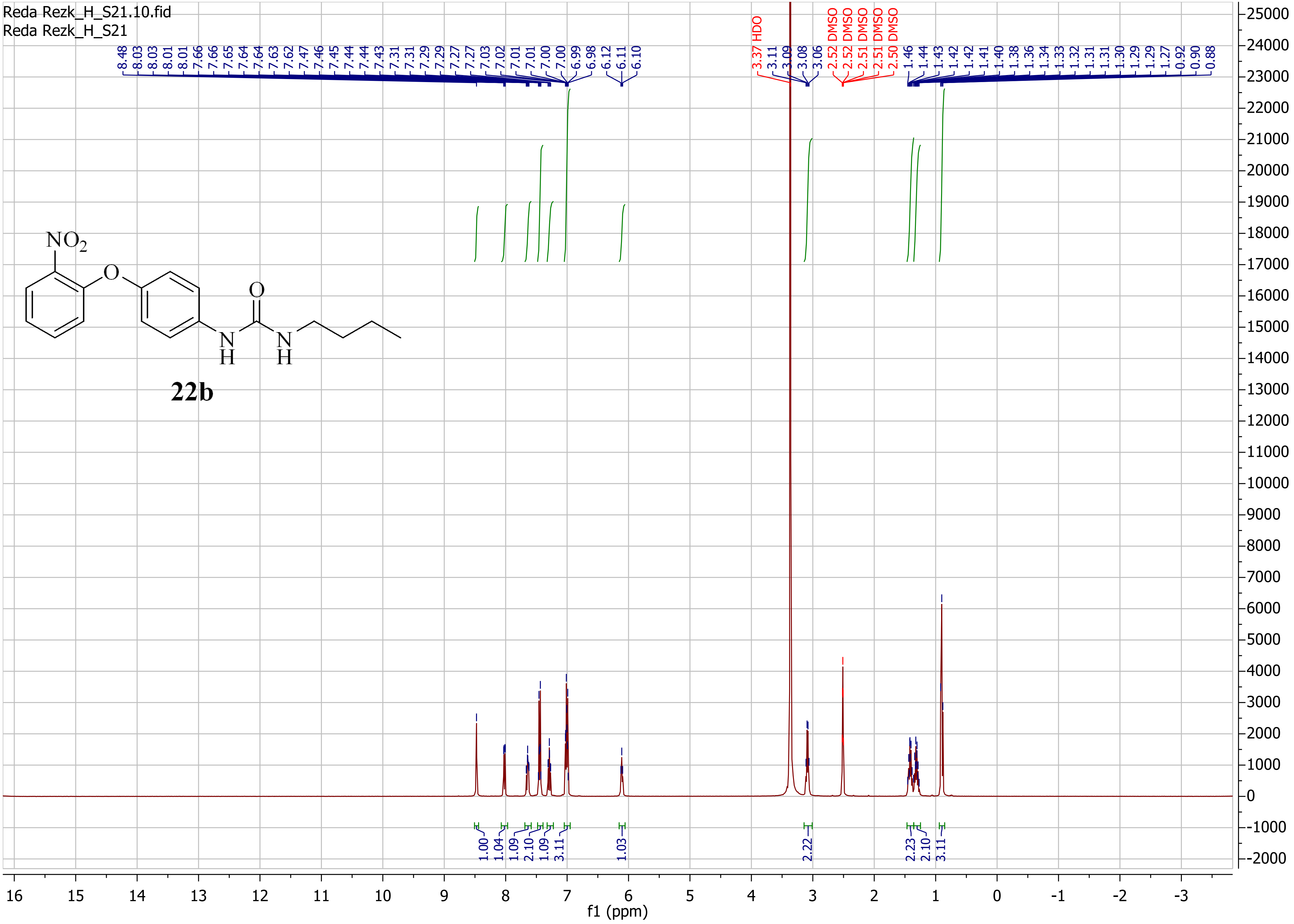
Reda Rezk_H_S21.10.fid
Reda Rezk_H_S21
8.48 8.03 8.03 8.01 8.01 7.66 7.66 7.65 7.64 7.64 7.63 7.62 7.47 7.46 7.45 7.44 7.44 7.43 7.31 7.31 7.29 7.29 7.27 7.27 7.03 7.02 7.01 7.01 7.00 7.00 6.99 6.98 6.12 6.11 6.10
3.37 HDO
3.11 3.09 3.08 3.06
2.52 DMSO 2.52 DMSO 2.51 DMSO 2.51 DMSO 2.50 DMSO
1.46 1.44 1.43 1.42 1.42 1.41 1.40 1.38 1.36 1.34 1.33 1.32 1.31 1.31 1.30 1.29 1.29 1.27 0.92 0.90 0.88
NO_2
O
O
N
H
N
H
1.00 1.04 1.09 2.10 1.09 3.11 1.03 2.22 2.23 2.10 3.11
f1 (ppm)

22b

Abdallah ElSayed_H_Sor29.10.fid
Abdallah ElSayed_H_Sor29

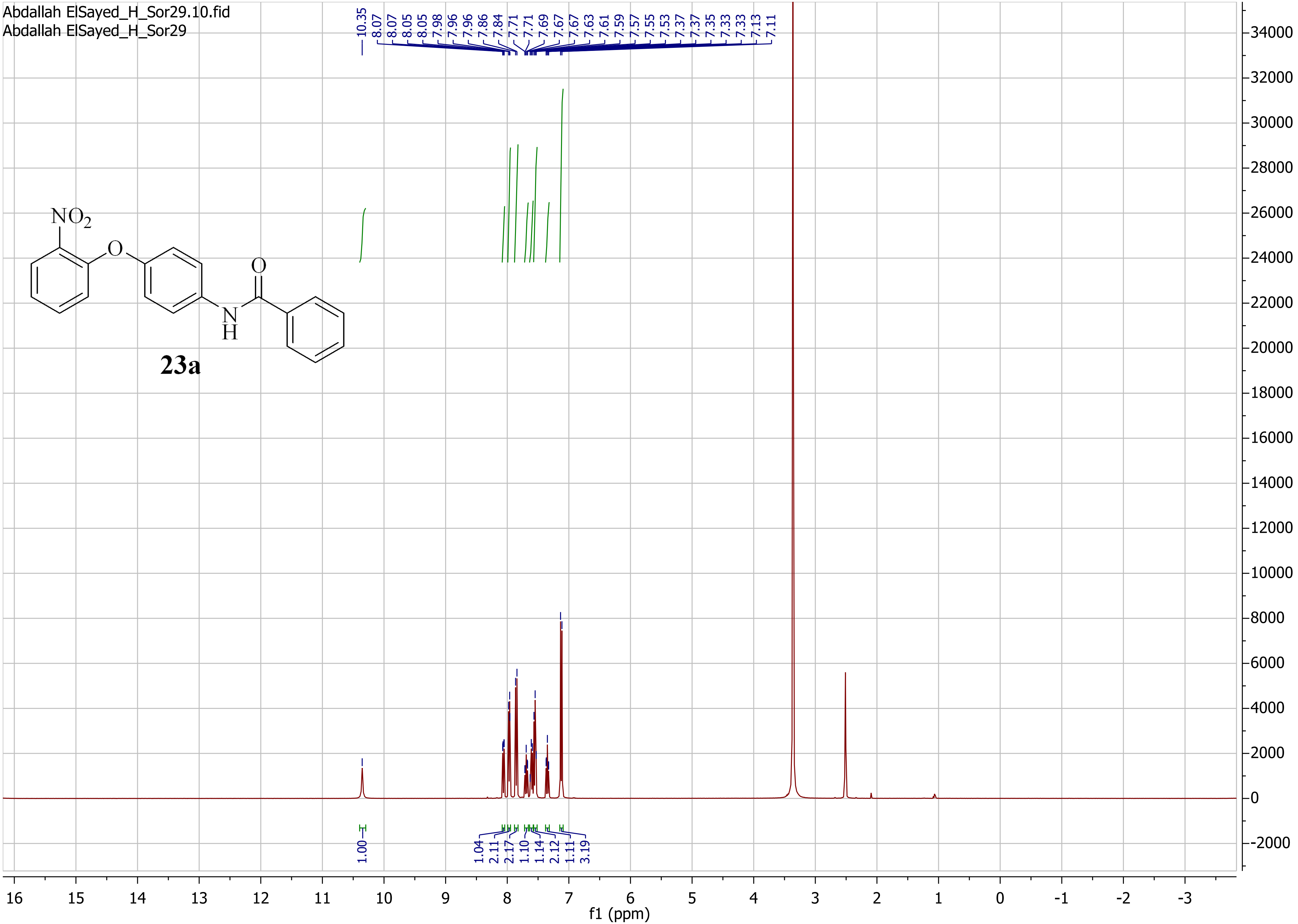

23a

Majed ElWard_H_Sor33

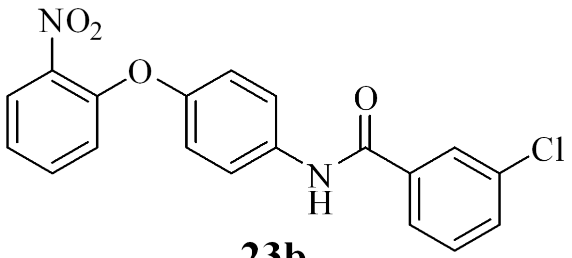

23b

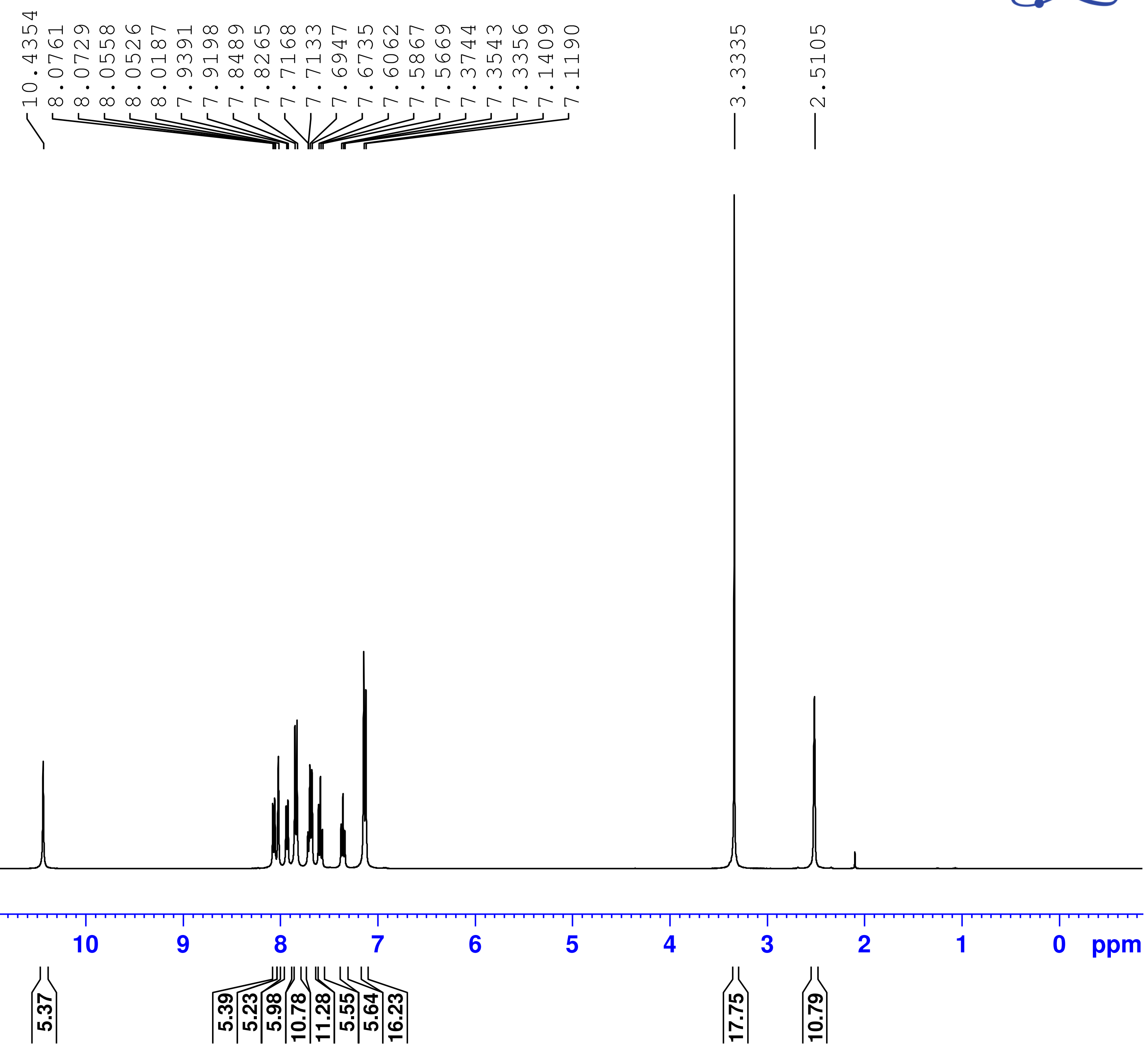


```
Current Data Parameters
NAME      Majed ElWard_H_Sor33
EXPNO                   10
PROCNO                   1

F2 - Acquisition Parameters
Date_           20200311
Time                6.27
INSTRUM            spect
PROBHD   5 mm PABBO BB/
PULPROG             zg30
TD                 65536
SOLVENT             DMSO
NS                    32
DS                     2
SWH             8012.820 Hz
FIDRES          0.122266 Hz
AQ             4.0894465 sec
RG                 180.8
DW                62.400 usec
DE                  6.50 usec
TE                 298.0 K
D1            1.00000000 sec
TD0                    1

======== CHANNEL f1 ========
SFO1         400.1924713 MHz
NUC1                  1H
P1                 15.00 usec
PLW1         10.39999962 W

F2 - Processing parameters
SI                 65536
SF           400.1900000 MHz
WDW                   EM
SSB       0
LB                  0.30 Hz
GB        0
PC                  1.00
```

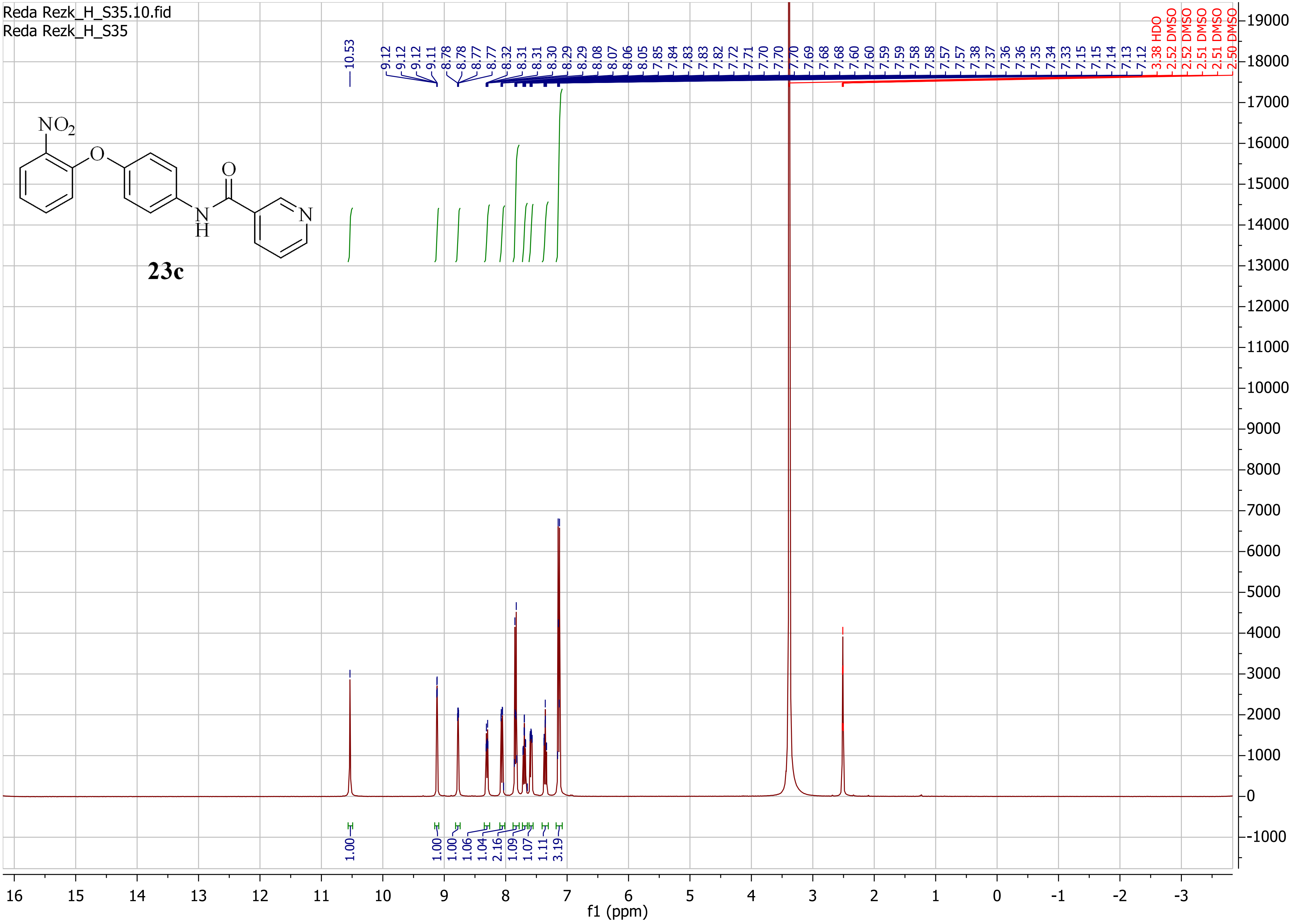
Reda Rezk_H_S35.10.fid
Reda Rezk_H_S35
NO2
O
O
N
H
N
23c
10.53
9.12 9.12 9.12 9.11 8.78 8.78 8.77 8.77 8.32 8.31 8.31 8.30 8.29 8.29 8.08 8.07 8.06 8.05 7.85 7.84 7.83 7.83 7.82 7.72 7.71 7.70 7.70 7.70 7.69 7.68 7.68 7.60 7.60 7.59 7.59 7.58 7.58 7.57 7.57 7.38 7.37 7.36 7.36 7.35 7.34 7.33 7.15 7.15 7.14 7.13 7.12
3.38 HDO
2.52 DMSO
2.52 DMSO
2.51 DMSO
2.51 DMSO
2.50 DMSO
1.00
1.00 1.00 1.06 1.04 2.16 1.09 1.07 1.11 3.19
f1 (ppm)

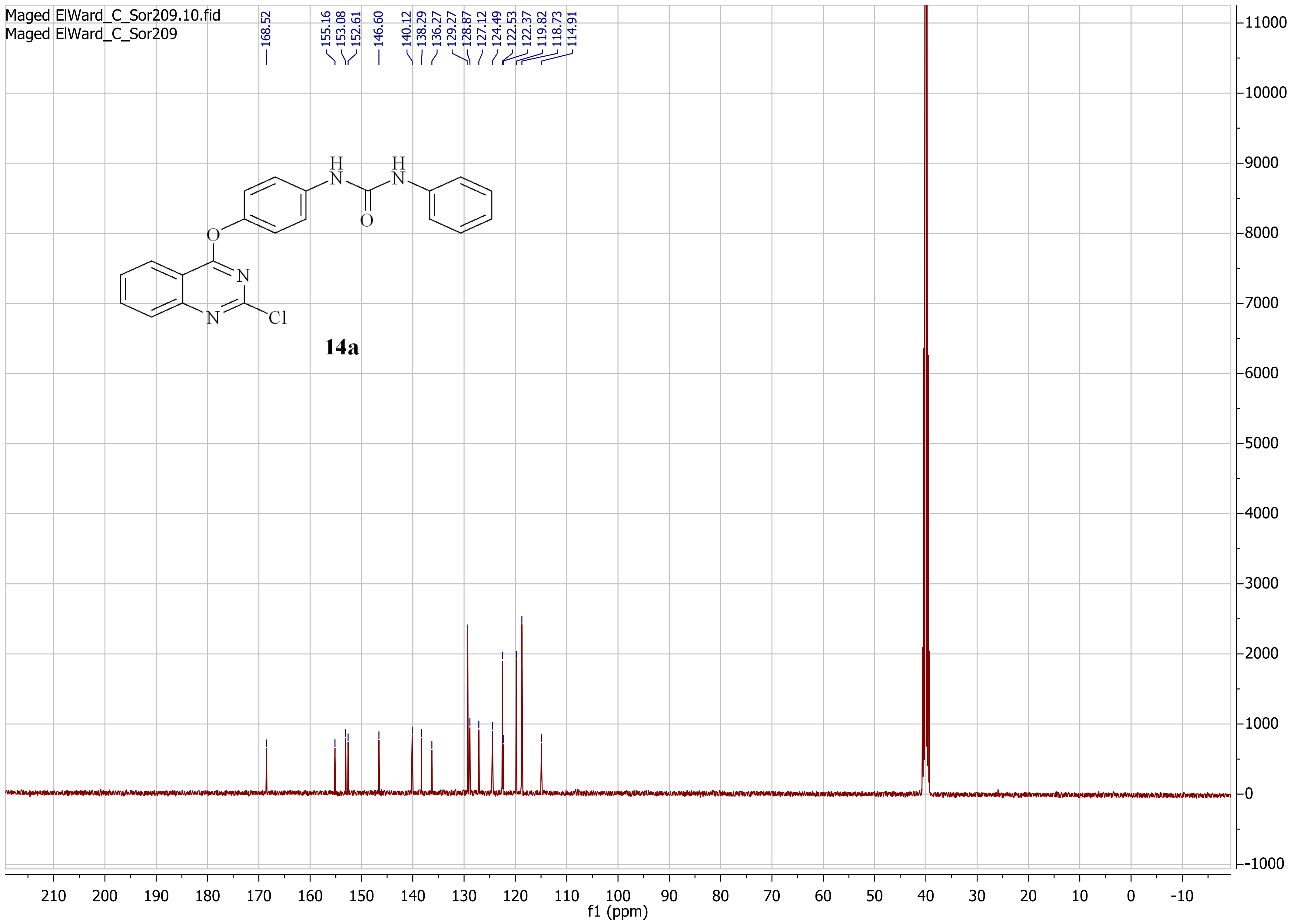
Maged ElWard_C_Sor209.10.fid
Maged ElWard_C_Sor209
168.52
155.16
153.08
152.61
146.60
140.12
138.29
136.27
129.27
128.87
127.12
124.49
122.53
122.37
119.82
118.73
114.91
14a
f1 (ppm)

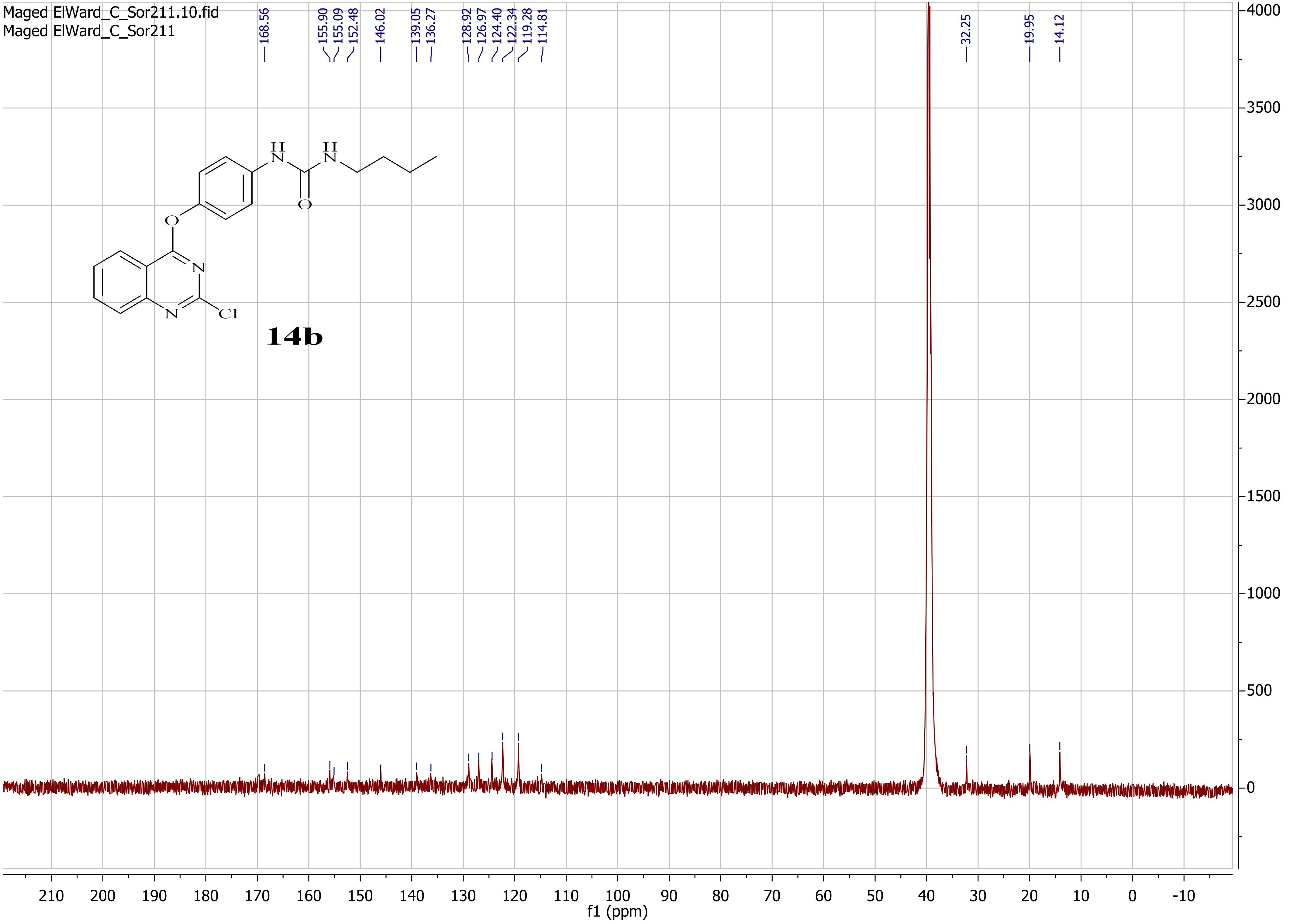
Maged ElWard_C_Sor211.10.fid
Maged ElWard_C_Sor211
168.56
155.90
155.09
152.48
146.02
139.05
136.27
128.92
126.97
124.40
122.34
119.28
114.81
32.25
19.95
14.12
H
N
H
N
O
O
N
N
Cl
14b
f1 (ppm)
210 200 190 180 170 160 150 140 130 120 110 100 90 80 70 60 50 40 30 20 10 0 -10
4000 3500 3000 2500 2000 1500 1000 500 0

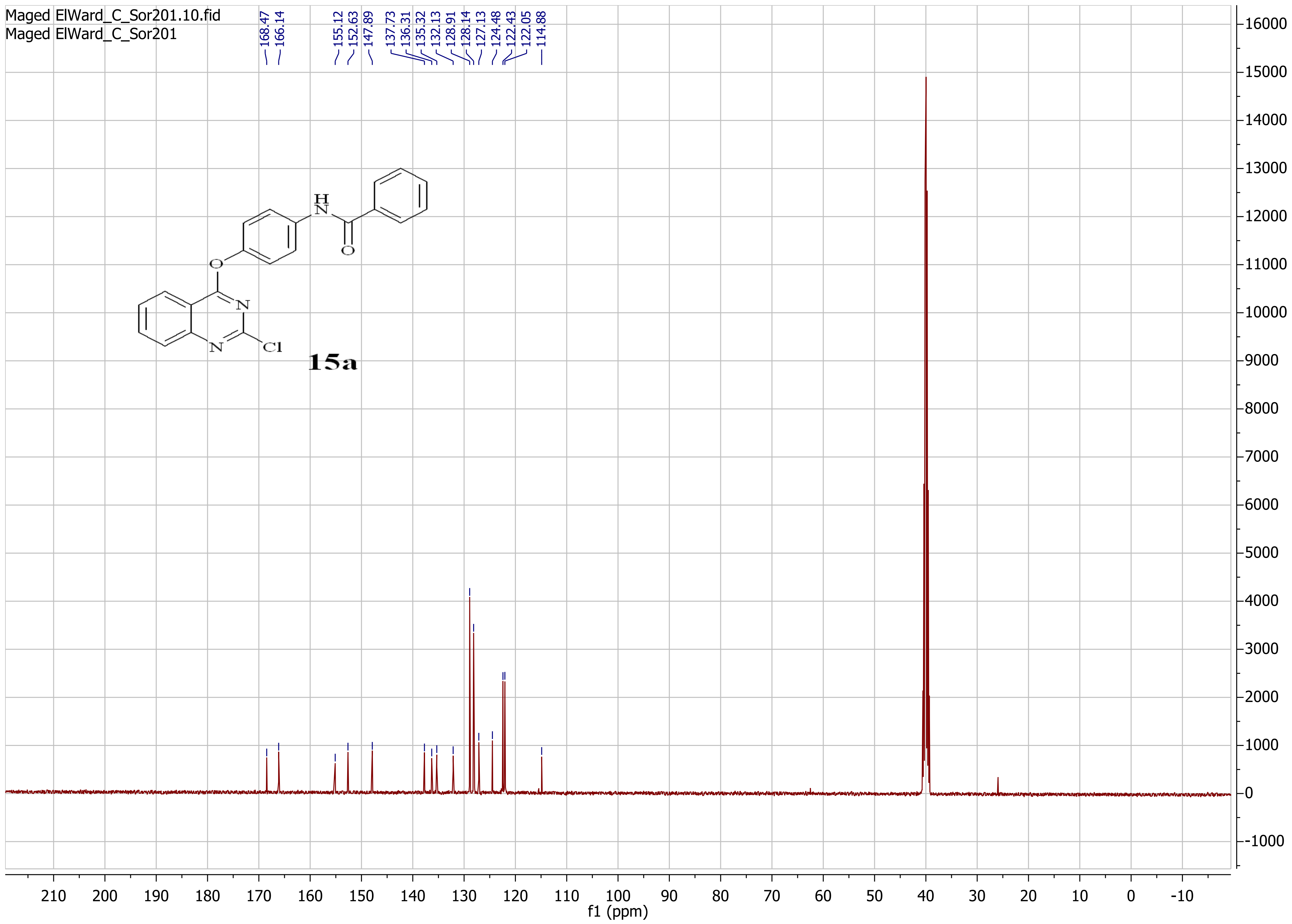
Maged ElWard_C_Sor201.10.fid
Maged ElWard_C_Sor201
168.47
166.14
155.12
152.63
147.89
137.73
136.31
135.32
132.13
128.91
128.14
127.13
124.48
122.43
122.05
114.88
15a
f1 (ppm)

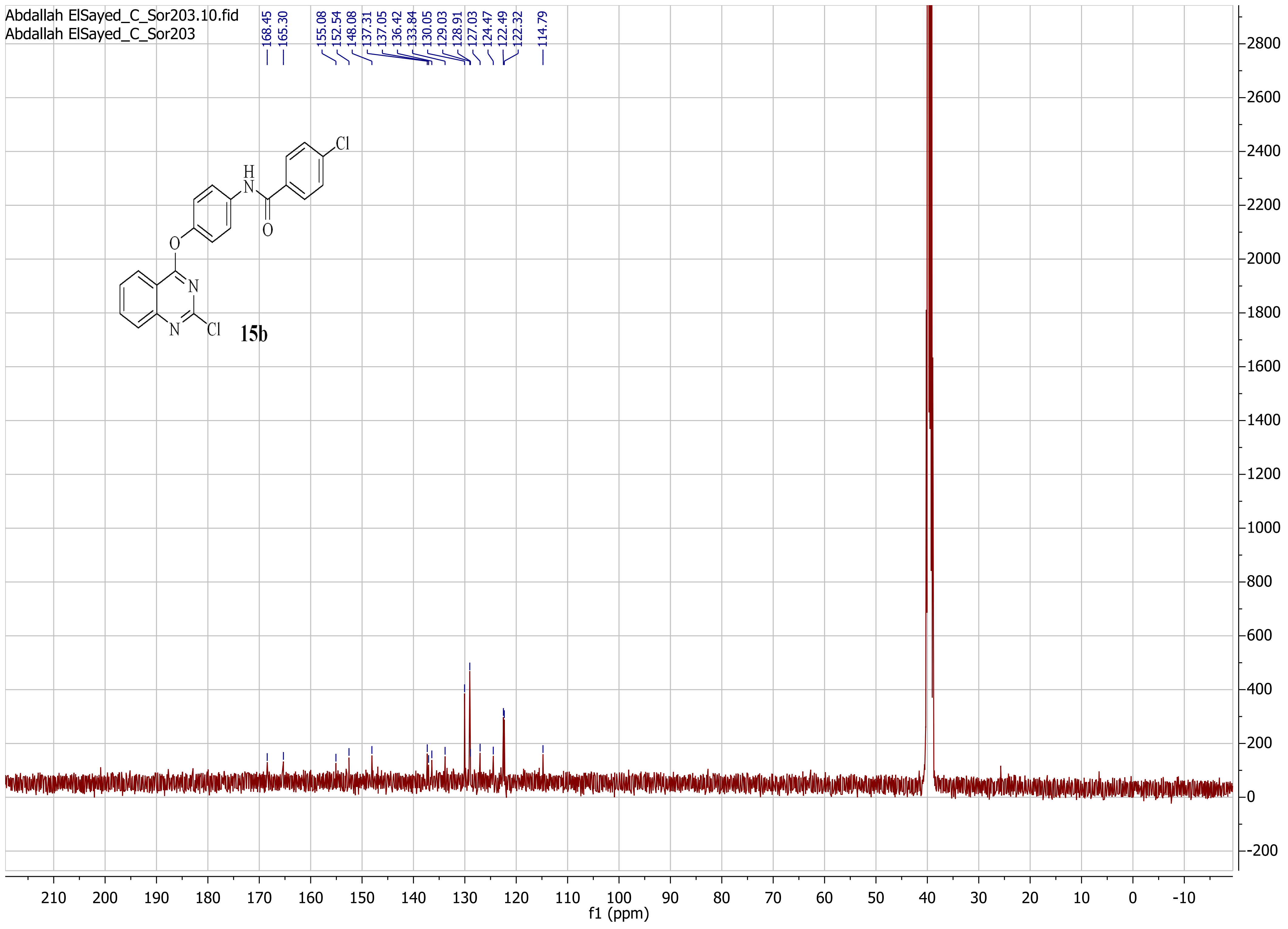

Abdallah ElSayed_C_Sor203.10.fid
Abdallah ElSayed_C_Sor203
168.45
165.30
155.08
152.54
148.08
137.31
137.05
136.42
133.84
130.05
129.03
128.91
127.03
124.47
122.49
122.32
114.79
Cl
H
N
O
O
N
N
Cl
15b
f1 (ppm)

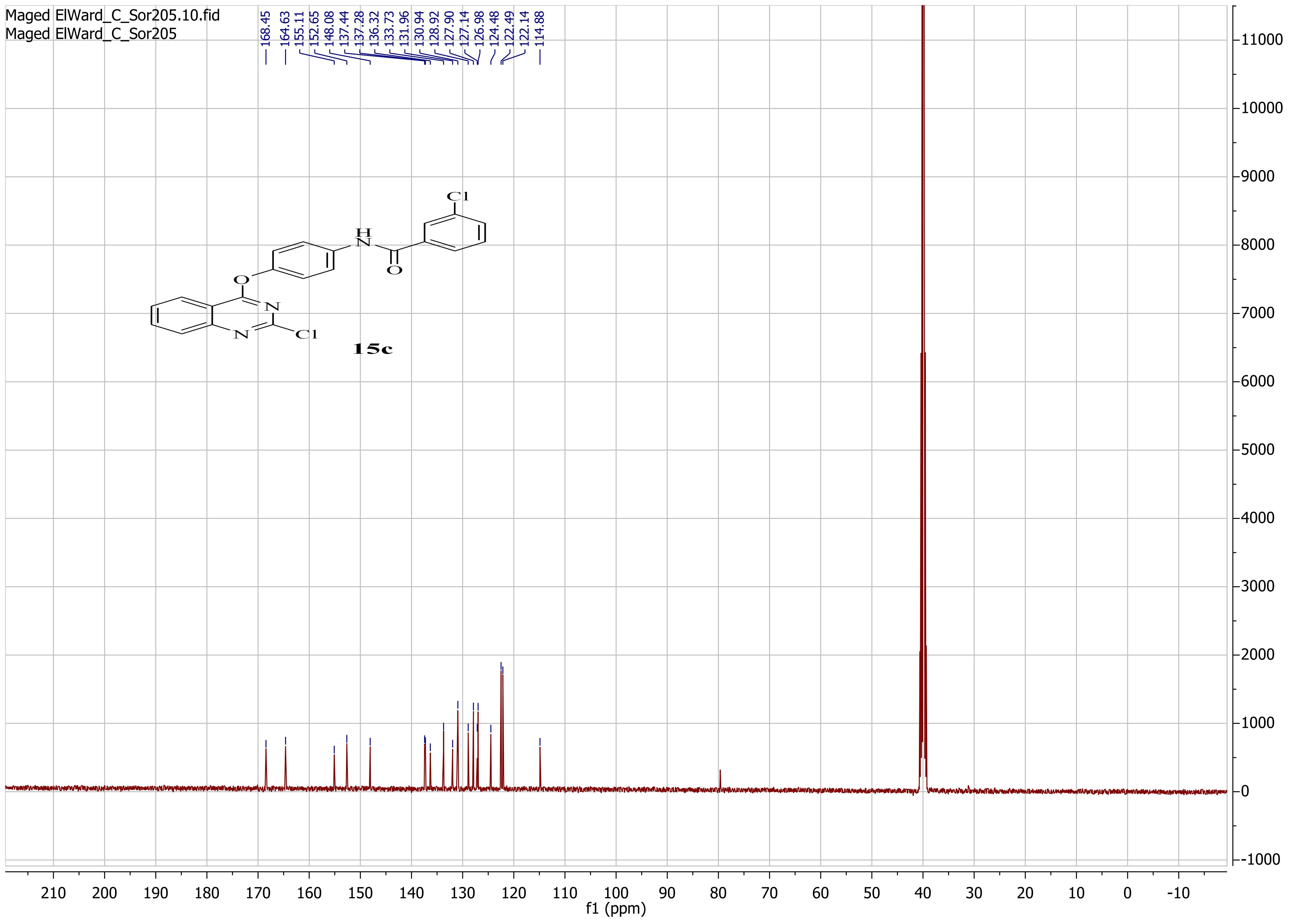

Maged ElWard_C_Sor205.10.fid
Maged ElWard_C_Sor205
168.45
164.63
155.11
152.65
148.08
137.44
137.28
136.32
133.73
131.96
130.94
128.92
127.90
127.14
126.98
124.48
122.49
122.14
114.88
Cl
H
N
O
O
N
N
Cl
15c
11000
10000
9000
8000
7000
6000
5000
4000
3000
2000
1000
0
-1000
210
200
190
180
170
160
150
140
130
120
110
100
90
80
70
60
50
40
30
20
10
0
-10
f1 (ppm)

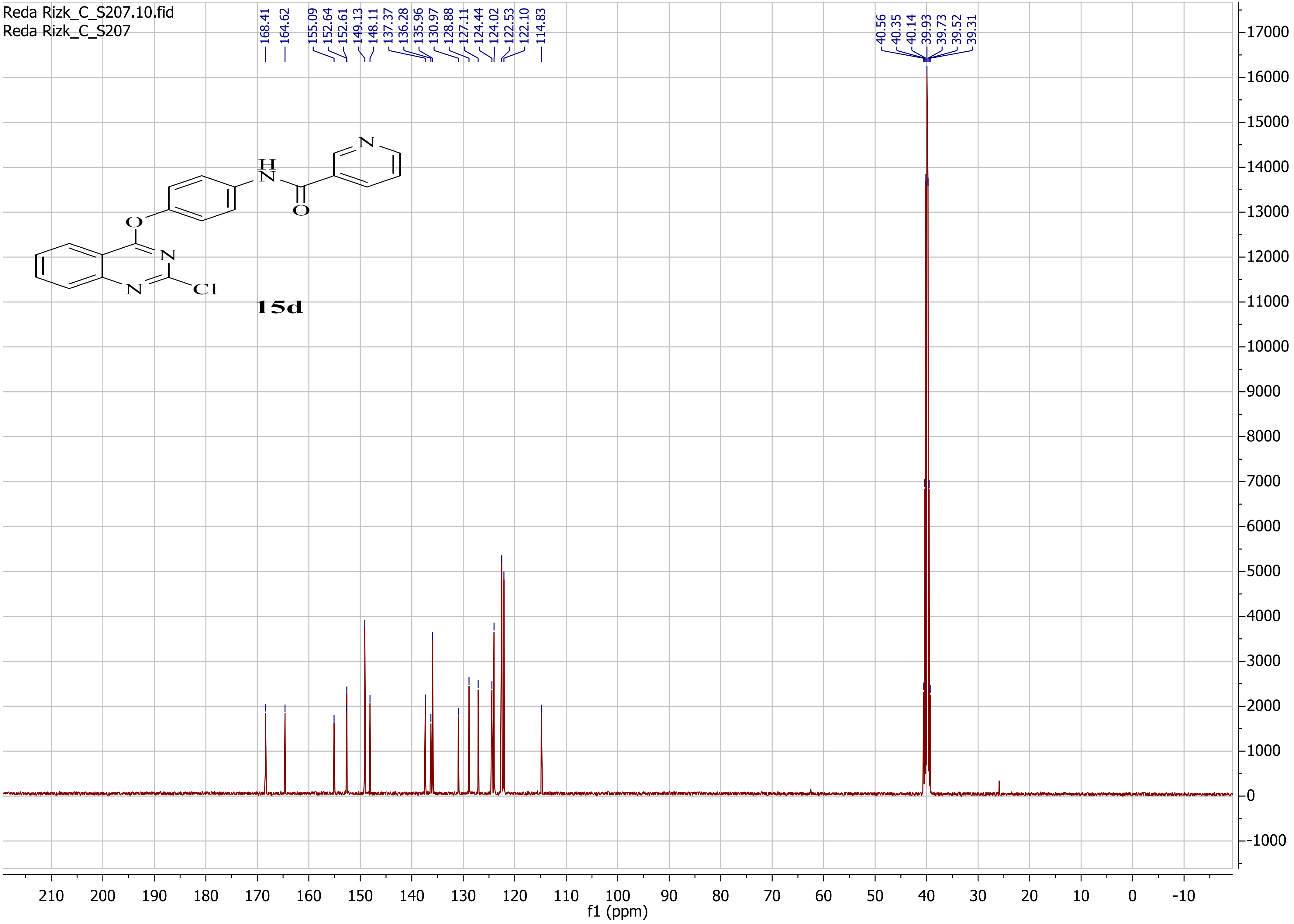
Reda Rizk_C_S207.10.fid
Reda Rizk_C_S207
168.41
164.62
155.09
152.64
152.61
149.13
148.11
137.37
136.28
135.96
130.97
128.88
127.11
124.44
124.02
122.53
122.10
114.83
40.56
40.35
40.14
39.93
39.73
39.52
39.31
15d
f1 (ppm)

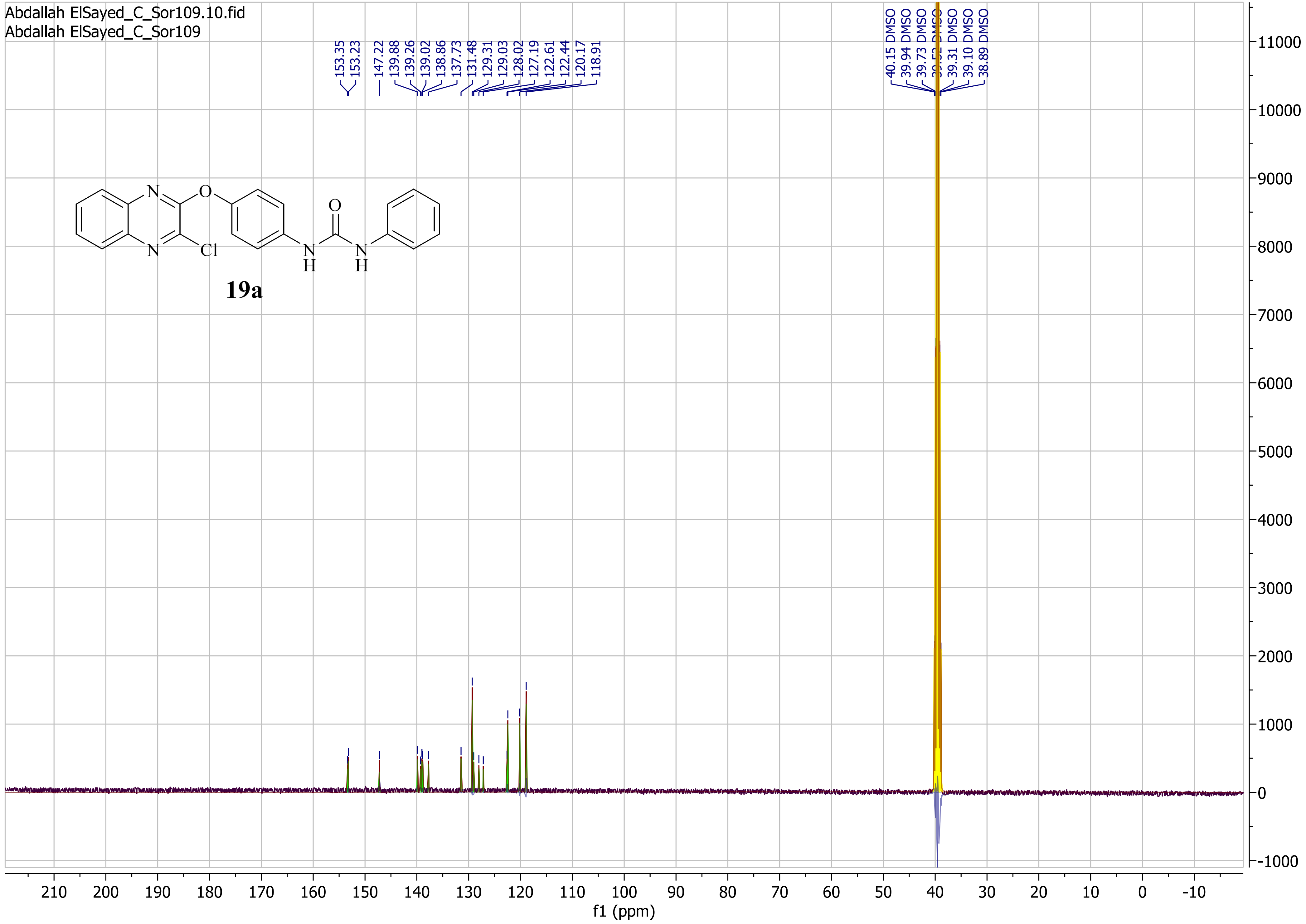

Abdallah ElSayed_C_Sor109.10.fid
Abdallah ElSayed_C_Sor109
153.35
153.23
147.22
139.88
139.26
139.02
138.86
137.73
131.48
129.31
129.03
128.02
127.19
122.61
122.44
120.17
118.91
40.15 DMSO
39.94 DMSO
39.73 DMSO
39.31 DMSO
39.10 DMSO
38.89 DMSO
N
O
Cl
H
19a
11000
10000
9000
8000
7000
6000
5000
4000
3000
2000
1000
0
-1000
210
200
190
180
170
160
150
140
130
120
110
100
90
80
70
60
50
40
30
20
10
0
-10
f1 (ppm)

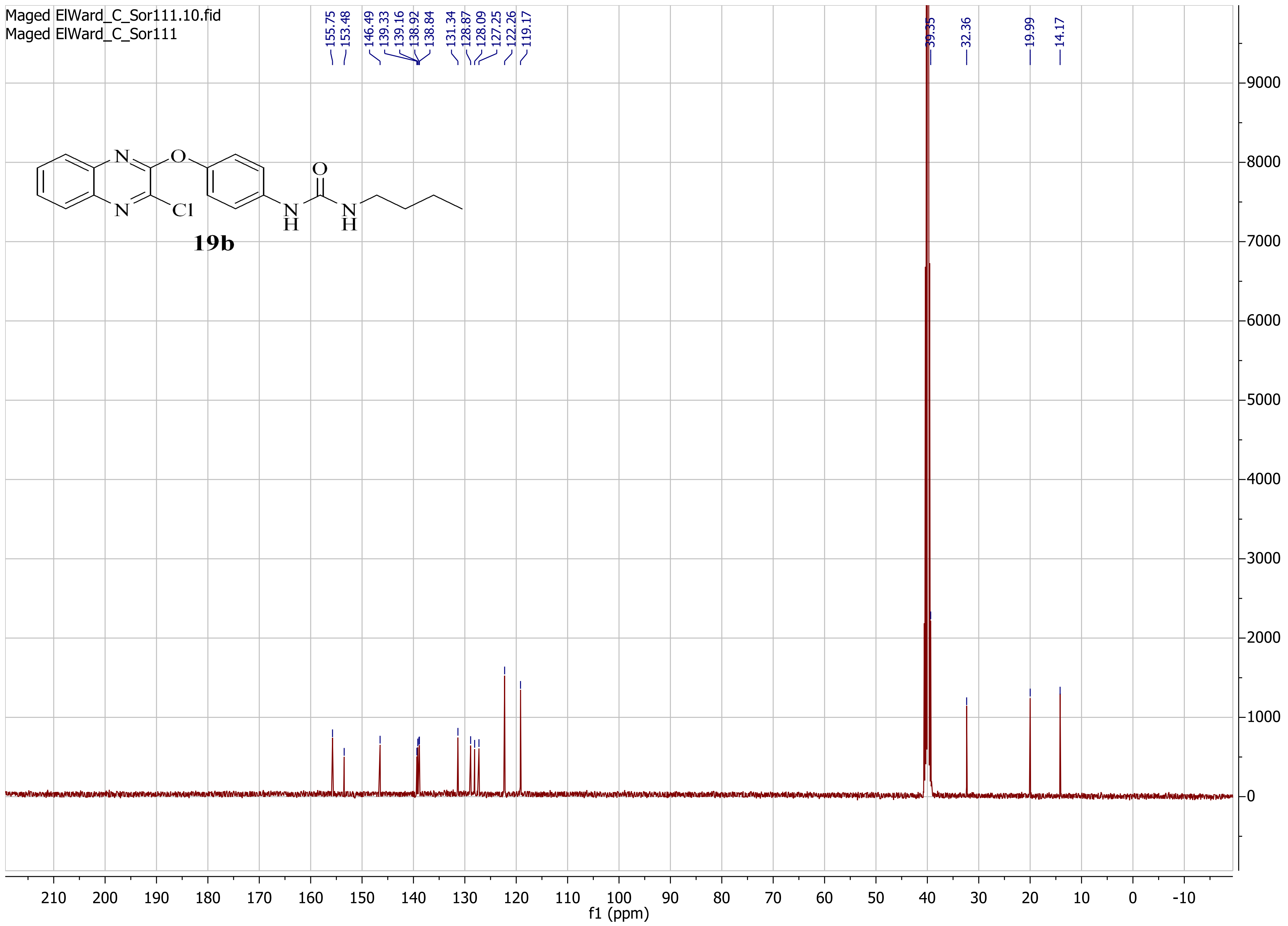
Maged ElWard_C_Sor111.10.fid
Maged ElWard_C_Sor111
155.75
153.48
146.49
139.33
139.16
138.92
138.84
131.34
128.87
128.09
127.25
122.26
119.17
39.35
32.36
19.99
14.17
N
O
N
Cl
O
N
H
N
H
19b
f1 (ppm)

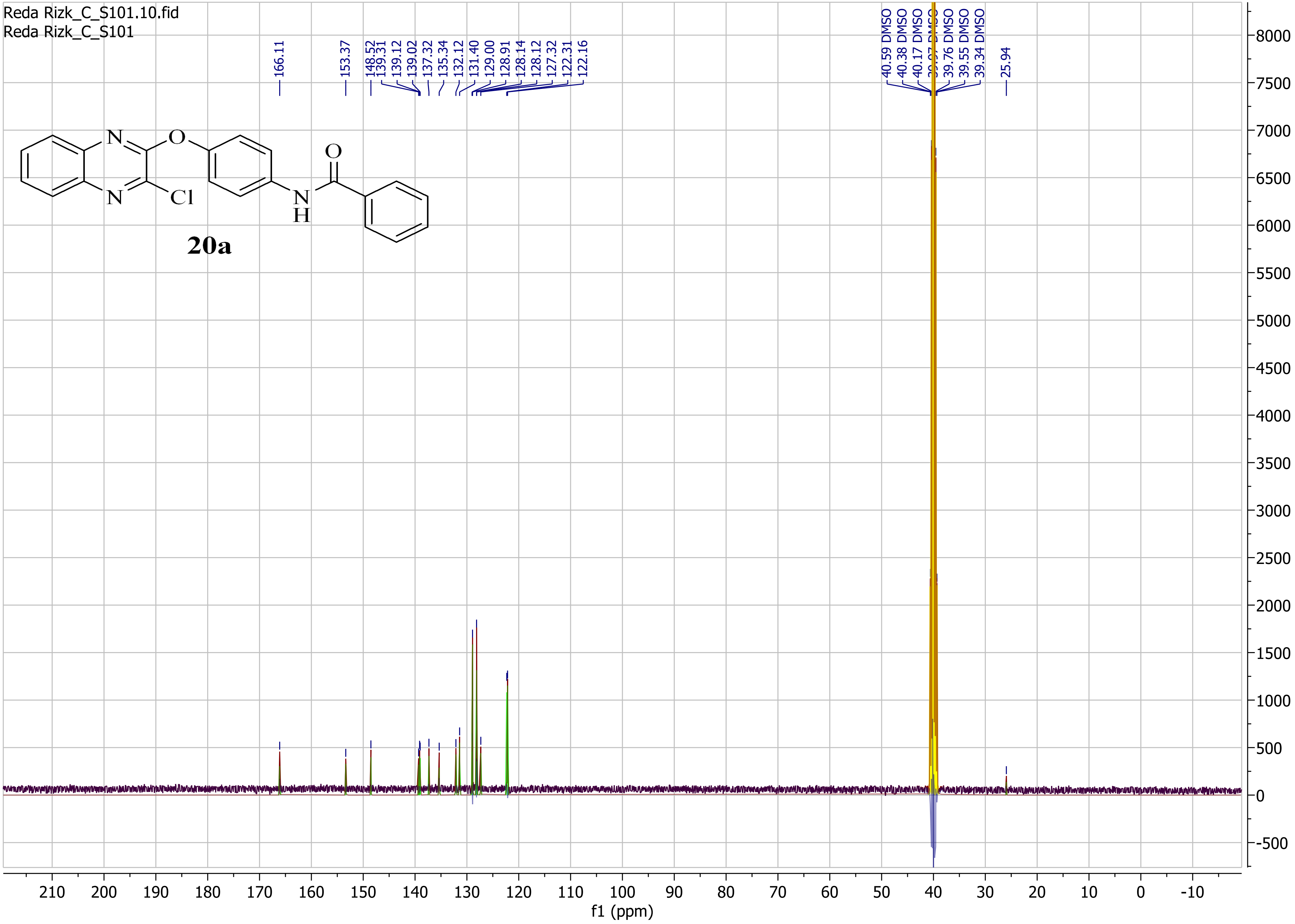

Reda Rizk_C_S101.10.fid
Reda Rizk_C_S101
166.11
153.37
148.52
139.31
139.12
139.02
137.32
135.34
132.12
131.40
129.00
128.91
128.14
128.12
127.32
122.31
122.16
40.59 DMSO
40.38 DMSO
40.17 DMSO
39.76 DMSO
39.55 DMSO
39.34 DMSO
25.94
20a
f1 (ppm)

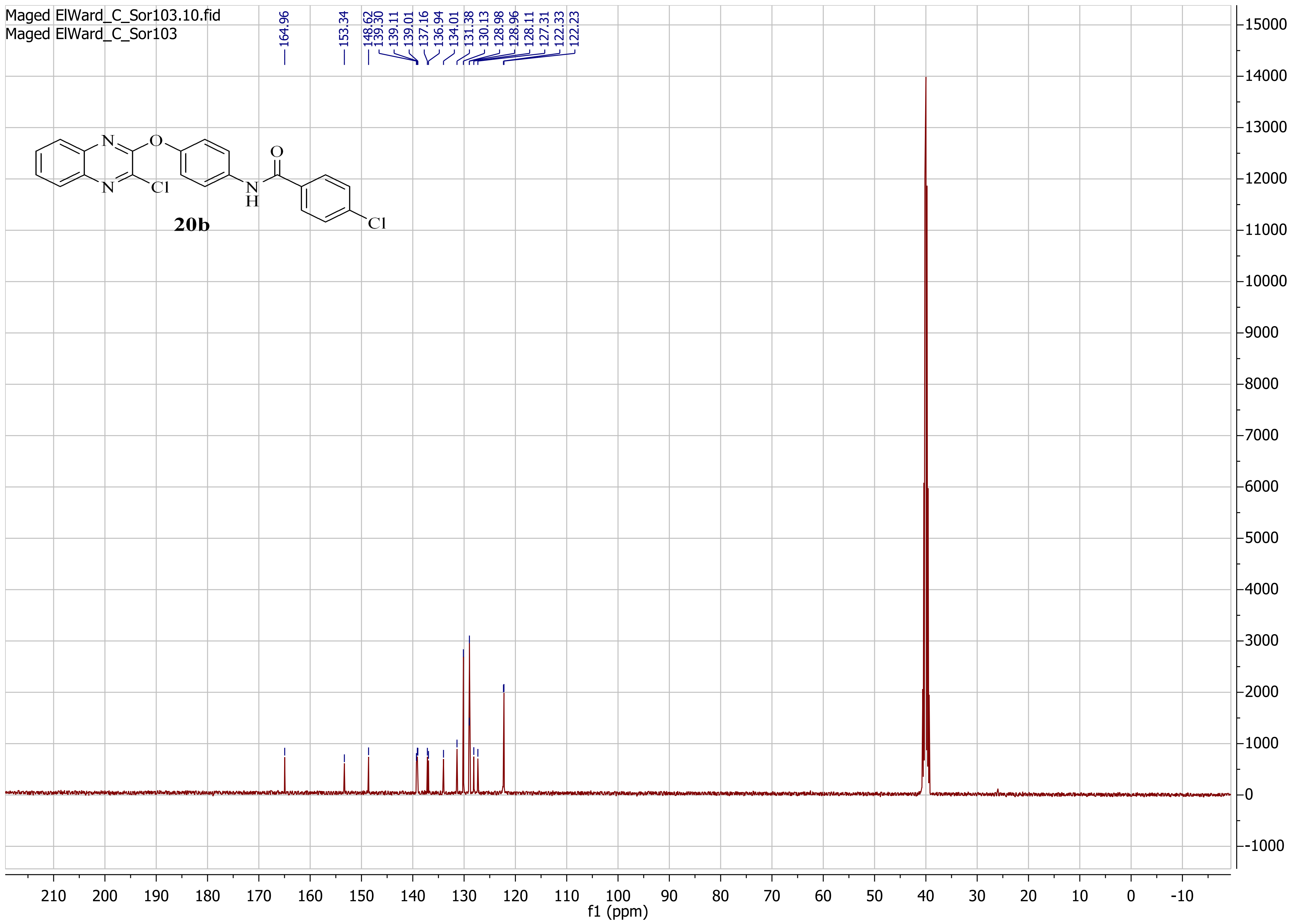
Maged ElWard_C_Sor103.10.fid
Maged ElWard_C_Sor103
164.96
153.34
148.62
139.30
139.11
139.01
137.16
136.94
134.01
131.38
130.13
128.98
128.96
128.11
127.31
122.33
122.23
N
O
N
Cl
O
N
H
Cl
20b
f1 (ppm)

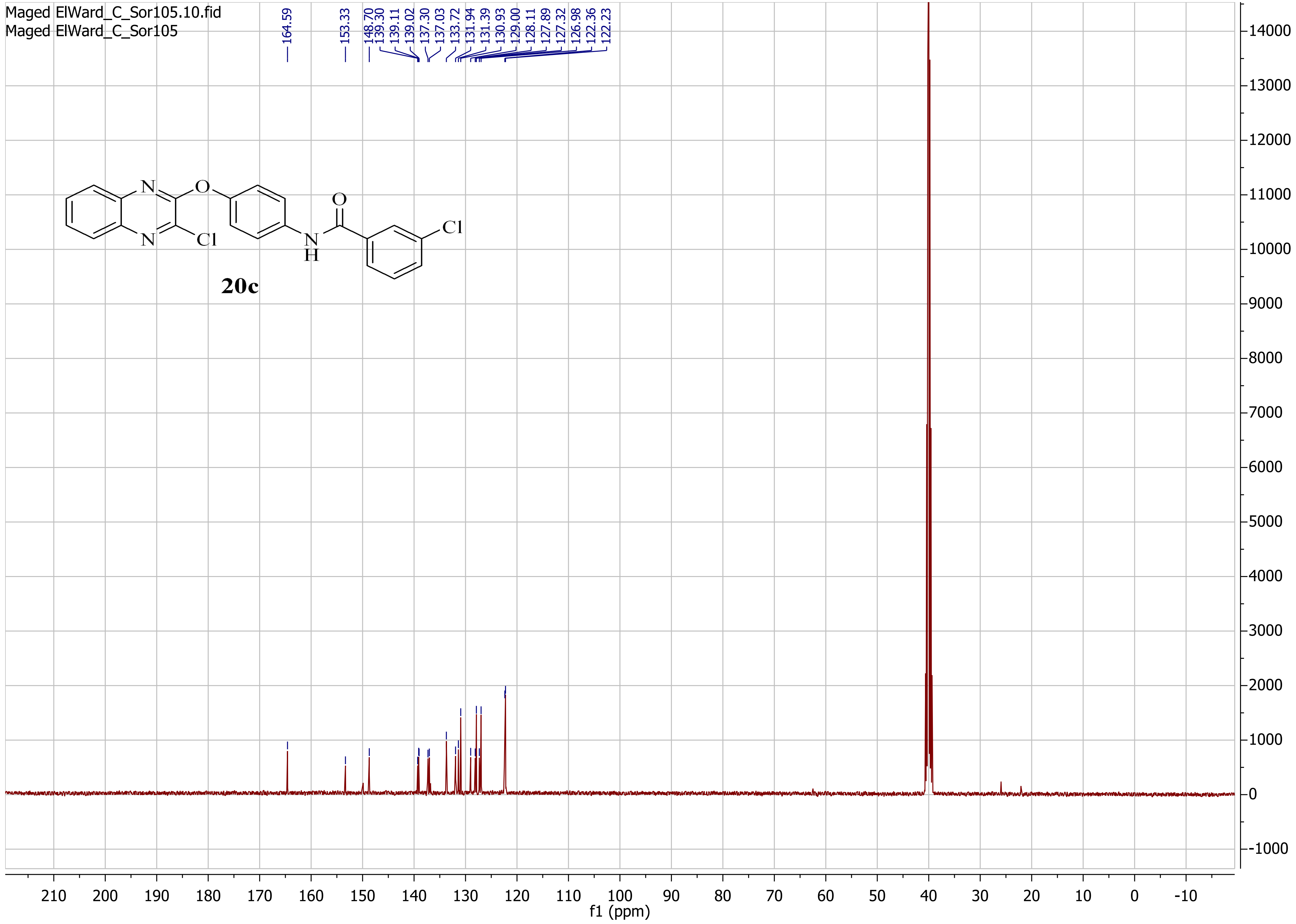

Maged ElWard_C_Sor105.10.fid
Maged ElWard_C_Sor105
164.59
153.33
148.70
139.30
139.11
139.02
137.30
137.03
133.72
131.94
131.39
130.93
129.00
128.11
127.89
127.32
126.98
122.36
122.23
N
O
N
Cl
O
N
H
Cl
20c
14000
13000
12000
11000
10000
9000
8000
7000
6000
5000
4000
3000
2000
1000
0
-1000
210 200 190 180 170 160 150 140 130 120 110 100 90 80 70 60 50 40 30 20 10 0 -10
f1 (ppm)

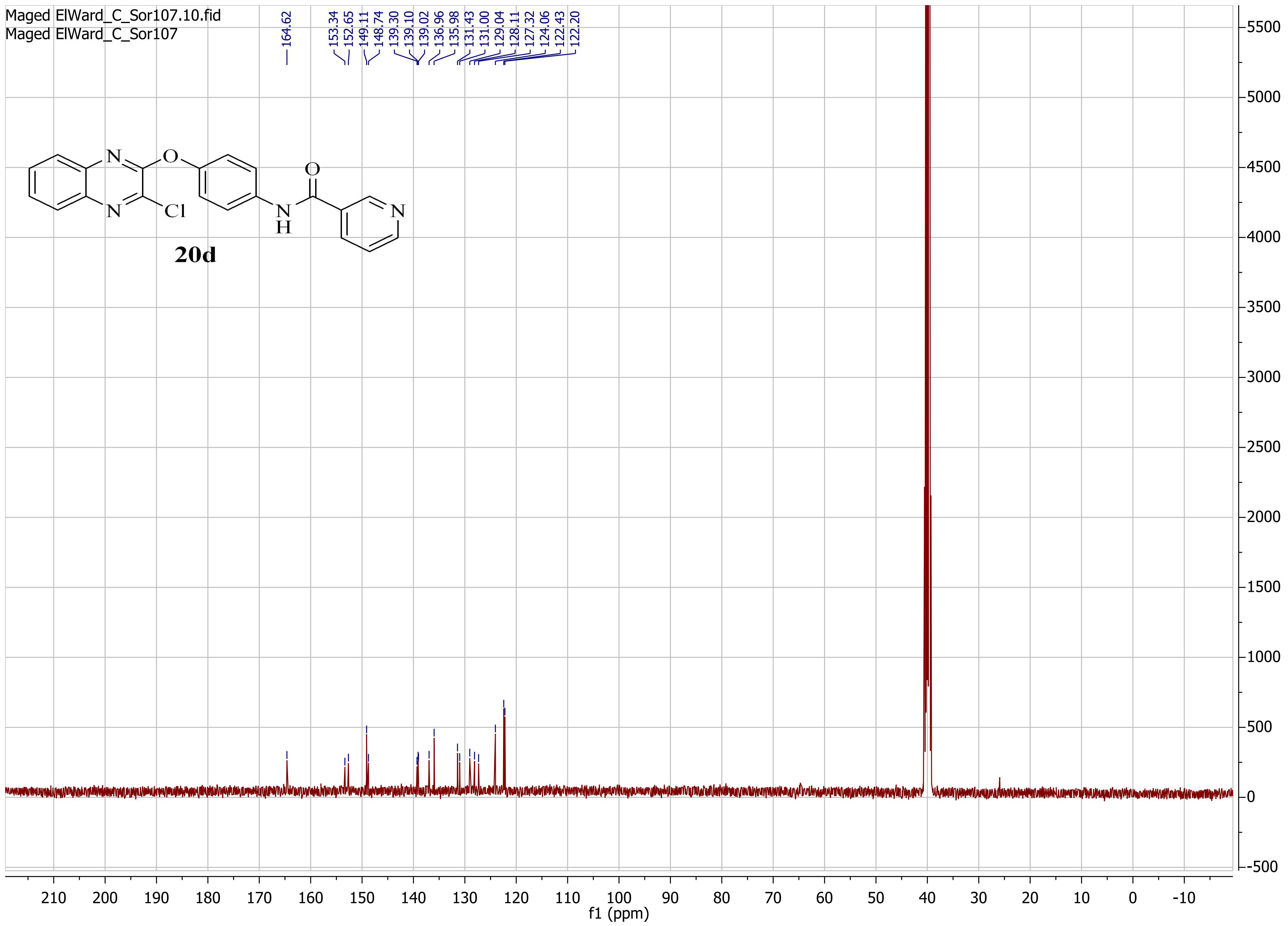
Maged ElWard_C_Sor107.10.fid
Maged ElWard_C_Sor107
164.62
153.34
152.65
149.11
148.74
139.30
139.10
139.02
136.96
135.98
131.43
131.00
129.04
128.11
127.32
124.06
122.43
122.20
20d
f1 (ppm)

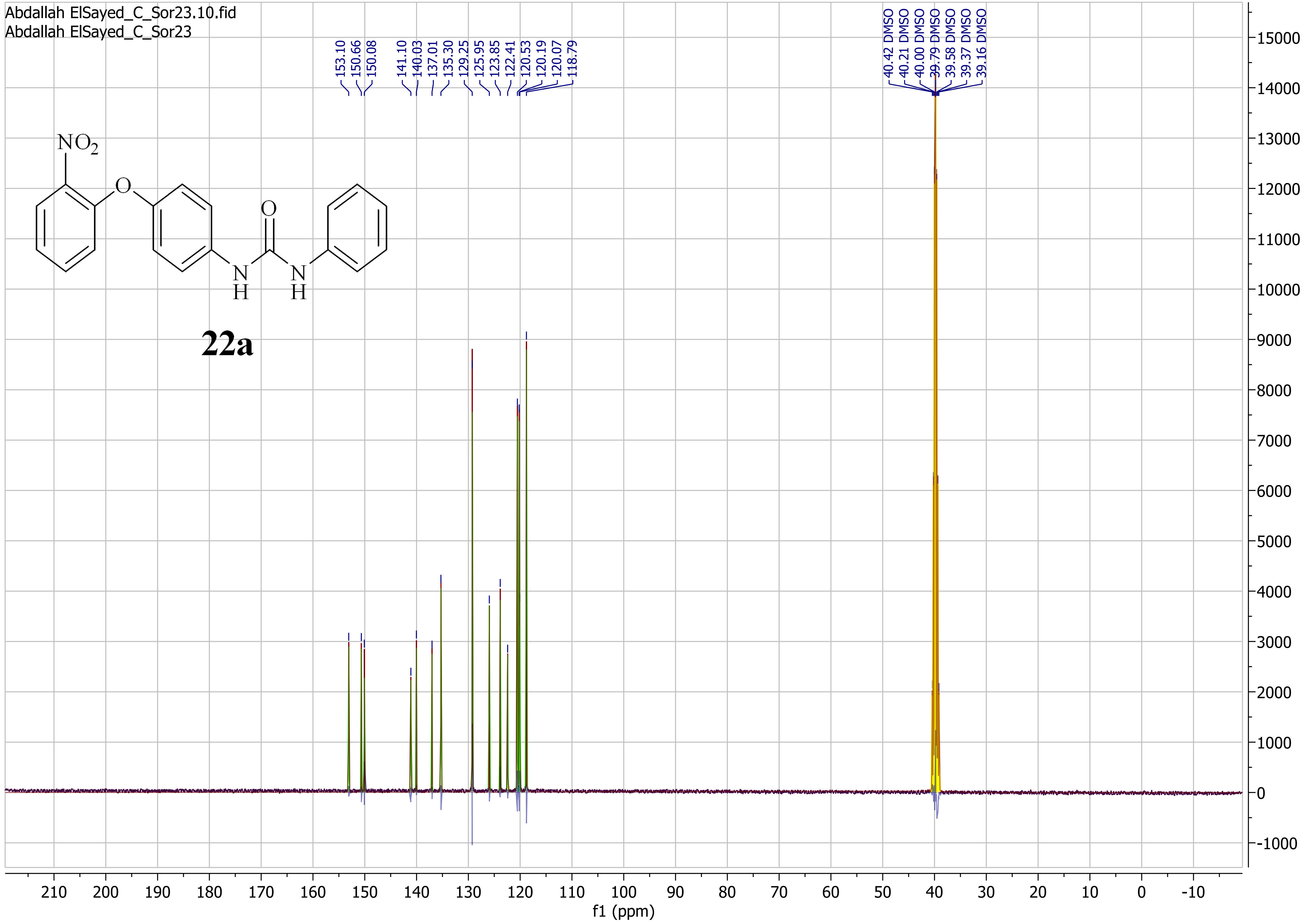
Abdallah ElSayed_C_Sor23.10.fid
Abdallah ElSayed_C_Sor23
153.10
150.66
150.08
141.10
140.03
137.01
135.30
129.25
125.95
123.85
122.41
120.53
120.19
120.07
118.79
40.42 DMSO
40.21 DMSO
40.00 DMSO
39.79 DMSO
39.58 DMSO
39.37 DMSO
39.16 DMSO
NO_2
O
O
N
H
N
H
22a
f1 (ppm)

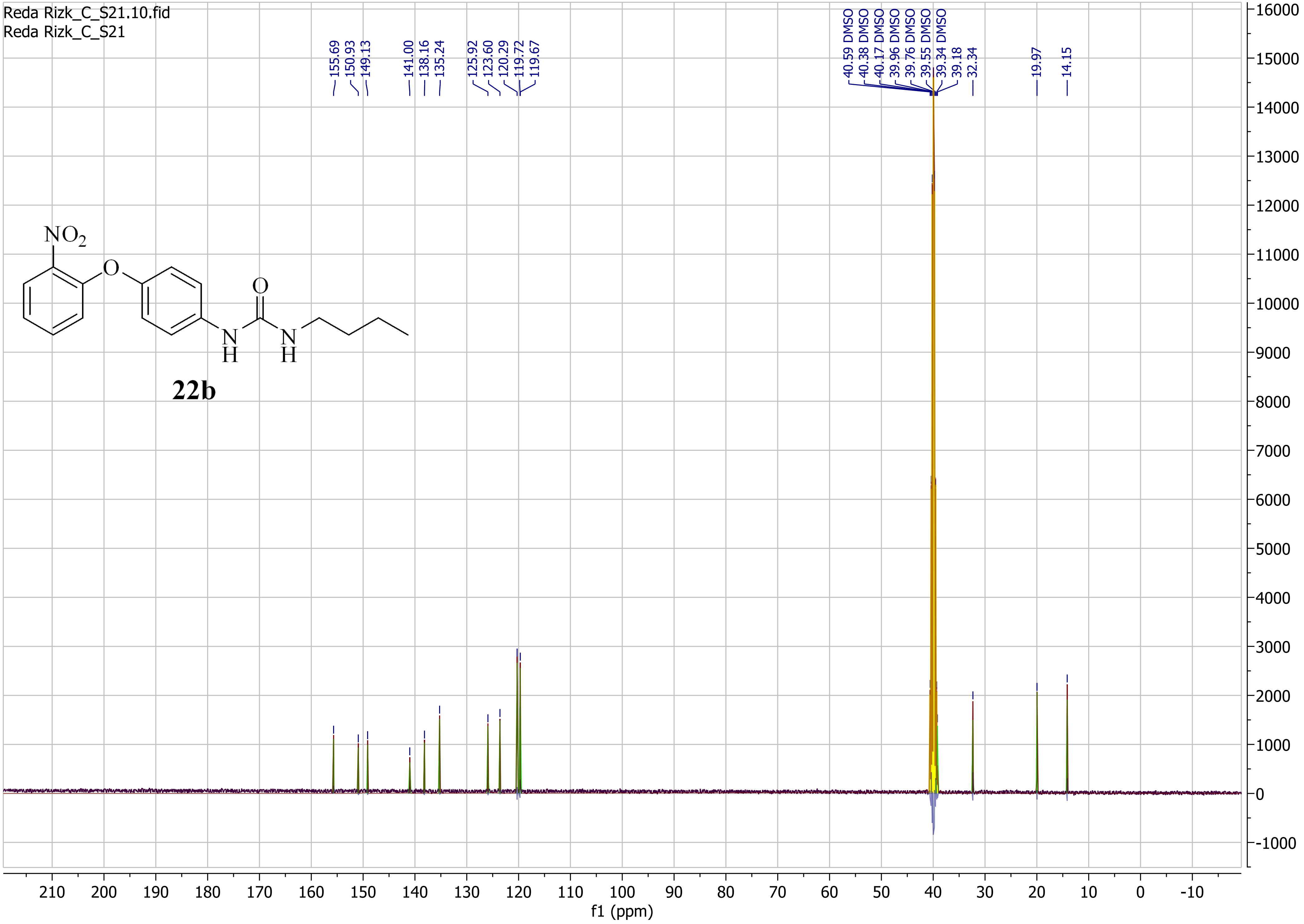
Reda Rizk_C_S21.10.fid
Reda Rizk_C_S21
155.69
150.93
149.13
141.00
138.16
135.24
125.92
123.60
120.29
119.72
119.67
40.59 DMSO
40.38 DMSO
40.17 DMSO
39.96 DMSO
39.76 DMSO
39.55 DMSO
39.34 DMSO
39.18
32.34
19.97
14.15
NO2
O
O
N
H
N
H
22b
f1 (ppm)

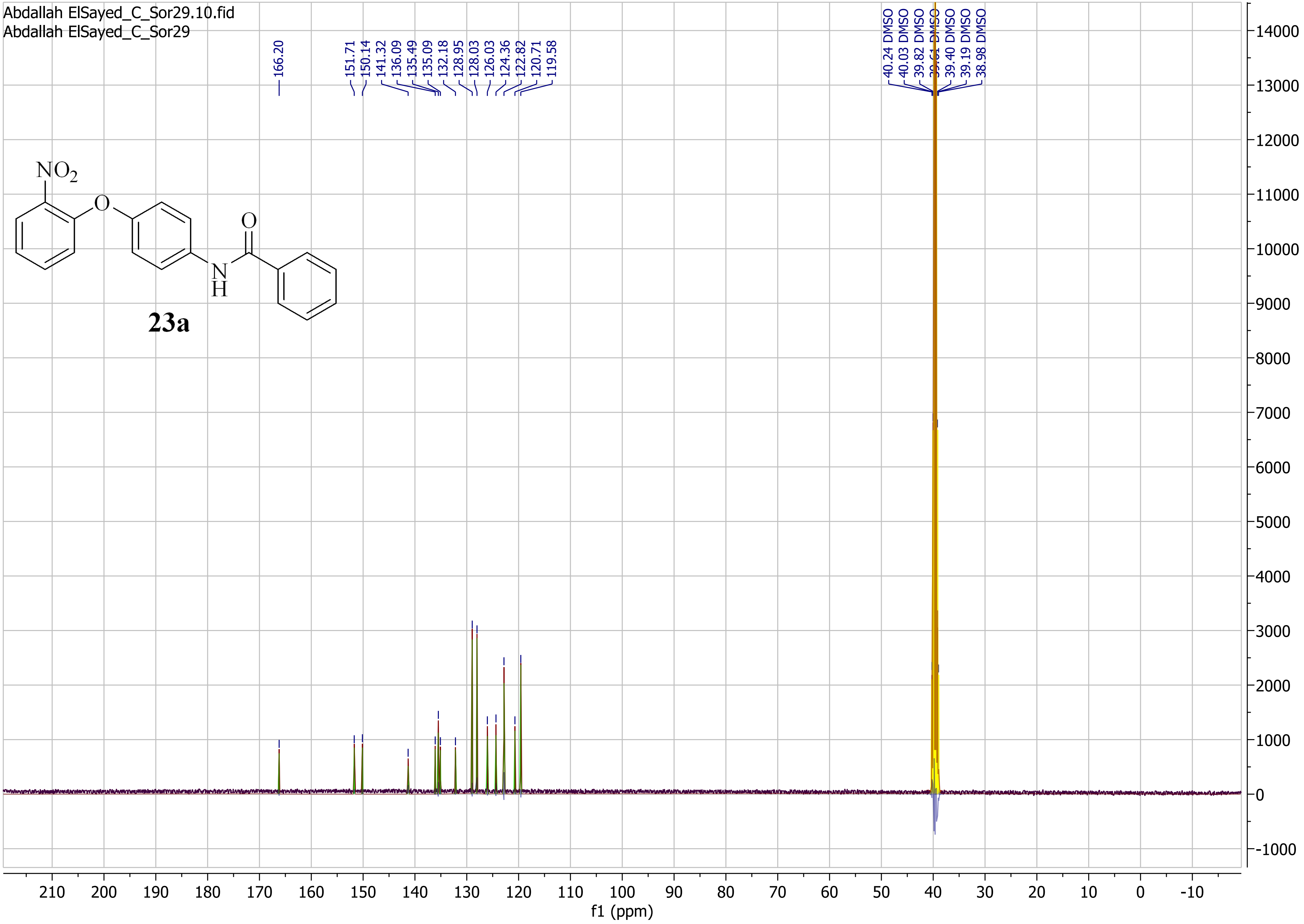

Abdallah ElSayed_C_Sor29.10.fid
Abdallah ElSayed_C_Sor29
166.20
151.71
150.14
141.32
136.09
135.49
135.09
132.18
128.95
128.03
126.03
124.36
122.82
120.71
119.58
40.24 DMSO
40.03 DMSO
39.82 DMSO
39.40 DMSO
39.19 DMSO
38.98 DMSO
NO2
O
N
H
O
23a
f1 (ppm)

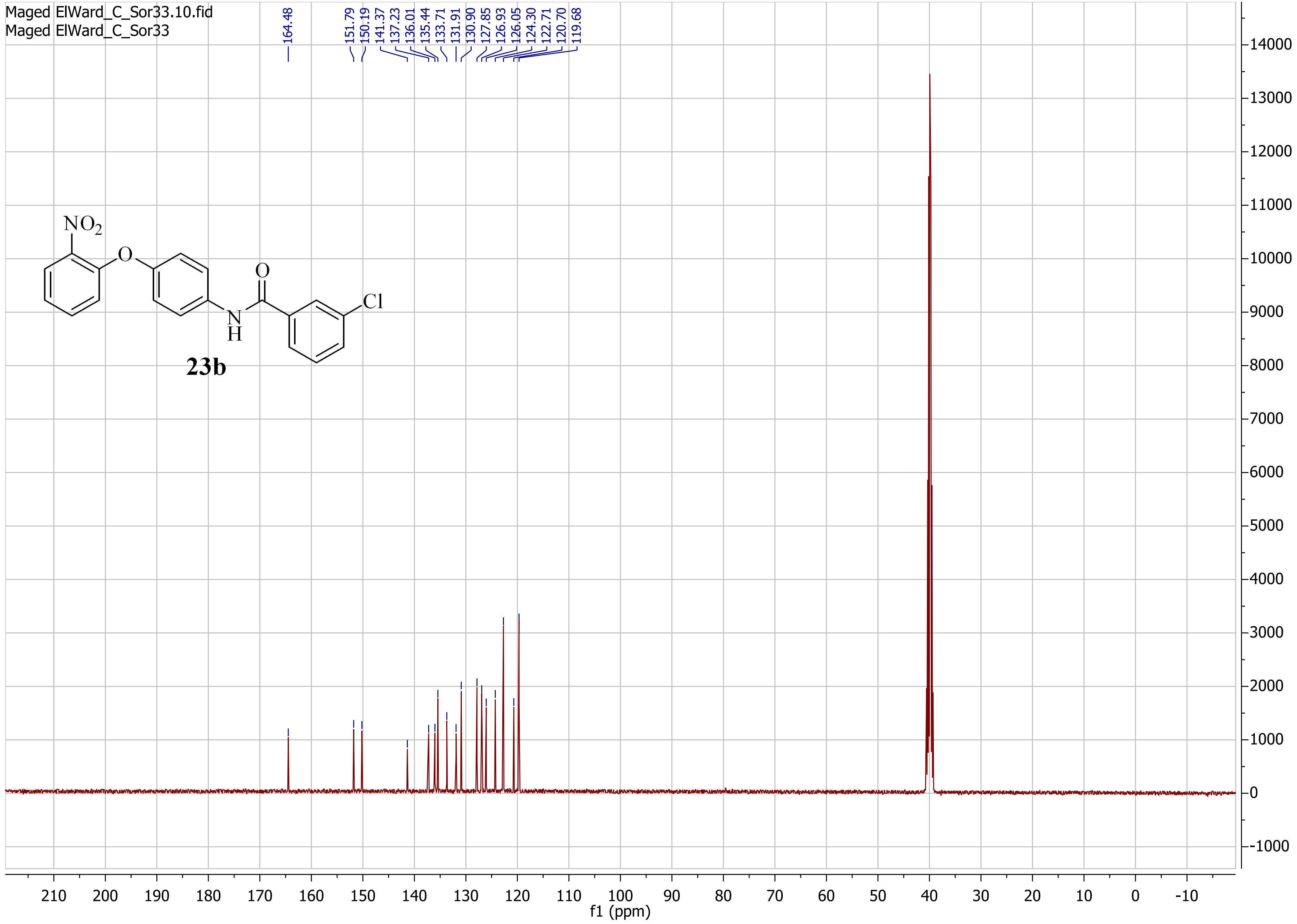

Maged ElWard_C_Sor33.10.fid
Maged ElWard_C_Sor33
164.48
151.79
150.19
141.37
137.23
136.01
135.44
133.71
131.91
130.90
127.85
126.93
126.05
124.30
122.71
120.70
119.68
NO2
O
O
Cl
N
H
23b
f1 (ppm)

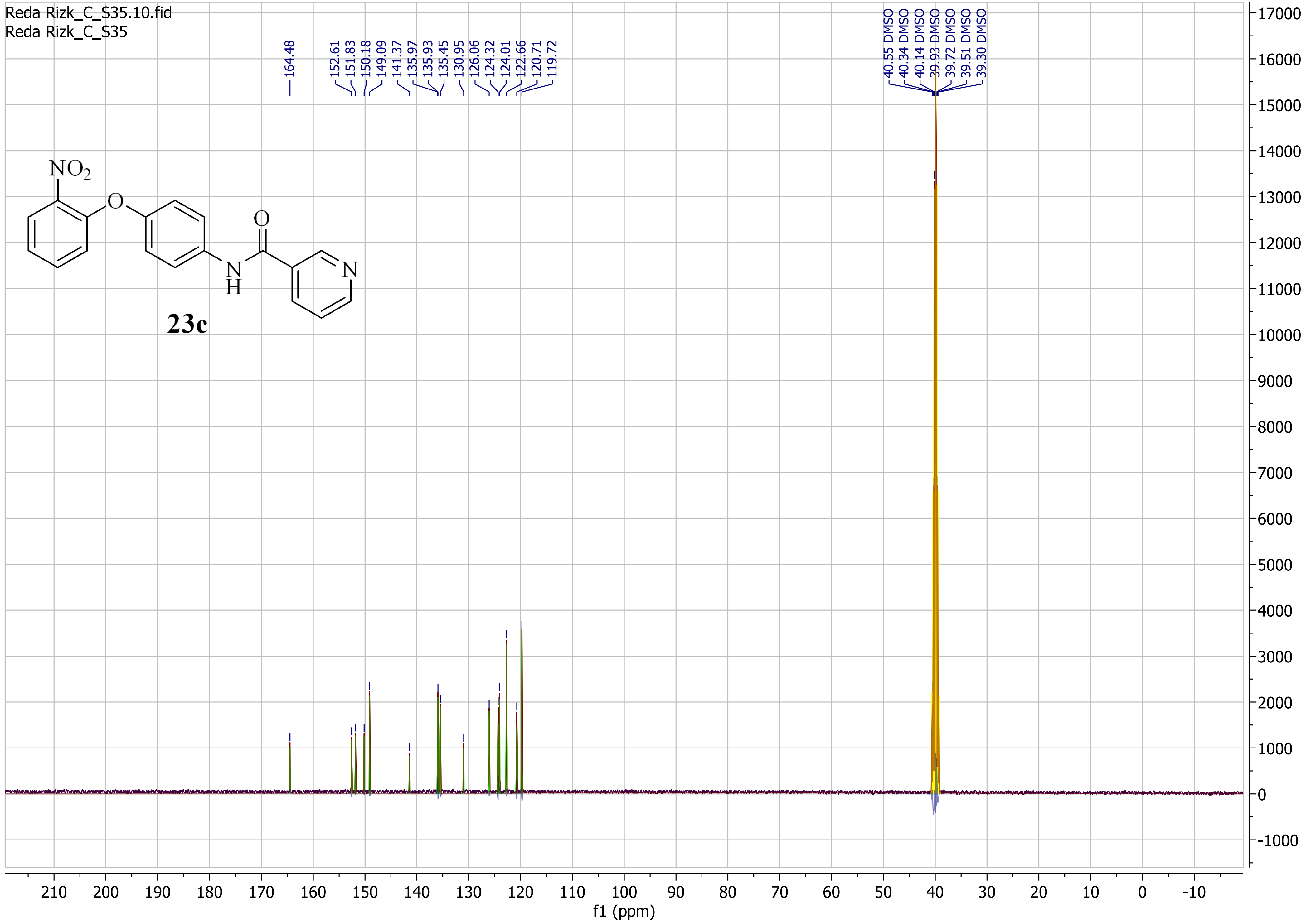
Reda Rizk_C_S35.10.fid
Reda Rizk_C_S35
164.48
152.61
151.83
150.18
149.09
141.37
135.97
135.93
135.45
130.95
126.06
124.32
124.01
122.66
120.71
119.72
40.55 DMSO
40.34 DMSO
40.14 DMSO
39.93 DMSO
39.72 DMSO
39.51 DMSO
39.30 DMSO
NO2
O
O
N
H
N
23c
17000
16000
15000
14000
13000
12000
11000
10000
9000
8000
7000
6000
5000
4000
3000
2000
1000
0
-1000
210 200 190 180 170 160 150 140 130 120 110 100 90 80 70 60 50 40 30 20 10 0 -10
f1 (ppm)

جامعة القاهرة
كلية الصيدلة

MAU
Microanalytical Unit-FOPCU
وحدة التحاليل الدقيقة
معمل الأشعة تحت الحمراء

IR Lab

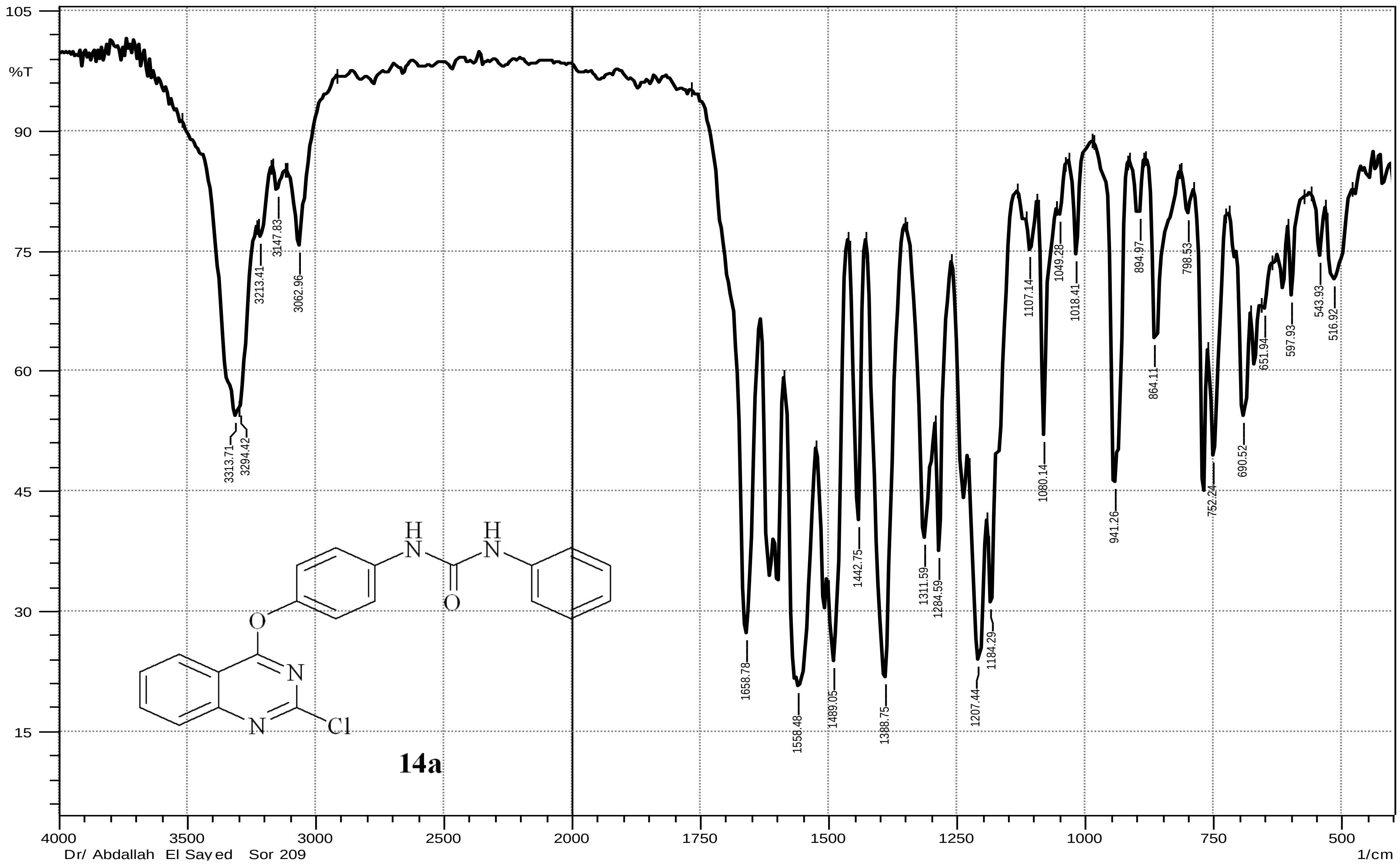

جامعة القاهرة

كلية الصيدلة

MAU

Microanalytical Unit-FOPCU

وحدة التحاليل الدقيقة

معمل الأشعة تحت الحمراء

IR Lab

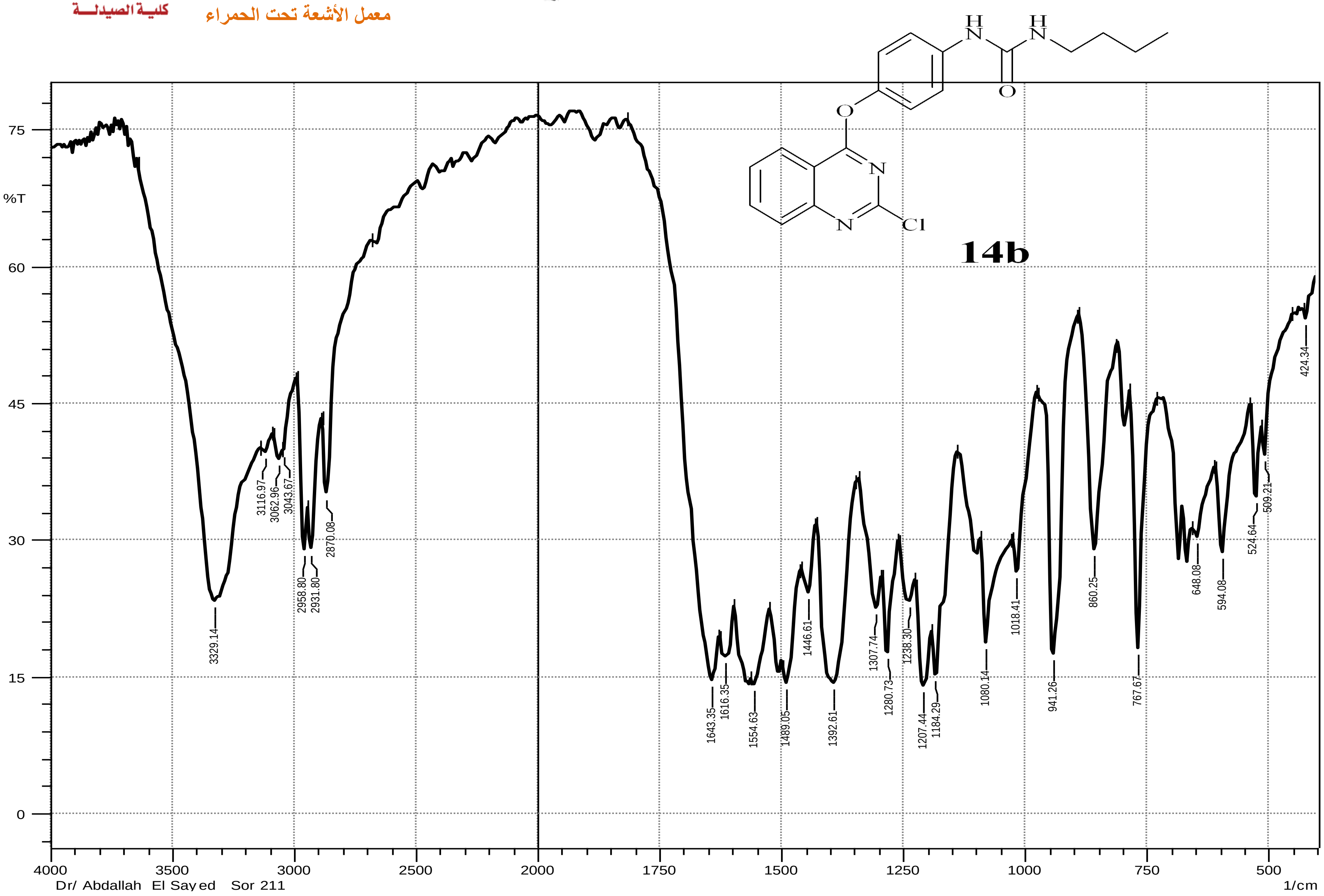

جامعة القاهرة

كلية الصيدلة

MAU

Microanalytical Unit-FOPCU

وحدة التحاليل الدقيقة

معمل الأشعة تحت الحمراء

IR Lab

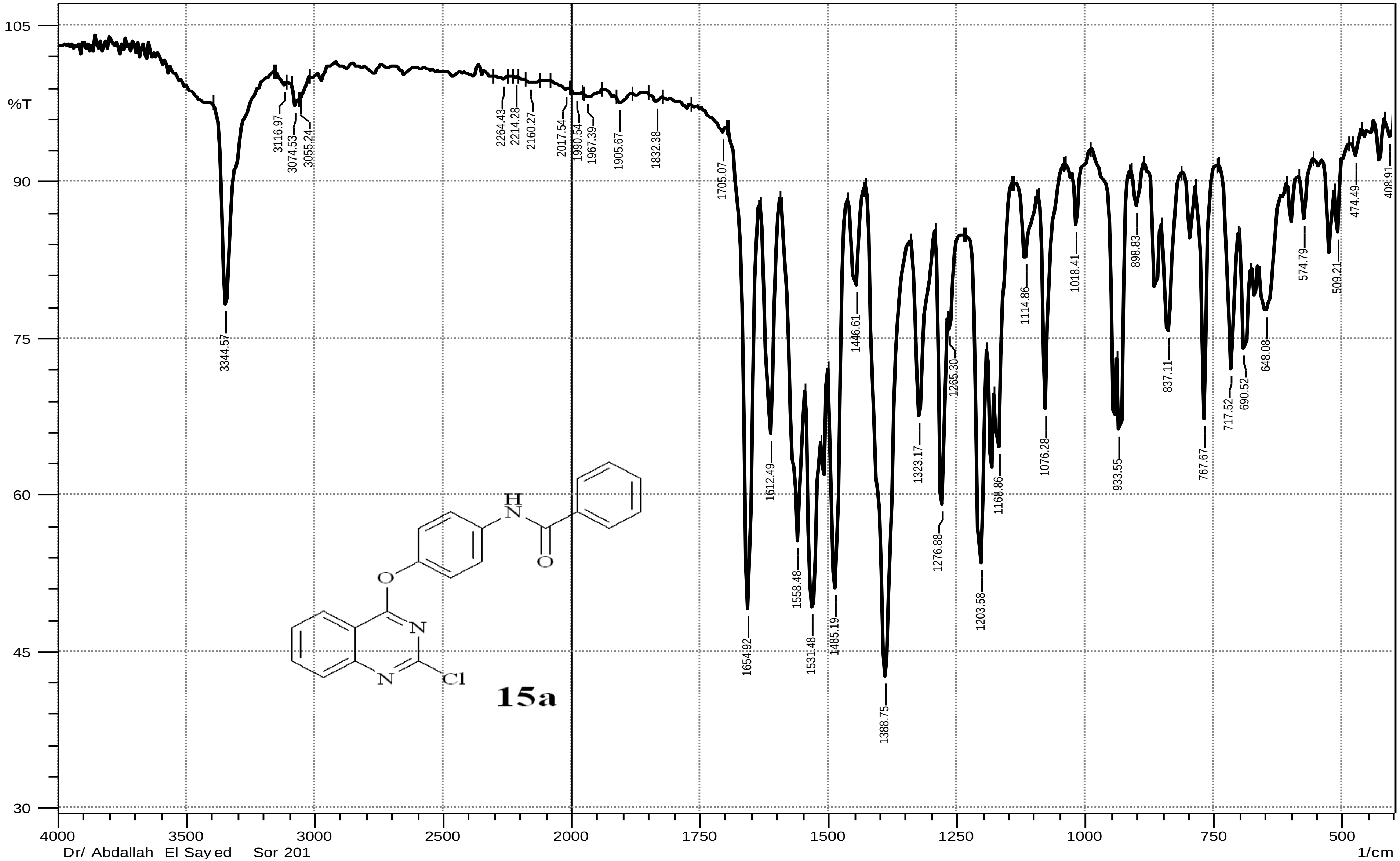

جامعة القاهرة

MAU

Microanalytical Unit-FOPCU

وحدة التحاليل الدقيقة

معمل الأشعة تحت الحمراء

كلية الصيدلة

IR Lab

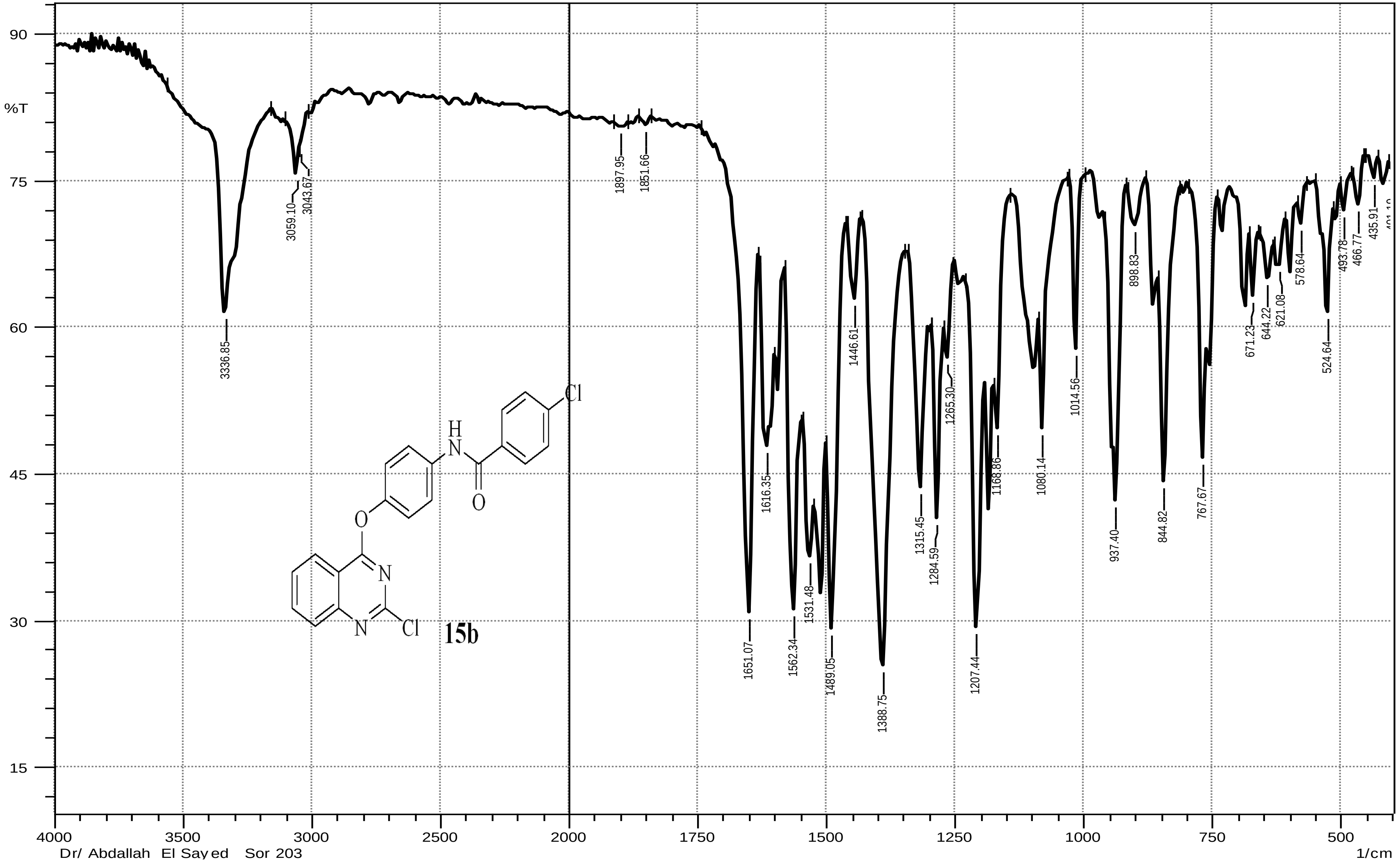

جامعة القاهرة

كلية الصيدلة

Microanalytical Unit-FOPCU

وحدة التحاليل الدقيقة

معمل الأشعة تحت الحمراء

IR Lab

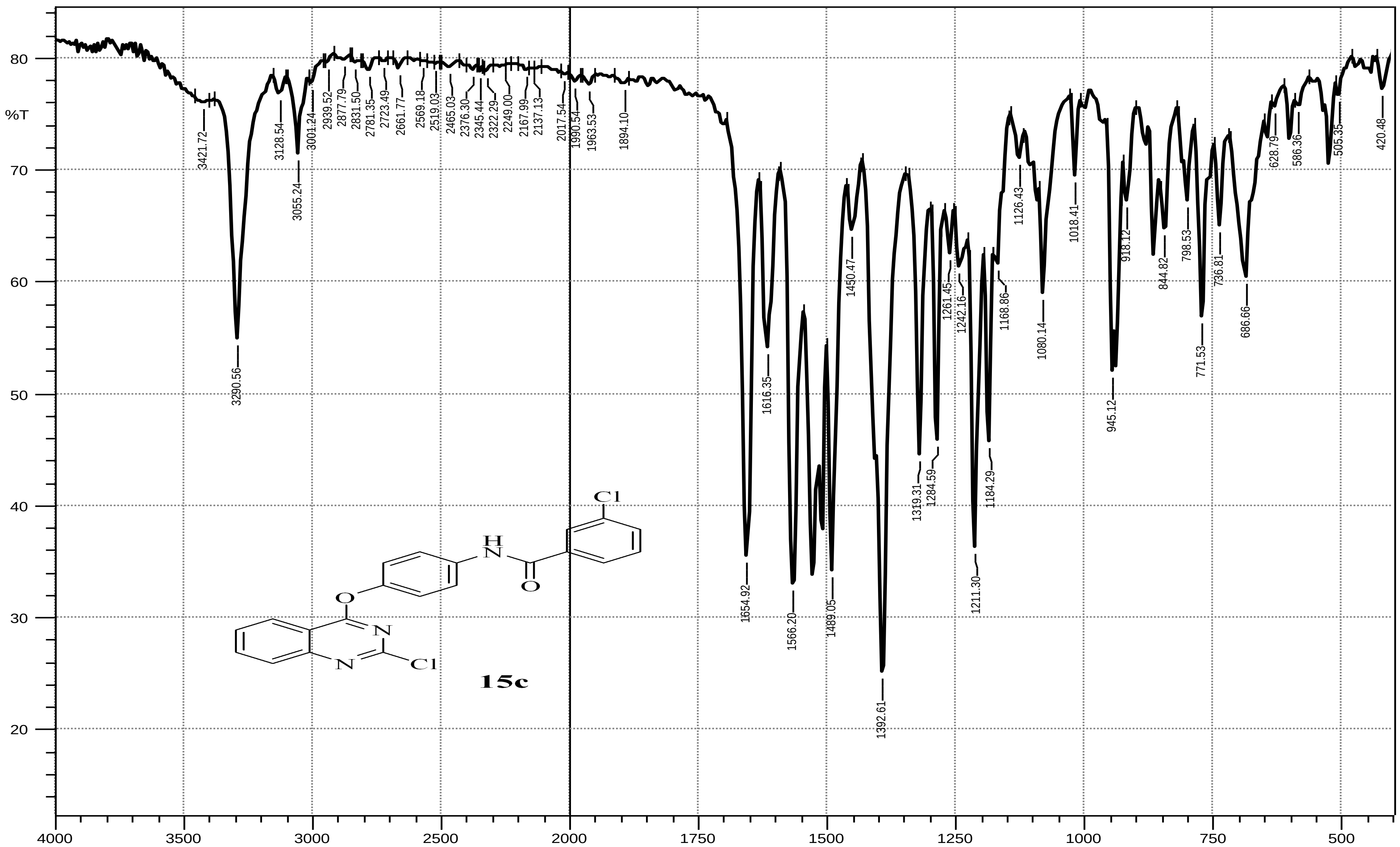

Dr/ Abdallah El Sayed Sor 205

جامعة القاهرة
كلية الصيدلة
MAU
Microanalytical Unit-FOPCU
وحدة التحاليل الدقيقة
معمل الأشعة تحت الحمراء
IR Lab

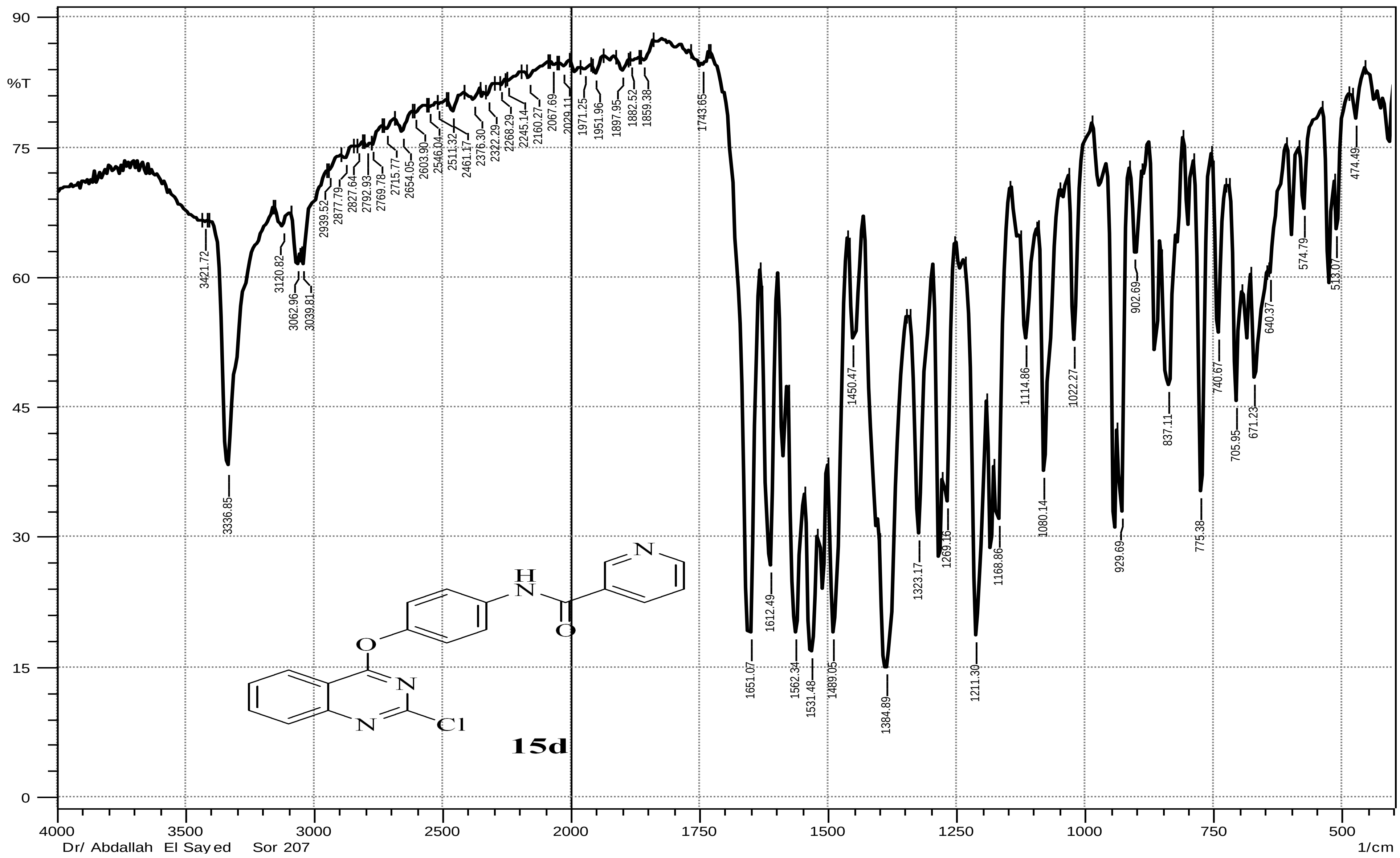

جامعة القاهرة
كلية الصيدلة

MAU
Microanalytical Unit-FOPCU
وحدة التحاليل الدقيقة
معمل الأشعة تحت الحمراء

IR Lab

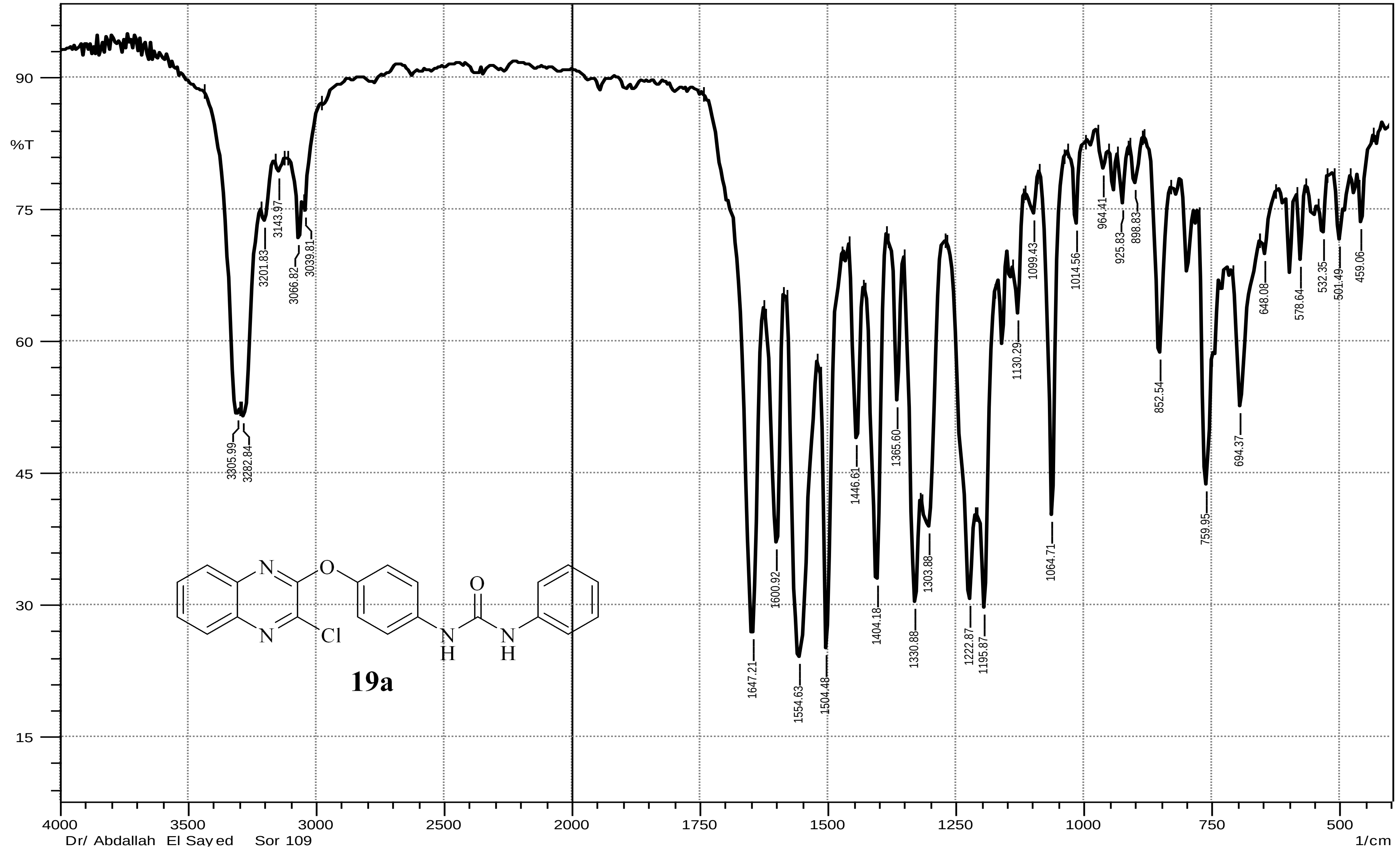

جامعة القاهرة

كلية الصيدلة

MAU

Microanalytical Unit-FOPCU

وحدة التحاليل الدقيقة

معمل الأشعة تحت الحمراء

IR Lab

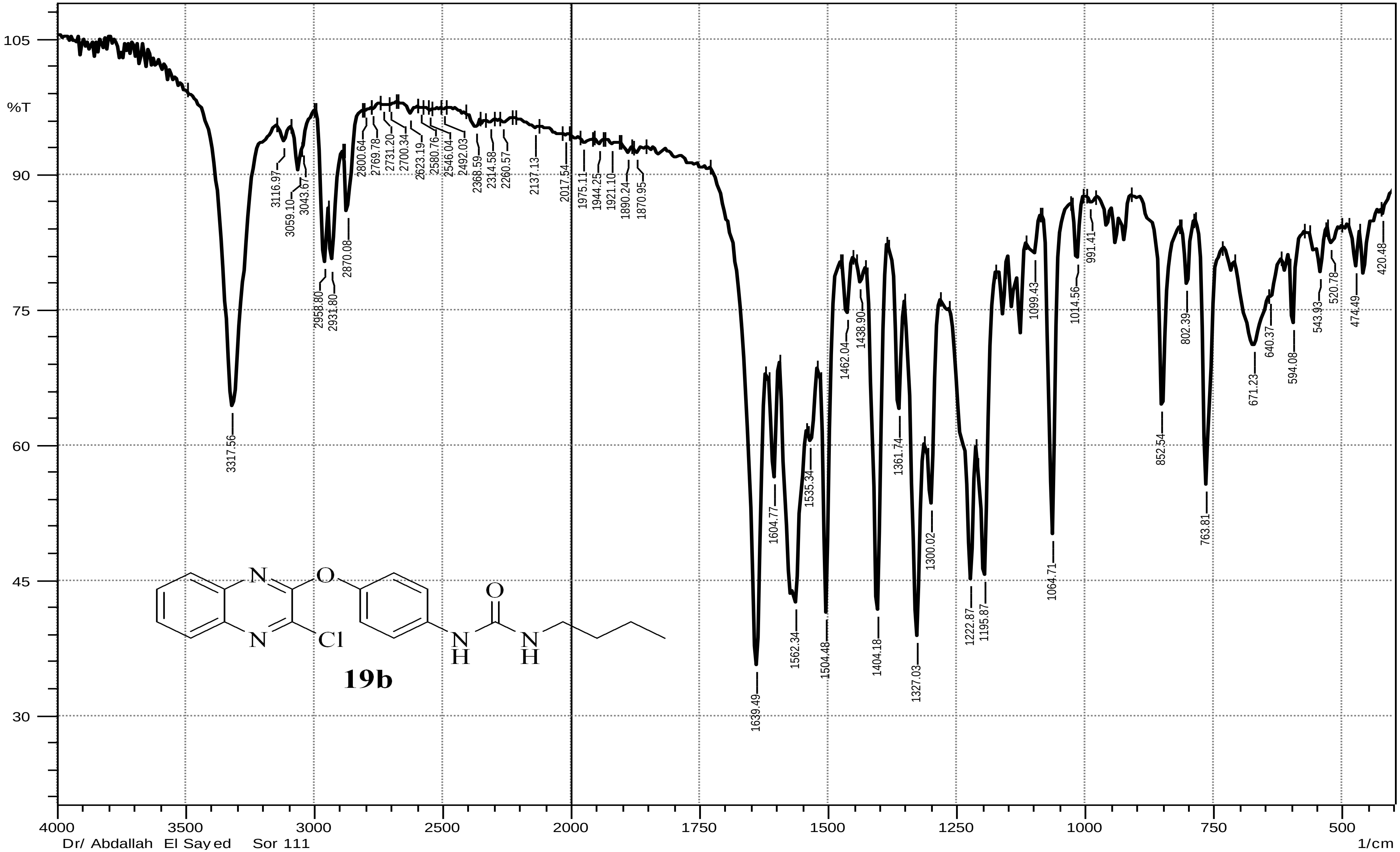

جامعة القاهرة
كلية الصيدلة

MAU
Microanalytical Unit-FOPCU
وحدة التحاليل الدقيقة
معمل الأشعة تحت الحمراء

IR Lab

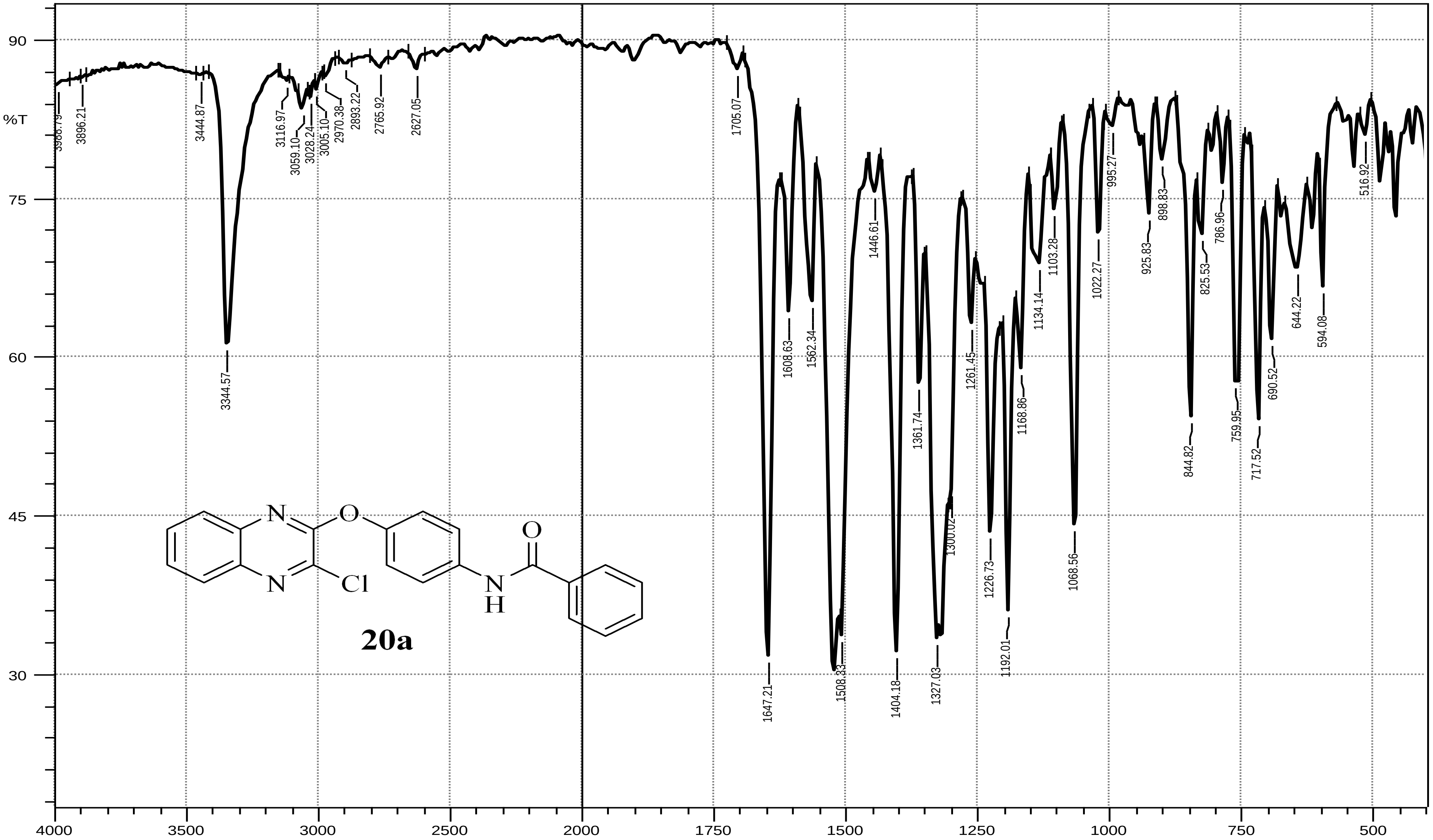

Dr/ Abdallah El Say ed Sor 101

Microanalytical Unit-FOPCU
وحدة التحاليل الدقيقة
معمل الأشعة تحت الحمراء

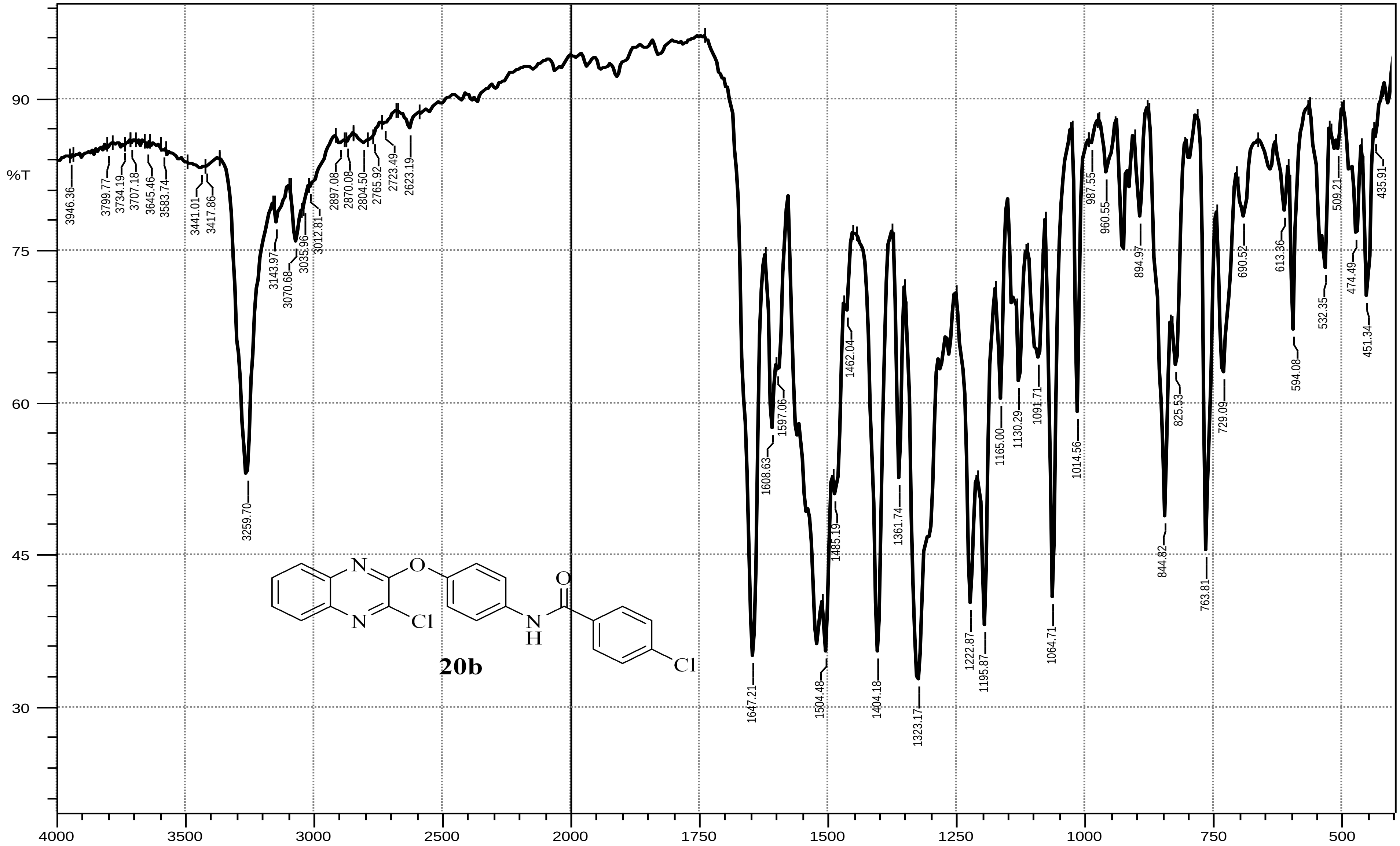

Dr/ Abdallah El Sayed Sor 103

جامعة القاهرة
كلية الصيدلة

MAU
Microanalytical Unit-FOPCU
وحدة التحاليل الدقيقة
معمل الأشعة تحت الحمراء

IR Lab

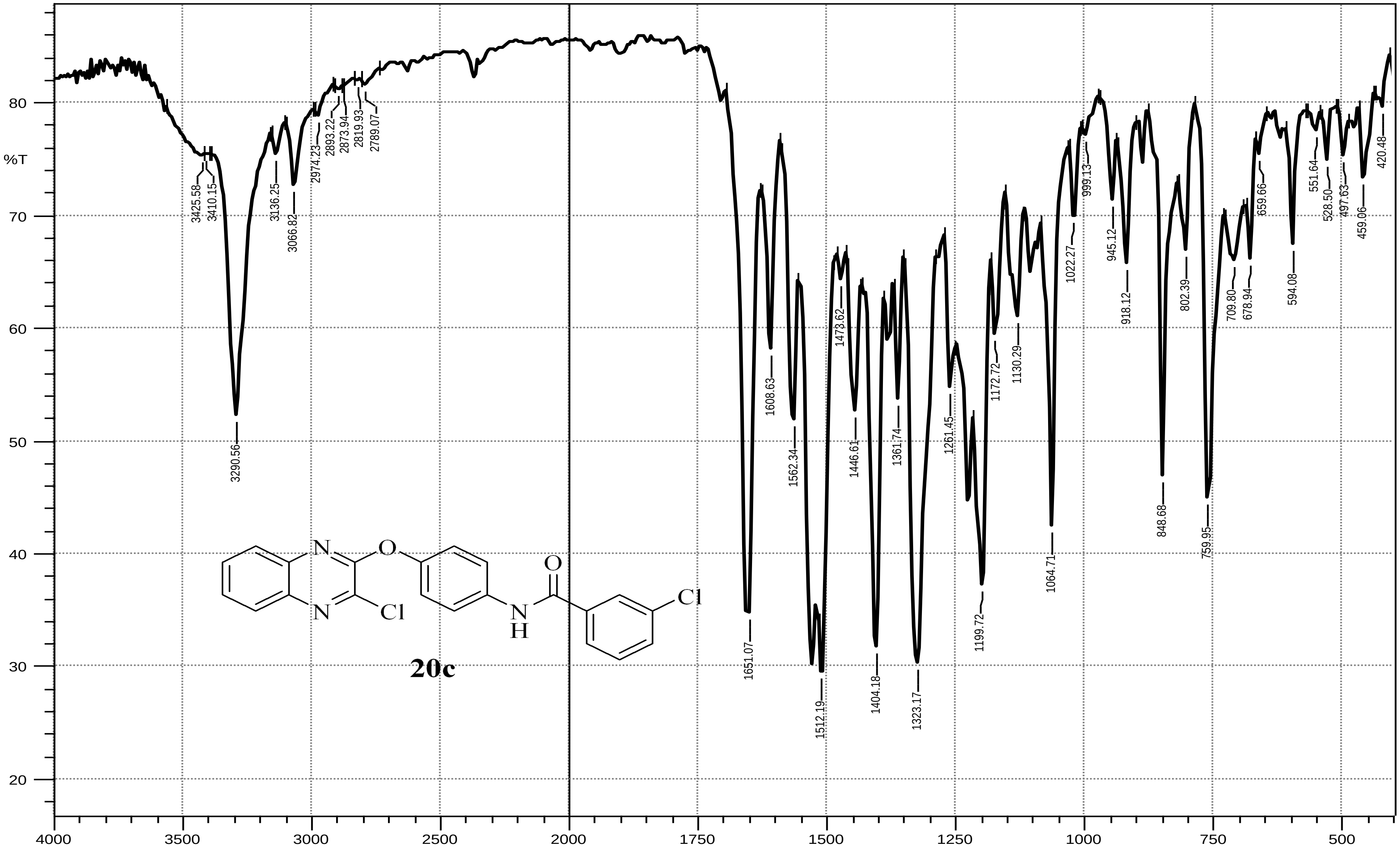

Dr/ Abdallah El Sayed Sor 105

جامعة القاهرة
كلية الصيدلة

MAU
Microanalytical Unit-FOPCU
وحدة التحاليل الدقيقة
معمل الأشعة تحت الحمراء

IR Lab

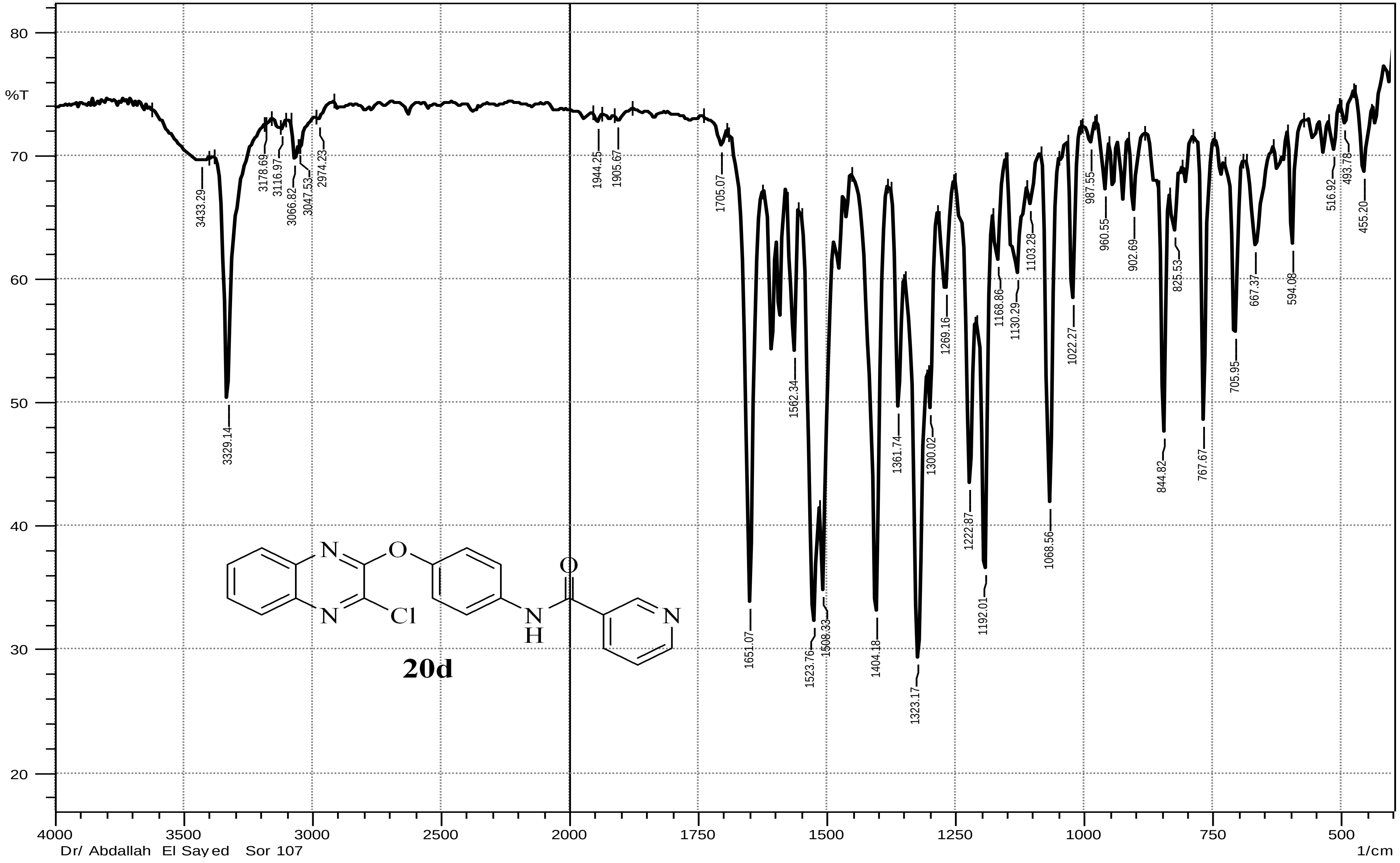

جامعة القاهرة
كلية الصيدلة

MAU
Microanalytical Unit-FOPCU
وحدة التحاليل الدقيقة
معمل الأشعة تحت الحمراء

IR Lab

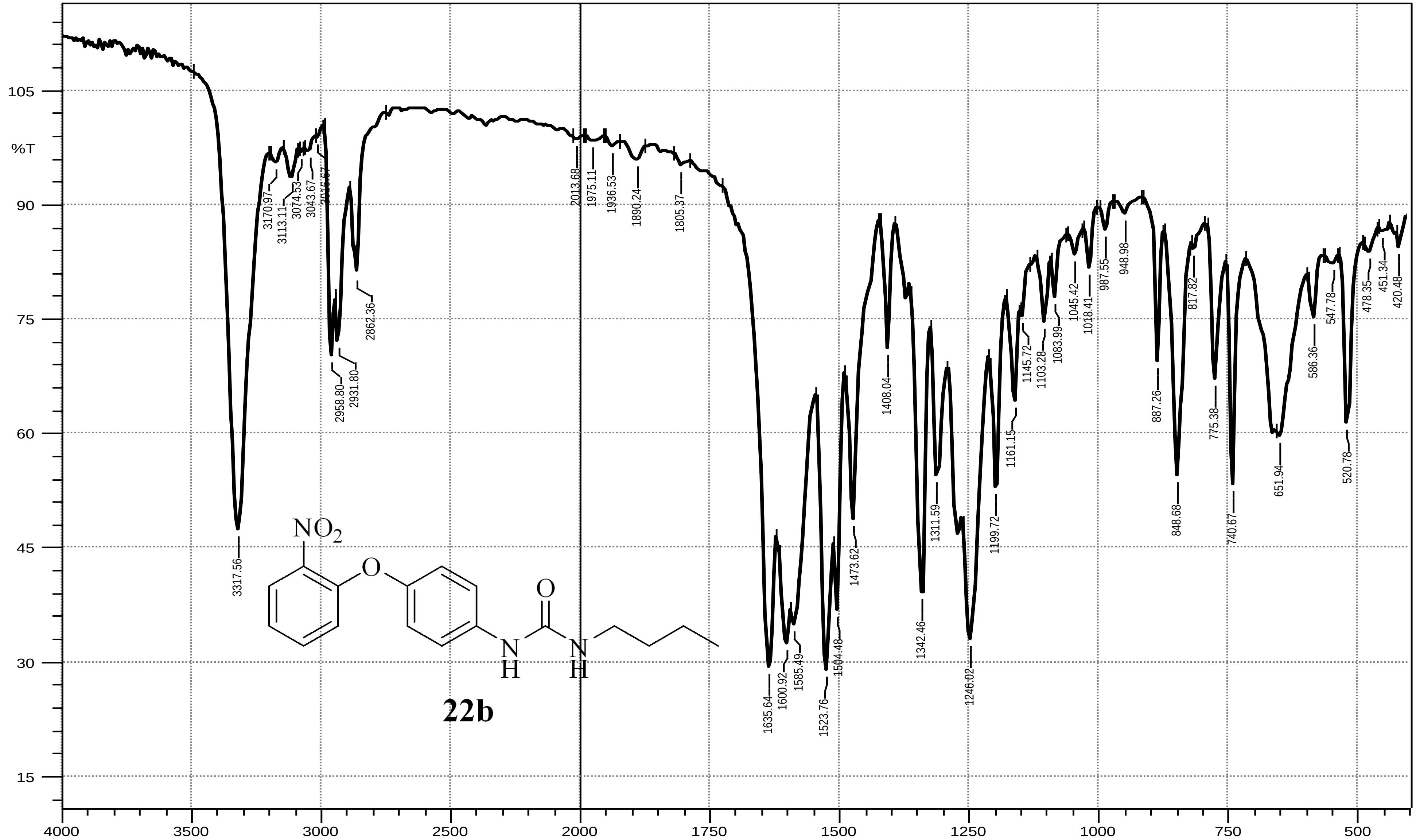

Dr/ Abdallah El Sayed Sor 21

جامعة القاهرة
كلية الصيدلة

MAU

Microanalytical Unit-FOPCU
وحدة التحاليل الدقيقة
معمل الأشعة تحت الحمراء

IR Lab

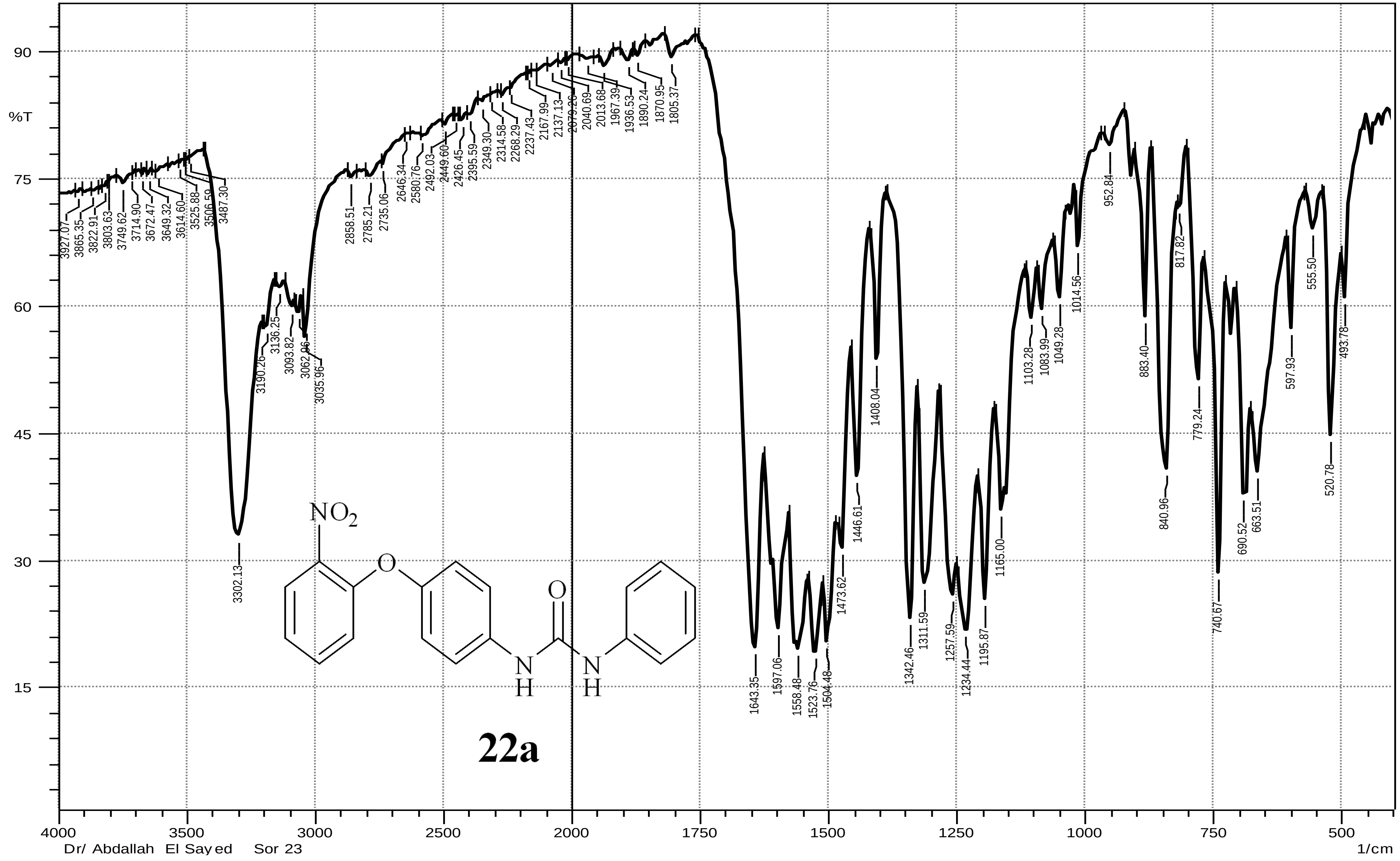

جامعة القاهرة

كلية الصيدلة

MAU

Microanalytical Unit-FOPCU

وحدة التحاليل الدقيقة

معمل الأشعة تحت الحمراء

IR Lab

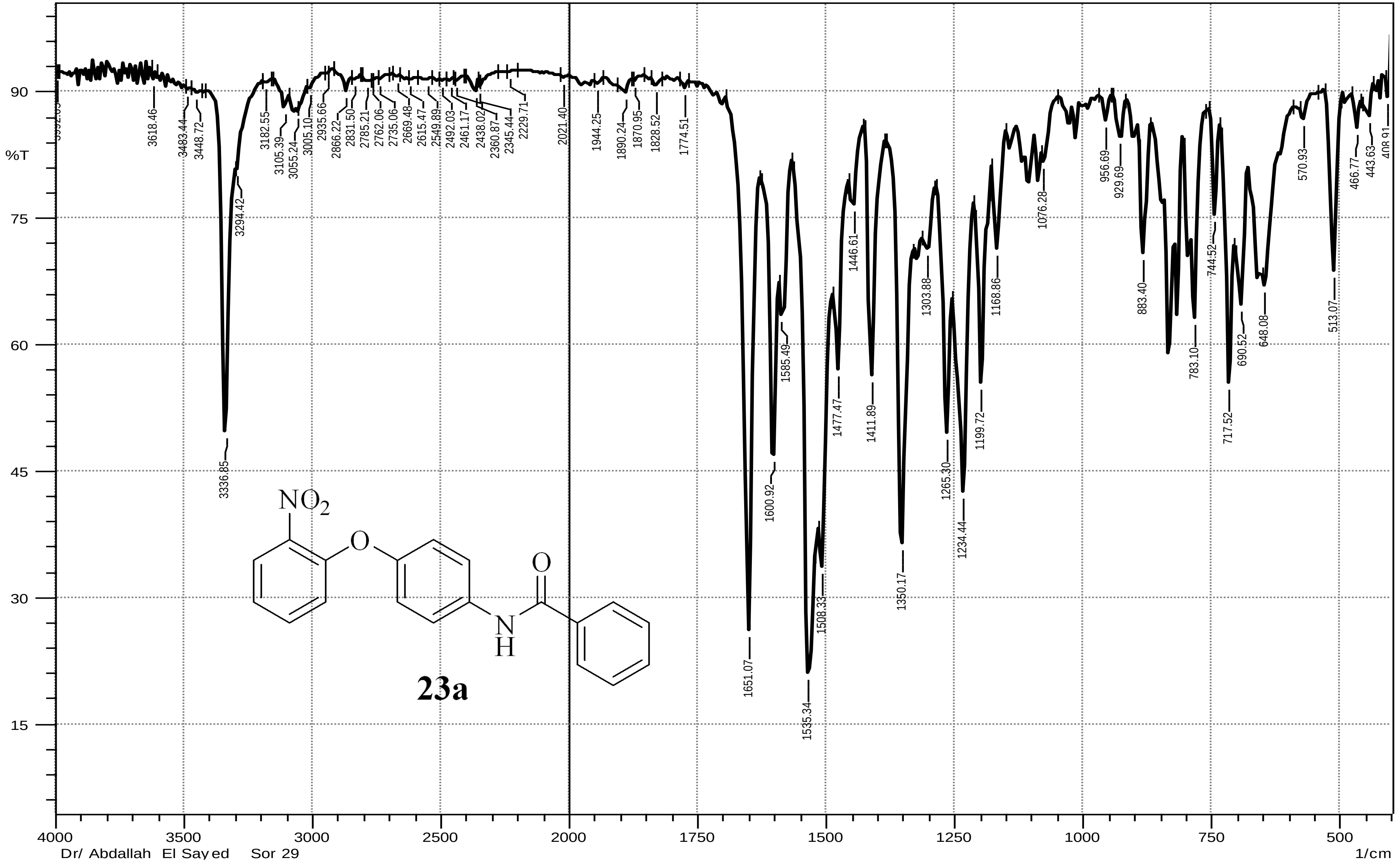

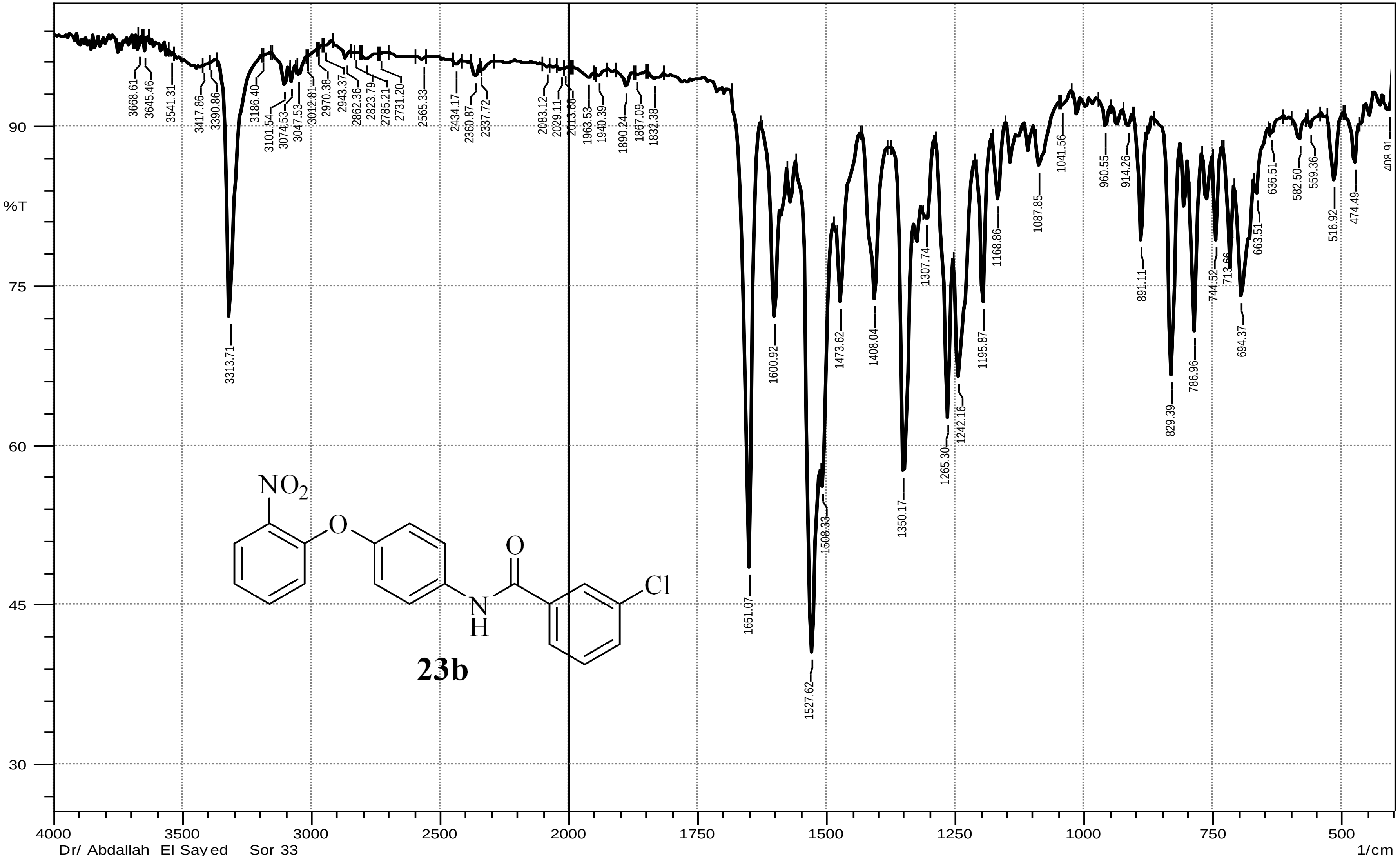

Dr/ Abdallah El Sayed Sor 33

جامعة القاهرة

كلية الصيدلة

MAU

Microanalytical Unit-FOPCU

وحدة التحاليل الدقيقة

معمل الأشعة تحت الحمراء

IR Lab

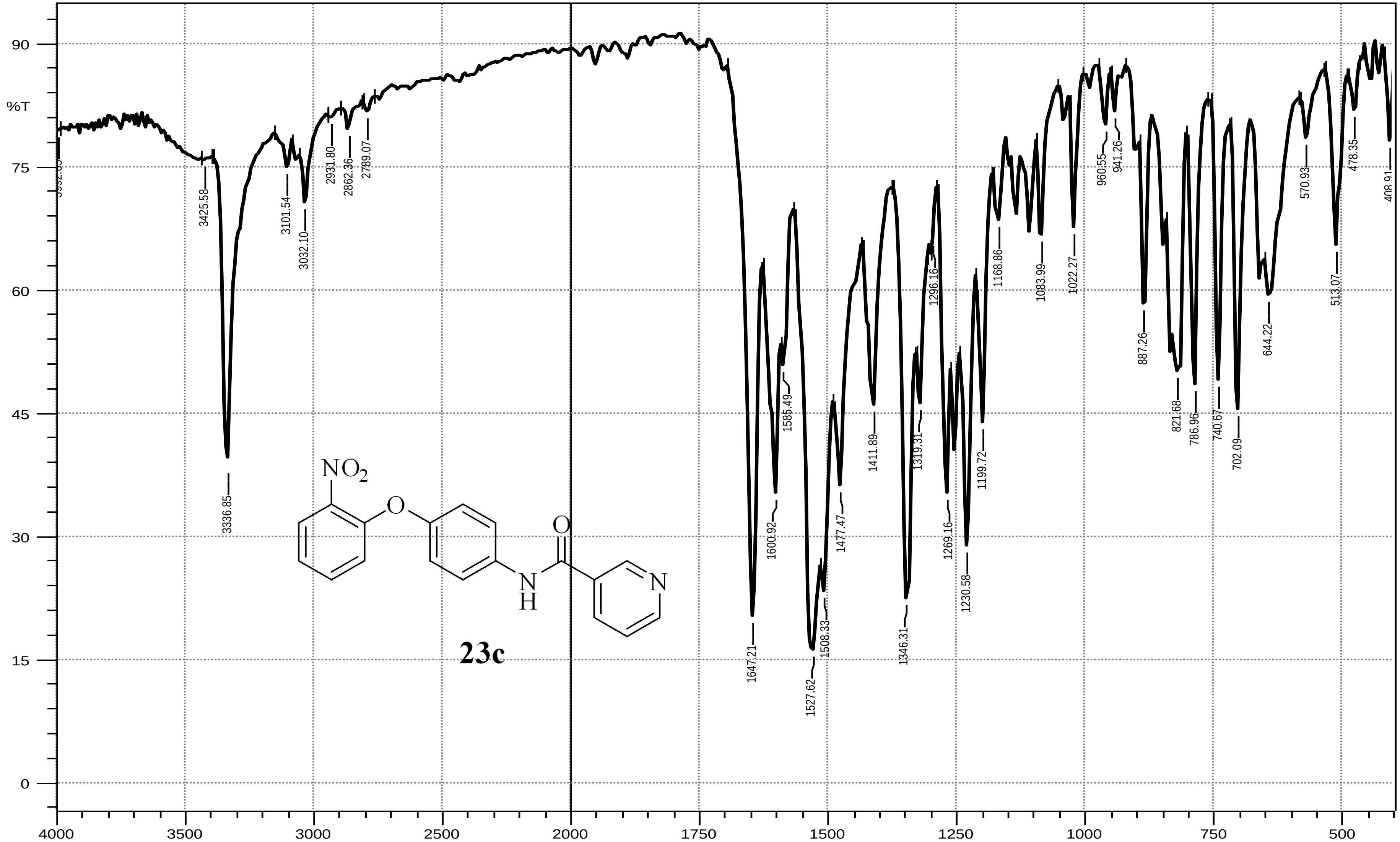

Dr/ Abdallah El Sayed Sor 35

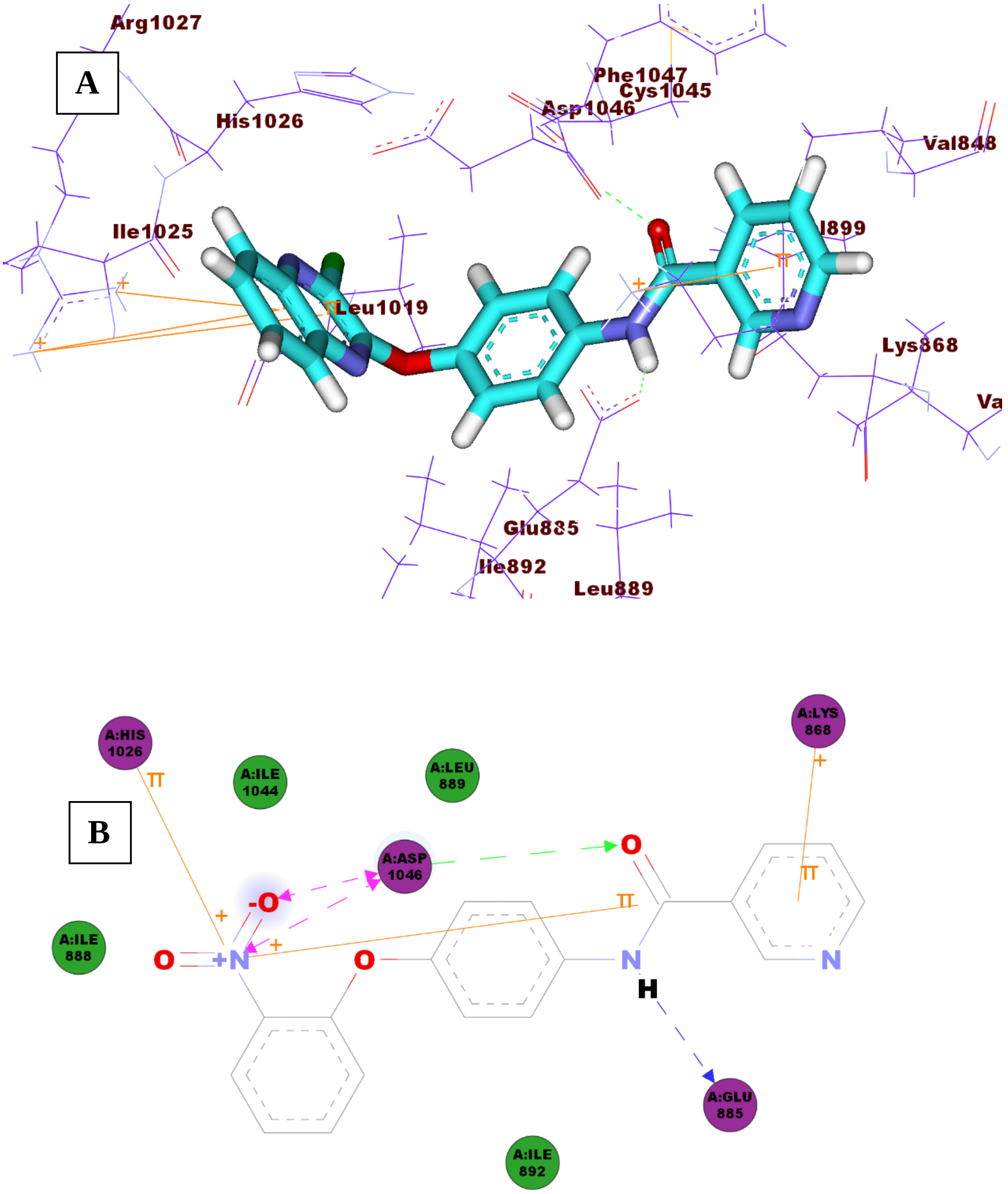

Fig. 12. (A) 3D of compound **20_d** binding pattern (B) 2D of compound **20_d** binding pattern.

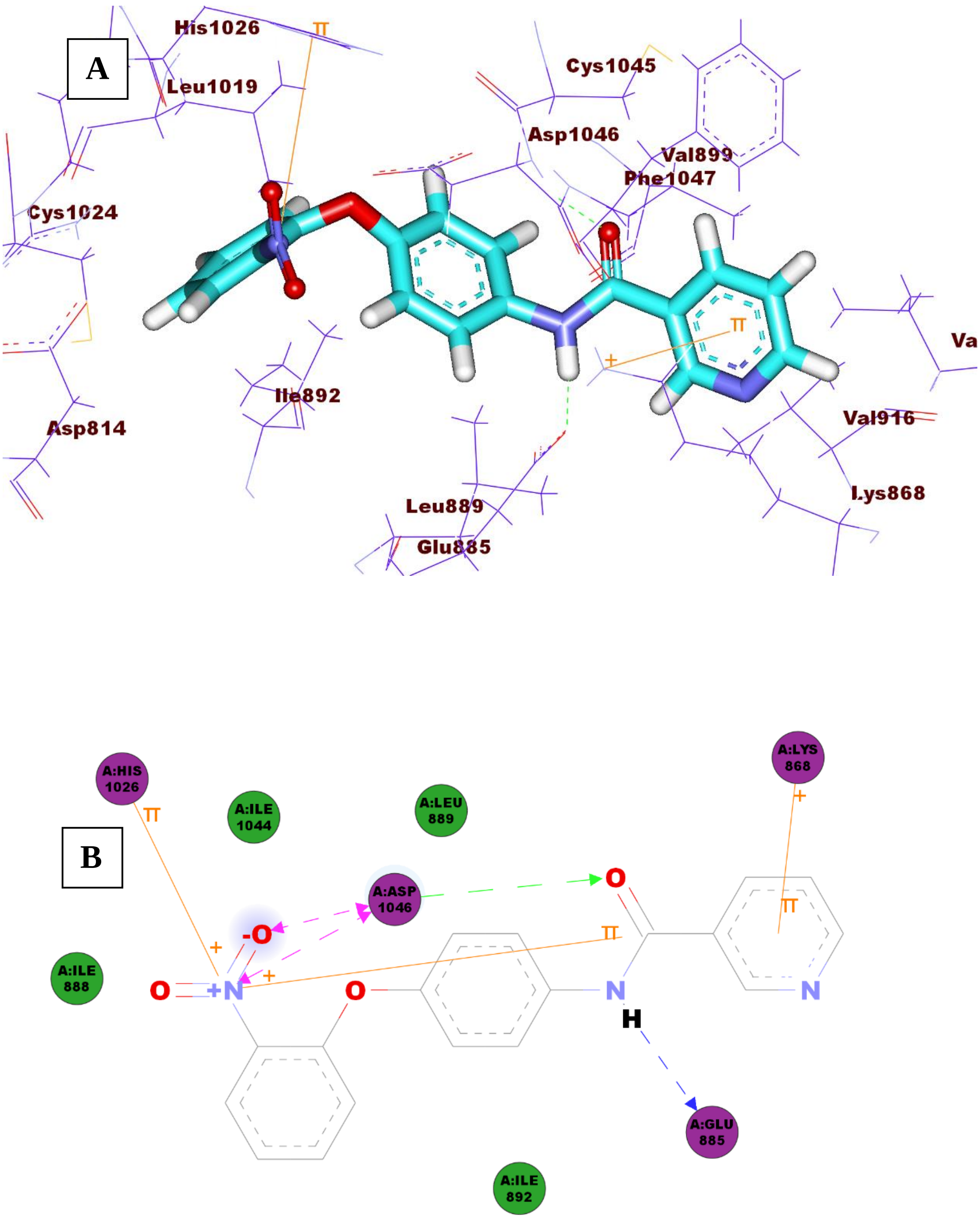

Fig. 13. (A) 3D of compound **23$_c$** binding pattern (B) 2D of compound **23$_c$** binding pattern
